# ENDOU-1-induced cytoplasmic HnRNPA3 recognizes m6A methylation on the upstream reading frame of human *CHOP* transcripts to achieve maximal *CHOP* translation

**DOI:** 10.1007/s00018-026-06180-7

**Published:** 2026-03-28

**Authors:** Hung-Chieh Lee, Yi-Hsin Huang, Chi-Cheng Hsieh, Yi-Nan Ke, Huai-Jen Tsai

**Affiliations:** 1 Department of Life Science, Fu-Jen Catholic University, No.510, Zhongzheng Rd., Xinzhuang Dist, New Taipei City, 24205 Taiwan (R.O.C.); 2 Liver Disease Prevention and Treatment Research Foundation, 6F., No.30-1, Gongyuan Rd., Zhongzheng Dist,, 100008 Taipei, Taiwan (R.O.C.)

**Keywords:** HnRNPA3, uORF, m6A, ENDOU-1, Translational control

## Abstract

Translation of the coding sequence for human C/EBP homologous protein (CHOP) is highly regulated by its upstream open reading frame (*uORF*^*chop*^). ENDOU-1 was shown to play positive roles in mediating *CHOP* translation through suppressing *uORF*^*chop*^-mediated translational inhibition (*uORF*^*chop*^-MTI) during ER stress. However, when we screened for other RNA-binding proteins putatively engaged in the same inhibitory activity, we found that HnRNPA3 associated with zebrafish Endouc and its mammalian orthologue ENDOU-1 and could also be involved in *uORF*^*chop*^-MTI in a p-eIF2α-independent manner. A time course experiment showed that the dynamic patterns of ENDOU-1, HnRNPA3 and CHOP were similarly fluctuant and positively correlated during ER stress. Moreover, overexpression of ENDOU-1 induced the increase of HnRNPA3 expression and caused a shift of HnRNPA3 from the nucleus to the cytoplasm. The resultant cytoplasmic HnRNPA3 served as a reader protein to recognize the N6-methyladenosine site on human *uORF*^*chop*^ cassette methylated by Wilms’ tumor 1-associating protein. Collectively, we suggest that HnRNPA3 is a positive effector of ENDOU-1 in an m6A-dependent manner, allowing it to reach maximal *CHOP* translation during ER stress.

**Supplementary Information:**

The online version contains supplementary material available at 10.1007/s00018-026-06180-7.

## Introduction

An upstream open reading frame (uORF) located at the 5’ UTR of mRNA encoding transcription factor CCAAT/enhancer-binding protein homologous protein (CHOP), a member of the C/EBP family, can regulate translational inhibition of the downstream main coding sequence of *CHOP* mRNA [1, 2]. Nevertheless, the phosphorylation of eukaryotic translation initiation factor 2 subunit α (eIF2α) can facilitate ribosomal bypass of this inhibitory uORF element, thereby allowing *CHOP* translation [3]. However, we found that the zebrafish uridylate-specific endoribonuclease c (Endouc) and its mammalian orthologue ENDOU-1 (Endouc/ENDOU-1) play important roles in the translation of human *CHOP* during endoplasmic reticulum (ER) stress through binding the human *uORF*^*chop*^ (*huORF*^*chop*^) transcript and cleaving it at the uridylate-specific residue 80G‐81U to disrupt the inhibitory effect mediated by *huORF*^*chop*^ [4]. Still, it is plausible that some unknown protein(s) associated with Endouc/ENDOU-1 could also be involved in the repression of *huORF*^*chop*^-mediated translation inhibition (*huORF*^*chop*^‐MTI) during ER stress.

To identify whether other RNA-binding proteins (RBPs) associated with Endouc might also be involved in *huORF*^*chop*^-MTI, we first applied immunoprecipitation (IP) and LC-MS/MS analyses to screen putative Endouc-interacting RBPs, followed by overexpressing the candidates to examine those that could facilitate *CHOP* translation. One of four screened RBPs was heterogeneous nuclear ribonucleoprotein A3 (HnRNPA3). It stood out among the four by displaying the highest affinity in the Co-immunoprecipitation (Co-IP) with Endouc/ENDOU-1. Therefore, this study aimed to investigate (1) the biological function of HnRNPA3 and (2) the effect of the HnRNPA3, which interacts with Endouc/ENDOU-1, on regulating of *CHOP* mRNA translation during ER stress.

HnRNPs are a class of RBPs that share many structural and functional similarities [5]. The structures of more than 20 HnRNPs have been identified. Generally, HnRNPs contain several core functional domains responsible for mRNA processing, transport, transcription, DNA repair, and telomere maintenance through DNA-, RNA-protein or protein-protein interactions [5–7]. Although HnRNPs can interact with RNA motifs in a sequence-specific manner, nonspecific interactions with RNA are also prevalent among HnRNPs [8–10]. Dysfunction, nuclear depletion, and the formation of cytoplasmic inclusions can lead to abnormal transcription and translation of a range of downstream target RNAs [11]. Therefore, HnRNPs play important roles in regulating gene expression.

The HnRNPA/B subfamily is the core member of HnRNPs. It mainly consists of four isoforms, including HnRNPA0, A1, A2/B1, and HnRNPA3, that perform multiple functions in mRNA nuclear import and pre-mRNA alternative splicing, as well as cytoplasmic trafficking of mRNA stability, turnover, and translation [8, 12]. Among these four isoforms within the hnRNPA/B subfamily, HnRNPA3 is relatively less studied. So far, however, accumulated evidence reveals that HnRNPA3 plays important roles in cell senescence, differentiation, cancer, and neurodegeneration. For example, HnRNPA3 is involved in the senescence of human diploid fibroblasts [13]. Intronic polyadenylation is dynamically regulated by HnRNPA3 to influence epidermal differentiation gene expression [14]. HnRNPA3 has been proposed as a valuable differential diagnostic and prognostic biomarker during the multistep processes of hepatocellular carcinoma carcinogenesis; Upregulated HnRNPA3 is tightly associated with poor survival of patients with hepatocellular carcinoma [15, 16]. Increased HnRNPA3 plays roles in bladder cancer cisplatin resistance [17] and glioblastoma Temozolomide resistance [18]. Interestingly, HnRNPA3 is associated with chromosome 9 open reading frame 72 (C9orf72)-mediated frontotemporal lobar degeneration (FTLD)/amyotrophic lateral sclerosis (ALS) pathology through its specific binding to mutant C9orf72 repeat RNA [19], and binds with dipeptide repeat proteins [20, 21]. Although extensive studies have reported on the functions of HnRNPA3 in human pathogenesis, its roles during ER stress remain unknown.

In this paper, we identified an ENDOU-1-interacting protein, HnRNPA3, and found that ENDOU-1 induces the cytoplasmic translocation of HnRNPA3 which, in turn, allows it to directly bind the N6-methyladenosine (m6A)-2 methylation site on the *uORF*^*chop*^ transcript and thus act as a positive effector to increase translation of the main open reading framez (mORF) of CHOP protein, effectively allowing ENDOU-1 to achieve maximal abrogation of *uORF*^*chop*^-MTI in a p-eIF2α-independent manner during ER stress.

## Materials and methods

### Zebrafish

Zebrafish (*Danio rerio*) wild-type (Wt) AB strain (RRID: ZIRC_ZL1) and transgenic line *huORFZ* [22] were cultured as previously described [4]. The experiments and treatments of this zebrafish model have been reviewed and approved by the Fu Jen Catholic University Institutional Animal Care and Use Committee with ethics approval number A11065. Microinjection of zebrafish embryos at the one-celled stage followed the protocol described previously [22].

### Cell culture

HEK293T, HeLa, HepG2, and NSC34 cells were cultured and transfected as previously described [2, 4, 23]. Insect cells Sf21 were cultured and transfected as described by Fu et al. [24]. HEK293T and HeLa cells were provided by Prof. Sheng-Chung Lee, National Taiwan University. NSC-34 cells were obtained from Cedarlane Laboratories (Cedarlane, Burlington, ON, Canada, Cat. no. CLU140). HepG2 cells were provided by Prof. Jin-Chuan Sheu, Liver Disease Prevention and Treatment Research Foundation. HEK293T, HepG2, and NSC34 cells were all maintained in DMEM high glucose (Simply) plus 10% fetal bovine serum (Gibco) and 1% penicillin/streptomycin (Gibco) at 37°C under 5% CO_2_. Insect cell line Sf21 provided by Prof. Yen-Ling Song, National Taiwan University, was maintained in Grace’s Insect Medium (Gibco) plus 10% fetal bovine serum at 28°C under 5% CO_2_.

For drug treatment, cultures were replaced by fresh medium, incubated for 2 h, and the following chemicals were individually added: Dimethyl sulfoxide (DMSO; Sigma-Aldrich, D2650), which served as the control group, 1 µM Thapsigargin (TH, Sigma-Aldrich, T9033), which served as an ER stress inducer. Cells were then harvested after treatment for 1–8 h, as indicated.

### Cell transfection

HEK293T and HeLa cells were sub-cultured in wells of a 6-well plate starting with a density of about 1 × 10^5^ cells/well for 24 h until cells were at 80–90% confluence. Transfection of recombinant protein expression plasmids was performed using Lipofectamine™3000 transfection reagent (ThermoFisher) according to the manufacturer’s instructions, while transfection of siRNA or siRNA/expression plasmids was performed using Lipofectamine 2000 Transfection Reagent (ThermoFisher) according to the manufacturer’s instructions. For dual-luciferase (dual-luc) assay, HEK293T cells were plated in wells of a 6-well plate with 70% confluence. The Firefly-reporter plasmids and phRG-TK (internal control) were co-transfected using jetPRIME transfection reagent (Polyplus) according to the manufacturer’s instructions. Luciferase (luc) activity was measured using the Dual-Glo luciferase assay kit (Promega, E2920).

All siRNAs were designed and synthesized by Santa Cruz. The siRNAs used in this study were HnRNPA3 siRNA (sc-38262), ENDOU-1 siRNA (PP11 siRNA; sc-95700), METTL14 siRNA (sc-89054), WTAP siRNA (sc-63224), and Control siRNA-A (sc-37007).

### Plasmid construction

The puORF^chop^-luc and phRG‐TK reporter constructs, zebrafish Endouc-Flag, and human ENDOU-1 expression vectors were described previously [4, 22]. The cDNAs encoding human SREK1-interacting protein 1 (SREK1IP1), Serine/arginine-rich splicing factor 9 (Srsf9), Protein transport protein Sect. 61 subunit alpha 1 (Sec61α1) and HnRNPA3 were cloned by RT–PCR from the cDNA pools to generate the following Myc-tagged recombinant protein expression vectors: pSREK1IP1-Myc, pSrsf9-Myc, pSec61α1-Myc, and pHnRNPA3-Myc. Flag-tagged HnRNPA3 expression vector was generated using the same strategy. Plasmid mCherry‐HnRNPA3 fusion constructs were provided from Prof. Kohji Mori [21]. We inserted a strong NLS from simian virus 40 (SV40) large tumor antigen (T-ag) into the mCh-A3ΔM9 mutant to generate mCh‐A3NLS vector. The m6A-2 site was located in the *huORF*^*chop*^. Thus, to analyze the influence of m6A modification on *huORF*^*chop*^, we mutated the m6A-2 site using PCR mutagenesis and inserted it into the pCDNA3-luc vector to generate puORF^chop^-AAU mut-luc and puORF^chop^-AGC mut-luc. The m6A-1 site was located in the 5’UTR of *CHOP* transcript before the *huORF*^*chop*^. Thus, a DNA fragment containing *CHOP* 5’UTR including *huORF*^*chop*^ was inserted into pCDNA3-luc to generate puORF^chop^-m6A-3-Luc. The m6A-1 mutant was the m6A-motif AGACT mutated to AGTCT using PCR mutagenesis and inserted into the pCDNA3-luc to generate puORF^chop^-m6A-1-mut-Luc.

The m6A-3 site was located in the 5’UTR of *CHOP* transcript between the *huORF*^*chop*^ and the *CHOP* coding sequence. Thus, a DNA fragment containing *huORF*^*chop*^ and 5’UTR sequence between the *huORF*^*chop*^ and *CHOP* coding sequence was inserted into pCDNA3-luc to generate puORF^chop^-m6A-3-Luc. The m6A-3 mutant was the m6A-motif AGACT mutated to AGTCT using PCR mutagenesis and inserted into pCDNA3-luc to generate puORF^chop^-m6A-3-mut-Luc. Primers used for these constructs were list in Table S2.

### In-gel digestion and LC-MS/MS analysis

The recombinant zebrafish Endouc-Flag protein was produced using insect cell Sf21 [4]. After 4 d, the infected Sf21 cells were collected for protein extraction. The recombinant Endouc-Flag protein and whole protein extracts from HEK293T cells were purified using IP lysis buffer (ThermoFisher #87787) supplemented with Protease Inhibitor Cocktail Tablets (Roche, 04693132001) and then incubated with 30 µL of anti-FLAG M2-agarose beads (Sigma-Aldrich, A2220) overnight with gentle rocking. After washing 6 times with the IP lysis buffer, Flag-tagged proteins were eluted with 0.5 mg/mL of 3 x Flag peptide (GenScript) in the lysis buffer at 4°C for 30 min. Next, the IP products shown on 10% SDS-PAGE after silver staining were cut into five gel slices about 1 mm in length. Then, these gel slices were applied to perform in-gel digestion and LC-MS/MS, using the Mascot Distiller (Matrix Science). The resultant MGF file was searched using the Mascot Search Engine (v2.2, Matrix Science) according to the standard protocol described by Fu et al., [23]. Candidate genes were listed in Table S1.

### Western blotting and immunostaining

Western blot analysis followed the procedures described previously [25]. The following antibodies were used: CHOP (1:1000; CST, #2895), HnRNPA3 (1:1000; Proteintech, 25142-1-AP), ATF4 (1:1000; Proteintech, 10835-1-AP), eIF2α (1:1,000; CST, #5324); p-eIF2α (1:1,000; CST, #3398); PERK (1:1,000; CST, #3192); p‐PERK (1:1,000; Abcam, Ab156919); Bip (1:1,000; Proteintech, 11587-1-AP); Flag (1:20,000; Proteintech, 20543-1-AP); Myc(1:1,000; CST, #2276); ENDOU‐1 (1:1,000; Abcam, EPR15137); m6A (1:1000; Proteintech, 68055-1-Ig), METTL14 (1:1,000; Sigma-Aldrich, HPA038002), WTAP (1:1,000; Proteintech, 10200-1-AP), Lamin B1 (1:1000; Santa Cruz, sc-374015), GAPDH (1:20000; Abcam, Ab181602), α‐tubulin (1∶10,000; Sigma-Aldrich, T6074), Multi-rAb™ HRP-Goat Anti-Mouse Recombinant Secondary Antibody (1:20,000; Proteinech, RGAM001) and Multi-rAb™ HRP-Goat Anti-Rabbit Recombinant Secondary Antibody (1:20000; Proteintech, RGAR001).

To optimize CHOP staining, the membrane was incubated with 0.5% (v/v) glutaraldehyde in TBST (20 mM Tris pH7.4, 150 mM NaCl, 0.1% Tween 20) for 5 min and then washed at least 6 times with TBST buffer before blocking with 5% BSA/TBST solution [26]. Protein extracts of 20 ~ 50 µg were loaded in each lane to detect the protein level.

### Immunoprecipitation (IP)

HEK293T cells were transfected with expression vectors encoding the respective proteins (pEndouc-Flag, pENDOU-1-Flag, pSREK1IP1-Myc, pSrsf9-Myc, pSec61α**1**-Myc, and pHnRNPA3-Myc) for 48 h. Cells were washed with ice-cold phosphate-buffered saline (PBS: 137 mM NaCl, 27 mM KCl, 15 mM KH_2_PO_4_, 81 mM Na_2_HPO_4_) and lysed using IP lysis buffer (ThermoFisher, #87787) supplemented with Protease Inhibitor Cocktail tablets (Roche, 04693132001) at 4°C for 12 min. The cleared lysate was incubated overnight with 20 µL of anti-Flag beads (Sigma-Aldrich, A2220). After washing 6 times with IP lysis buffer, Flag-tagged protein complexes were eluted with 0.5 mg/mL of 3 x FLAG peptide (Genscript) in the lysis buffer at 4°C for 30 min with gentle rocking, separated on 10% SDS-polyacrylamide gels, and analyzed by immunoblotting with the indicated antibodies.

### RNA-Electrophoretic Mobility Shift Assay (RNA-EMSA)

Recombinant HnRNPA3-Myc protein was incubated with biotin-labeled RNA substrate under suitable binding conditions (40 mM Tris, pH7.4, 30 mM KCl, 1 mM MgCl_2_, 0.1% IGEPAL^®^ CA-630, 1 mM DTT, and 10 µg/ml BSA) at 37°C for 30 min. The results were analyzed on 15% polyacrylamide gel containing 8 M urea and stained with the Chemiluminescent Nucleic Acid Detection Module kit (ThermoFisher, #89880). For biotin-labeled RNA synthesis, full-length *huORF*^*chop*^ RNA (105 nt) and luc 105 bp RNA were synthesized using the T7 Transcription kit (Invitrogen, AM1344). Two pmole of template were used, and the reaction lasted 5 h. Then, these small RNAs were labeled with biotin using the RNA 3′ End Biotinylation kit (ThermoFisher, #20160). Primers used to amplify the templates were listed in Table S2. The biotin-labeled *huORF*^*chop*^ template DNA was synthesized by Mdbio Bioscience. For purification of HnRNPA3-Myc protein, HEK293T cells were transiently transfected with pHnRNPA3-Myc expression vector for 48 h and lysed using IP lysis buffer (ThermoFisher, #87787) supplemented with Protease Inhibitor Cocktail tablets (Roche, 04693132001). Then lysates were immunopurified by using 30 µL of anti-c-Myc Magnetic Beads (ThermoFisher, 88843). After incubation at 4℃ overnight, beads were washed 6 times with IP lysis buffer and eluted using c-Myc peptide (M2435; Sigma-Aldrich). The eluted protein samples were subjected to Western blotting.

### RNA pull-down assay

HEK293T cells were transiently transfected with pHnRNPA3-Myc expression plasmid for 48 h, washed with ice-cold PBS, and lysed in lysis buffer (25 mM Tris, pH7.4, 150 mM NaCl, 1 mM EDTA, 1% IGEPAL^®^ CA-630, 5% glycerol, and 1% TritonX100 supplemented with Protease Inhibitor Cocktail tablets (Roche, 04693132001). Protein concentrations in lysis buffer were measured with a protein assay dye reagent (Bio-Rad, #5000006). 500 ng of protein extracts were incubated with 2 µg of biotinylated probes and 100U RNasin (Promega, N2111) at 4℃ for 1 h with gentle rocking. Then, 20 µL of Streptavidin beads (ThermoFisher, 20359) were added into the RNA-protein mixtures and incubated at 4℃ for 3 h with gentle rocking. After incubation, the mixture was centrifugated at 5000 rpm for 30 s, followed by washing the resins three times with 1 mL of washing buffer (50 mM Tris-HCl, pH 7.5, 100 mM NaCl, 1% Igepal CA-630, 0.1% SDS, and 0.5% sodium deoxycholate supplemented with Protease Inhibitor Cocktail tablets (Roche, 04693132001). After complete removal of the washing buffer, 20 µL of a 2xSDS-PAGE sample buffer (63 mM Tris, pH 6.8, 25% Glycerol, 2% SDS, 0.01% bromophenol blue, and 5% β-mercaptoethanol) were added to the resins, vortexed briefly, and heated at 95°C for 10 min for elution of the RNA-protein complex. The eluted samples were subjected to Western blotting.

### Isolation of nuclear and cytoplasmic protein

Nuclear and cytoplasmic proteins were isolated using the subcellular protein fractionation kit (ThermoFisher, #78840). In each group, 2 × 10^6^ HEK293T cells were used to isolate the nuclear and cytoplasmic proteins. The volume ratio of CEB: MEB: NEB reagents was 200:200:100 µL, respectively. Finally, samples were separated on 10% SDS-polyacrylamide gels and analyzed by immunoblotting with the indicated antibodies. The α-tubulin (1∶10,000; Sigma-Aldrich, T6074) was used as a marker for cytoplasm, and Lamin B1 (1:1,000; Santa Cruz, sc-374015) was used as a marker for the nucleus.

### Quantitative Reverse Transcription Polymerase Chain Reaction (RT-qPCR)

Total RNA was extracted from cells using Trizol (ThermoFisher, #15596026) according to the manufacturer’s instructions. cDNA was synthesized from total RNA using the cDNA Reverse Transcription kit (ThermoFisher, #4368814). RT-qPCR was performed with a Power SYBR Green PCR Master Mix (Applied Biosystems, #4367659) in a StepOnePlus™ Real-Time PCR System (Applied Biosystems). PCR conditions were 95°C for 2 min, followed by 40 cycles of 95°C for 5 s and 60°C for 25 s. The specificity of each pair of primers was checked by melting curve analysis (95°C for 15 s, 60°C for 1 min, and a continuous rise in temperature to 95°C at 0.3°C/s ramp rate, followed by 95°C for 15 s). To check reproducibility, each assay was performed with technical triplicates for each of the three biological samples. Primers are listed in Table S2.

### RNA Immunoprecipitation (RIP) and m6A RNA Immunoprecipitation (MeRIP) assay

An RIP experiment was performed using the Magna RIP kit (Millipore, #17–700) according to the manufacturer’s instructions. Briefly, HEK293T cells were transiently transfected with Wt or mutant *huORF*^*chop*^-reporter constructs for 24 h, and then 2 × 10^7^ cells were washed with ice-cold PBS and lysed with 100 µL of RIP lysis buffer. Then, the cell lysates were immunoprecipitated with protein A/G magnetic beads conjugated to anti-HnRNPA3 antibody (Proteintech, 25142-1-AP) or normal rabbit IgG (Proteintech, 30000-0-AP) overnight at 4°C. After RNA purification, RT-qPCR was used to measure the levels of *CHOP* 5’UTR in the protein-RNA complexes.

The MeRIP assay was conducted using two MeRIP kits: the EpiQuik CUT&RUN m6A RNA Enrichment (MeRIP) kit (Epigentek, P9018-24) and the Magna RIP kit (Millipore, #17–700). MeRIP assays were performed according to each manufacturer’s protocols. Basically, 20 µg of total RNA were used for the EpiQuik CUT&RUN m6A RNA Enrichment (MeRIP) kit; 1 µg total RNA was used as an internal control. For the Magna RIP kit, 2 × 10^7^ cells and 100 µL of RIP lysis buffer were used in each group, and 5 µg of m6A antibody (Proteintech, 68055-1-Ig) or normal mouse IgG (Millipore, CS200621) were used in each group. Afterwards, the mRNA fragments containing m6A were pulled down by a beads-bound m6A capture antibody. The enriched m6A RNA was then released, purified, eluted from the antibody, reversely transcribed into cDNA, and detected by RT-qPCR. Primers are listed in Table S2.

### Sucrose gradient analysis

The use of sucrose gradient centrifugation to perform polysome profiling was described previously [2], except that cells were incubated with Cycloheximide (100 µg/ml; Sigma-Aldrich-Aldrich, C7698) for 5 min at 37°C, and the fractionated RNA (500 ng) was subjected to RT-qPCR. 500 pg of Firefly luc RNAs were added into each sample for normalization before RT-qPCR. The Firefly luc RNA was in vitro synthesis using T7 Transcription kit (Promega, P1300). Primers are listed in Table S2.

### Immunocytochemical detection

Immunocytochemical detection was performed according to standard protocol with some modifications. Briefly, HEK293T cells were plated on a cell culture slide (SPL, 30102). After transfection or stress treatment, cells were fixed in 4% paraformaldehyde for 10 min at room temperature and permeabilized for 5 min in PBS containing 0.2% Triton X-100. Cells were washed three times with PBSX (0.1% Triton-X100/1xPBS) and then blocked with blocking solution (1% BSA in PBSX) for 1 h at room temperature. Primary antibody was diluted in blocking solution and incubated overnight at 4°C. Cells were washed with PBSX for 3 min and stained with DAPI solution (Sigma) in a concentration of 10 µg/ml for 5 min, and then washed again with PBSX for three times. Finally, the Fluoromount (TM) Aqueous Mounting Medium (Sigma, F4680) was added on the slide, and mounted by cover slips. Images were observed using Confocal Microscopy (Leica Microsystems, Wetzlar, Germany, LSM 780). Antibodies were CoraLite^®^ Plus 488-conjugated HNRNPA3 Polyclonal antibody (Proteintech, CL488-25142; 1:200) and anti-PP11 antibody (Abcam, ab18520; 1:100) conjugated with Alexa Fluor^®^ 647 using Alexa Fluor^®^ 647 Conjugation kit (Abcam, ab269823).

### Prediction of the RNA secondary structure

Secondary structure profiles were generated using RNAstructure, a web server for RNA secondary structure prediction (https://rna.urmc.rochester.edu/RNAstructureWeb/index.html) [27].

### Statistical analysis

All the data are presented as mean ± SD (standard deviation) and described as the mean ± SD in GraphPad Prism 5. The significance of the difference between the two groups was assessed using Student’s t-test. One-way ANOVA with Tukey´s multiple comparisons test was performed for comparison between more than two groups. The values of *P* < 0.001 (***) were shown to represent the significant difference. All experiments were replicated three times.

## Results

### Screening and identification of putative proteins that bind and interact with Endouc/ENDOU-1 in the context of *uORF*^*chop*^-MTI

**Fig. 1:**
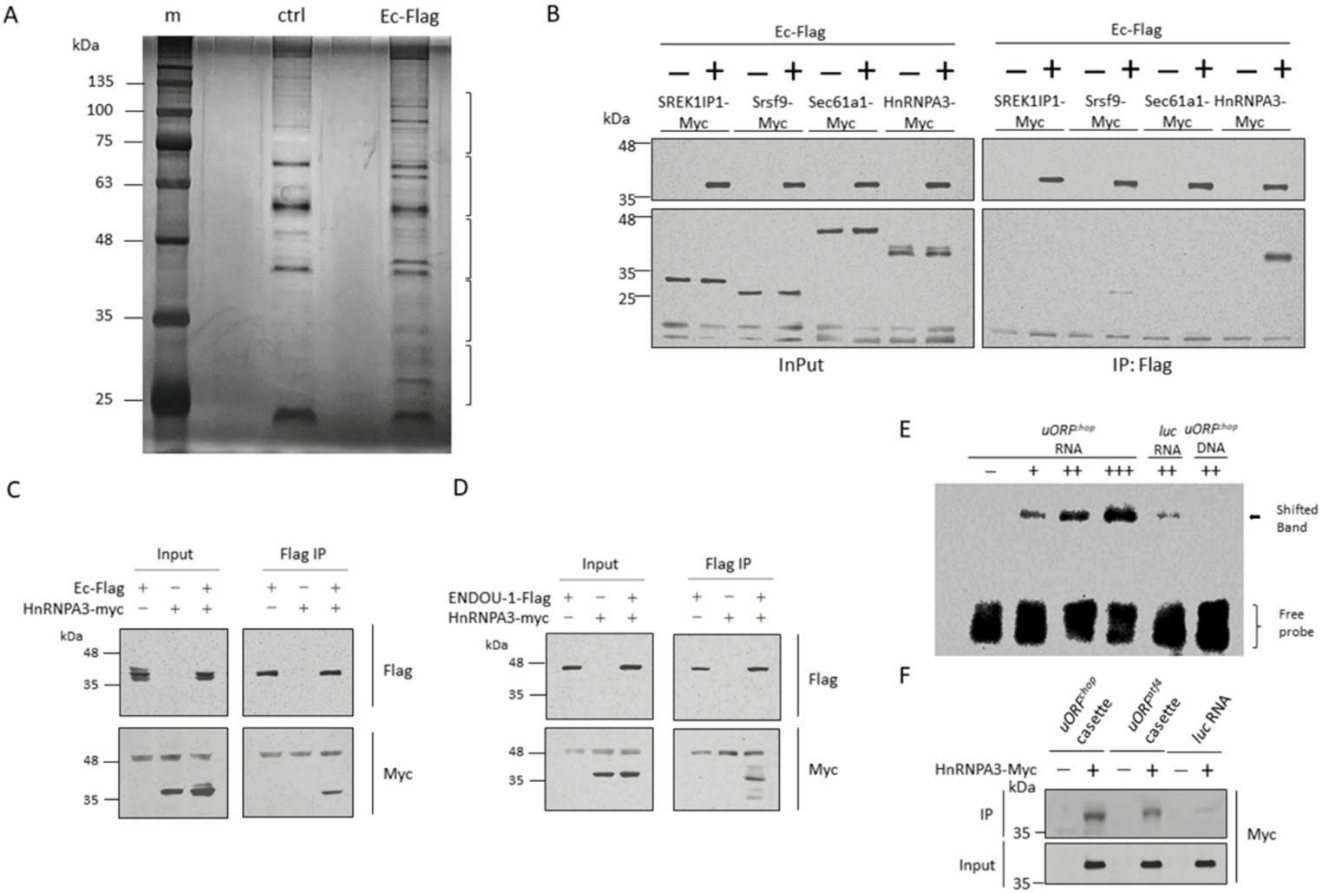
Screening and identification of putative proteins that interact with zebrafish Endouc, human ENDOU-1 and human *CHOP* uORF RNA. **A** The pull-down assay. Total lysates extracted from HEK293T cells were pulled down by recombinant zebrafish Endouc fused with Flag (Ec-Flag) produced from Sf21 insect cells. The precipitated proteins were analyzed on SDS-PAGE by silver staining: lane m, protein markers; lane ctrl, protein profiles of mixture containing Flag beads, extracts of Sf21 wild-type (Wt) cells and total lysates extracted from HEK293T cells served as control; and lane Ec-Flag, protein profiles of mixture containing Flag beads, Ec-Flag produced by Sf21 and total lysates extracted from HEK293T cells. Brackets on the right represented the gel slices excised for LC-MS/MS. **B** Four putative proteins that interacted with zebrafish Endouc-Flag (Ec-Flag) were screened by co-immunoprecipitation (Co-IP). HEK293T cells were transfected with empty pCS2 vector or pCS2 vector to express Flag-tagged zebrafish Endouc (Ec-Flag), or Myc-tagged human HnRNPA3 (HnRNPA3-Myc), human Sec61α1 (Sec61α1-Myc), human Srsf9 (Srsf9-Myc), and human SREK1IP1 (SREK1IP1-Myc). Cell lysates were subjected to IP with anti-Flag-HRP antibody or anti-Myc-HRP antibody. The presence of Flag- and Myc-tagged recombinant proteins in cell extracts prior to IP was controlled using anti-Flag and anti-Myc antibodies (Input). Co-IP of (**C**) zebrafish Endouc (Ec-Flag) or (**D**) human ENDOU-1 (ENDOU-1-Flag) with human HnRNPA3 (HnRNPA3-Myc). Either Myc-tagged human HnRNPA3 (HnRNPA3-Myc), Flag-tagged zebrafish Endouc (Ec-Flag) or human ENDOU-1 (ENDOU-1-Flag) produced by HEK293T cells was subjected to IP. **E** Electrophoretic mobility shifted assay of RNA. Biotin-labeled *huORF*^*chop*^ transcript was reacted with an increased amount of HnRNPA3-Myc. The positions of free RNA (free probe) and RNA–protein complex (shifted band) were indicated on the right. The luc 105‐nt RNA and single‐stranded *huORF*^*chop*^ 105‐nt DNA served as negative controls. **F** Western blot analysis after RNA pull-down assay to investigate the interaction between HnRNPA3 and biotin-labeled RNA, as indicated. HnRNPA3-Flag-expressing HEK293T cells were used to collect the protein lysate for RNA pulldown and analysis by immunoblotting with Flag antibody. HnRNPA3 exhibited more affinity to *huORF*^*chop*^ RNA than that of either *uORF*^*atf4*^ RNA or luc RNA

We immunoprecipitated zebrafish Endouc using LC-MS/MS to analyze its interactome. First, we used recombinant zebrafish Endouc fused with Flag tag (Endouc-Flag) produced by the Baculovirus expression system. Recombinant Endouc-Flag protein was applied in the Flag pull-down assay to extract total proteins from HEK293T cells, followed by silver staining analysis (Fig. 1A). LC-MS/MS was then used to analyze the interacting proteins bound by Enoduc-Flag. Samples were loaded onto polyacrylamide gels and separated by electrophoresis, followed by preparing sliced gel bands for LC-MS/MS. Pull-down without adding Enoduc-Flag was used as the negative control to subtract background signaling. A total of 318 proteins were obtained from LC-MS/MS, followed by gene ontology analysis to classify proteins interacting with Endouc into several functional categories, including RNA binding/processing, translation, chromosome interaction, ER association, and ubiquitination. Out of 55 candidates, four putative RBPs (Table S1) were selected to confirm their interaction with Endouc/ENDOU-1 in vitro, including human SREK1IP1, Srsf9, Sec61α1 and HnRNPA3. Recombinant SREK1IP1, Srsf9, Sec61α1 and HnRNPA3 fused with Myc tag were then produced using HEK293T cells, followed by IP with Endouc-Flag. Results demonstrated that HnRNPA3 and Srsf9, but not SREK1IP1 or Sec61α1, interacted with Endouc (Fig. 1B), suggesting that HnRNPA3 and Srsf9 might bind with Endouc with high affinity. However, since HnRNPA3 showed more affinity for Endouc than Srsf9, we chose HnRNPA3 for further experiments to determine whether it could also interact with mammalian ENDOU-1. Results showed that HnRNPA3 bound both zebrafish Endouc and mammalian ENDOU-1 with equally high affinity (Figs. 1C and D), suggesting that the interaction between HnRNPA3 and Endouc/ENDOU-1 was conserved across species.

### HnRNPA3, a RBP, presented a higher affinity to *huORF*^*chop*^ RNA compared to other RNAs

To confirm binding between HnRNPA3 protein and the *huORF*^*chop*^ transcript, we performed an RNA-EMSA using biotin-labeled, full-length *huORF*^*chop*^ transcript and purified recombinant protein HnRNPA3-Myc. Results showed that HnRNPA3-Myc could directly interact with the *huORF*^*chop*^ transcript in a dose-dependent manner (Fig. 1E). However, a slight shift was detected in the luc mRNA band (Fig. 1E) suggesting that the interaction between HnRNPA3-Myc and *huORF*^*chop*^ transcript was not specific. Next, to examine whether HnRNPA3-Myc could interact with the *Activating transcription factor 4* (*ATF4*) uORF (*uORF*^*atf4*^) transcript, another uORF-tagged transcript, but one involved in Integrated Stress Response (ISR) regulation, we performed an RNA pull-down assay. Results also showed an association between HnRNPA3-Myc and the *uORF*^*atf4*^ transcript (Fig. 1F), but one with notably weaker affinity of HnRNPA3-Myc when compared to its interaction with the *huORF*^*chop*^ transcript. These results confirm that HnRNPA3 directly interacts with the *huORF*^*chop*^ transcript and, as a result, presents higher affinity when compared to that of other ISR regulators, such as the human *uORF*^*atf4*^ transcript.

### Overexpression of HnRNPA3 promoted the translation of the Downstream Coding Sequence (DCS) of *huORF*^*chop*^ transcript

**Fig. 2:**
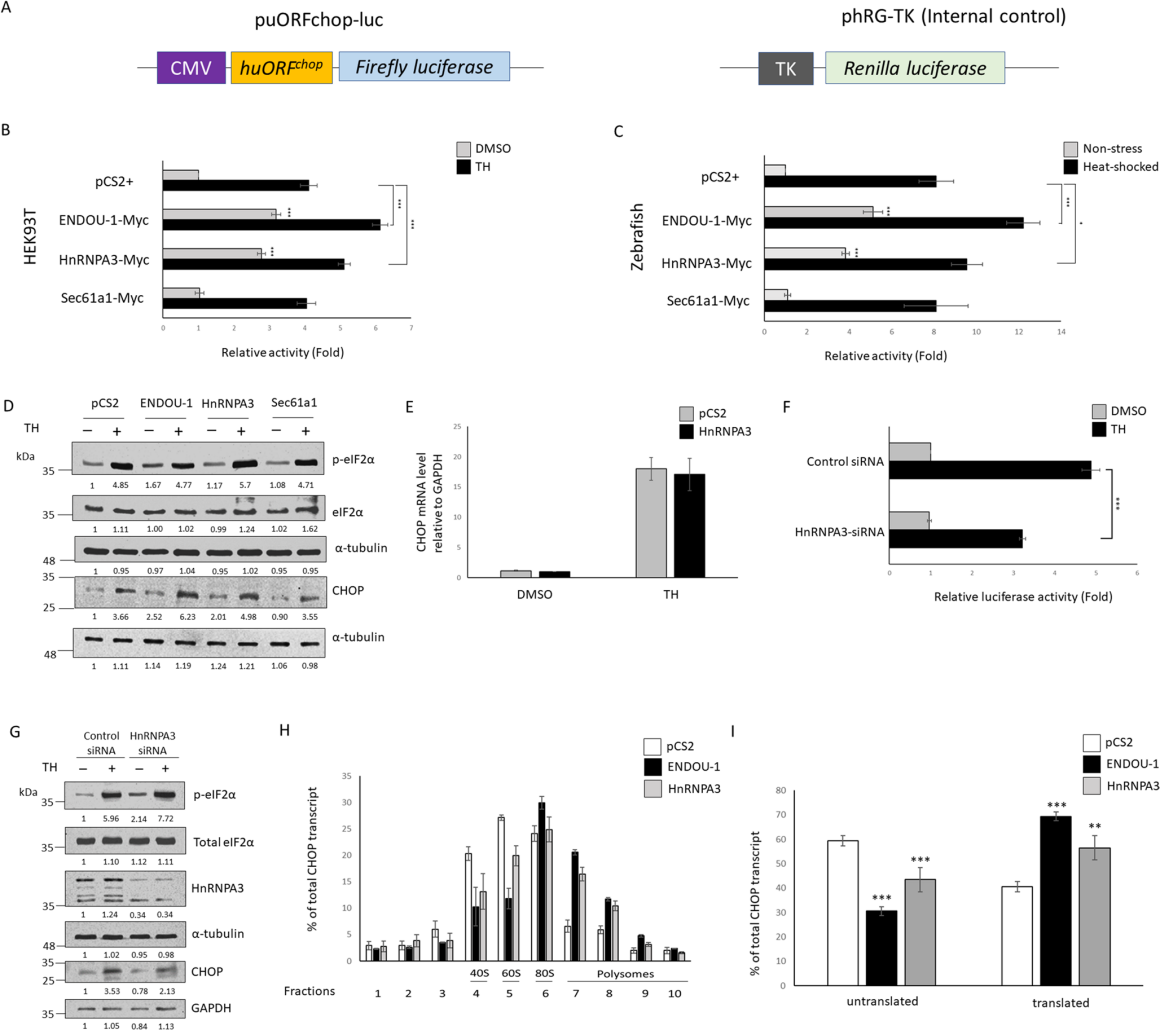
HnRNPA3 is positively correlated with *CHOP* translation. **A** Schematic showing the dual-luc reporter constructs for measuring *huORF*^*chop*^-mediated translational inhibition. phRG-TK was used as an internal control. **B** Dual-luc assay was used to analyze the effect of HnRNPA3 on *huORF*^*chop*^-MTI. Histograms present the luc activity obtained from HEK293T cells co-transfected with puORFchop‐luc, phRG‐TK, and each indicated plasmid and then treated with either DMSO (control group; grey column) or Thapsigargin (TH; stress group; solid column) for 6 h, followed by analysis of luc activity. Cells transfected with pCS2vector and kept at normal conditions served as a control group. Relative luc activity was represented by the fold increase of Fluc/Rluc ratio over that obtained from the control group normalized to 1. Data were averaged from three independent trials and presented as mean ± SEM. ***P ≤ 0.001 (one-way ANOVA, followed by Tukey’s multiple comparison test.) (**C**) Histograms show the luc activity obtained from zebrafish embryos microinjected simultaneously with puORFchop‐luc, phRG‐TK, and each indicated plasmid, followed by analysis of luc activity at 96 h post-fertilization (hpf). Embryos microinjected with the pCS2 vector during normal conditions (grey column) served as a control group, while the microinjected embryos at 72 hpf subjected to 40°C for 1 h comprised the heat‐shocked stress group (solid column). Relative luc activity was determined as above. *P ≤ 0.001; ***P ≤ 0.001 (one-way ANOVA, followed by Tukey’s multiple comparison test.) (**D**) Western blot analysis. The protein levels of p‐eIF2α, total eIF2α, and CHOP were detected in cells overexpressing protein as indicated under either non-stress (TH, -) or stress (TH, +) conditions. The α‐tubulin and GAPDH served as internal controls. **E** Using quantitative RT-qPCR to determine the relative expression level of *CHOP* mRNA in control or HnRNPA3-overexpressing HEK293T cells under either normal (DMSO) or stress (TH) conditions. Data were averaged from three independent trials and presented as mean ± SEM. **F** Dual-luc assay was used to analyze the effect of HnRNPA3-knockdown on *huORF*^*chop*^-MTI under either control (DMSO) or stress (TH) conditions in HEK293T cells. Data were averaged from three independent trials and presented as mean ± SEM. Statistical analysis was performed as above. **G** Western blot analysis. The protein levels of p‐eIF2α, total eIF2α, and CHOP expressed in HnRNPA3-knockdown cells were detected under either non-stress (TH, -) or stress (TH, +) conditions. The α‐tubulin and GAPDH served as internal controls. **H** RT-qPCR analyses of the gradient distribution of three mRNAs after polysome profiling assay (PPA). Lysates from control (pCS2; blank column), ENDOU-1-overexpressing (ENDOU-1; solid column), and HnRNPA3-overexpressing (HnRNPA3; grey column) cells were subjected to PPA. The resultant fractions from 1‐10 collected from a sucrose gradient were subsequently subjected to RT-qPCR assay to quantify *CHOP* transcripts (*CHOP* mRNAs). The distribution showing the relative abundance of *CHOP* transcripts contained in each fraction was determined. **I** Relative abundance (in percentage) of *CHOP* mRNA presented within monosome- and polysome-containing fractions. Fractions labeled as ‘’untranslated’’ contained 40S, 60S ribosomal subunits (fractions 3 ~ 5), while fractions labeled as ‘’translated’’ contained 80 S monosome, light and heavy polysomes (fractions 6–10). Data were averaged from three independent trials and presented as mean ± SEM. ***P ≤ 0.001 (one-way ANOVA, followed by Tukey’s multiple comparison test. Protein levels relative to each internal control (α-tubulin or GAPDH) are presented below each lane

Next, we examined whether interaction between HnRNPA3 and both ENDOU-1 and *huORF*^*chop*^ RNA would influence *huORF*^*chop*^-MTI. To accomplish this, we quantified the increased DCS of luc reporter induced by the overexpression of HnRNPA3 through an in vitro study using HEK293T cells. Under non-stress, luc activity was increased ~ 3.19- and 2.78-fold when cells overexpressed ENDOU-1 and HnRNPA3, respectively (Figs. 2A, B). This increased DCS of luc was also observed in the results from both ENDOU-1- and HnRNPA3-overexpressing cells under TH, a well-used stress inducer, treatment (Fig. 2B).

For in vivo experiments, under non-stress, Wt zebrafish embryos overexpressing ENDOU-1-myc exhibited 5.1-fold higher luc activity than that of the control group, while those overexpressing HnRNPA3-myc exhibited 3.8-fold higher luc activity (Fig. 2C), suggesting that the overexpression either HnRNPA3 or ENDOU-1 could increase the translation of luc, even in non-stress. Meanwhile, compared to the control group, the luc activity under stress was substantially increased in the ENDOU-1-myc- and HnRNPA3-myc-injected embryos compared to the luc activity of ENDOU-1-myc- and HnRNPA3-myc-overexpressing cells under non-stress, respectively (Fig. 2C), which is indicative of *huORF*^*chop*^‐MTI abrogation under stress because the increased fold of luc activity in stressed cells was greater than that in non-stress. However, in a parallel experiment, we found that in vitro and in vivo overexpression of Sec61α1, another candidate obtained from Endouc pull-down assay, didn’t display significant difference on increased folds of reporter activity under non-stress or stress compared to those of the control group, respectively (Fig. 2C).

Therefore, unlike Sec61α1, both in vitro and in vivo results showed that increased folds of luc activity driven by either ENDOU-1 or HnRNPA3 overexpression under either non-stress or stress were significantly higher than those of the control groups, respectively. Taken together, we concluded that the overexpression of HnRNPA3 causes a dramatic increase of reporter expression that allows HnRNPA3 to repress *huORF*^*chop*^-MTI, resulting in the translation of *huORF*^*chop*^–tag transcript in a manner similar to that of ENDOU-1.

The level of endogenous CHOP protein expressed in HnRNPA3-overexpressing cells was also determined. During non-stress, compared to either control cells or Sec61α1-overexpressed cells, HnRNPA3-overexpressed HEK293T cells displayed increased CHOP protein (Fig. 2D), suggesting that overexpression of HnRNPA3 could overcome the inhibitory trap of *huORF*^*chop*^ element in the non-stress condition, resulting in increased *CHOP* translation. Meanwhile, during stress, the level of increased CHOP protein detected in HnRNPA3-overexpressing cells was similar to that observed in ENDOU-1-overexpressing cells (Fig. 2D). Moreover, when we ran RT-qPCR, results revealed that the overexpression of HnRNPA3 did not induce *CHOP* mRNA level during non-stress and stress (Fig. 2E), suggesting that HnRNPA3-mediated increase of *CHOP* expression occurred at the translational, not transcriptional, level. Therefore, the overexpression of HnRNPA3 was found to induce *CHOP* translation during both normal and stress conditions, consistent with results from the in vivo luc reporter assay described above.

As we observed above, it was previously reported that eIF2α phosphorylation (p-elF2α) induces *CHOP* translation by facilitating ribosomal bypass of the inhibitory uORF element [3]. Of note, we previously found that ENDOU-1 overexpression could induce p-elF2α, resulting in *CHOP* translation that reached maximal level [4]. Then, when we analyzed p-eIF2α in HnRNPA3-overexpressing cells, we found that it remained unchanged, similar to that of control cells. Moreover, overexpression of HnRNPA3 did not induce other stress factors expression (Fig. S1). Therefore, although ENDOU-1 could increase p-eIF2α level to, in turn, maximize *CHOP* translation, this line of evidence suggested that HnRNPA3 also facilitated *CHOP* translation and repressed *uORF*^*chop*^-MTI, but in a p-eIF2α-independent manner.

Next, we performed siRNA to knock down HnRNPA3 in HEK293T cells under normal or stress and compared the results to cells transfected with control-siRNA. Results showed that both reporter gene and endogenous CHOP were reduced in HnRNPA3-knockdown cells (Fig. 2F, G), suggesting that the presence of HnRNPA3 was positively correlated with CHOP protein level. Interestingly, while the level of p-eIF2α in HnRNPA3-overexpressing cells remained unchanged, the level of p-eIF2α increased significantly in HnRNPA3-knockdown cells (Fig. 2G), suggesting that cellular HnRNPA3 is required for homeostasis.

To prove that HnRNPA3 plays a critical role in the translation of *CHOP* mRNA, we performed ribosome profiling following HnRNPA3 overexpression (Fig. 2H, I, S2). We measured the level of endogenous *CHOP* transcripts among the sucrose fractions. In the control cells, the *CHOP* mRNAs were largely associated with monosomes (fractions 4 and 5, Fig. 2H, I). However, in either ENDOU-1- or HnRNPA3-overexpressing cells, the *CHOP* mRNAs displayed a shift of 69 and 56% of transcripts from 40 S to 60 S monosomes toward 80 S and light polysomes, respectively (fractions 6–8, Fig. 2H, I). These results suggested that the addition of HnRNPA3 caused a higher percentage of *CHOP* mRNAs to shift from the monosome toward the light polysome fraction to proceed with continuous translation, proving that HnRNPA3 functions as a positive effector for *CHOP* translation.

### Functional link between ENDOU-1 and HnRNPA3 based on RNA processing and regulation

**Fig. 3:**
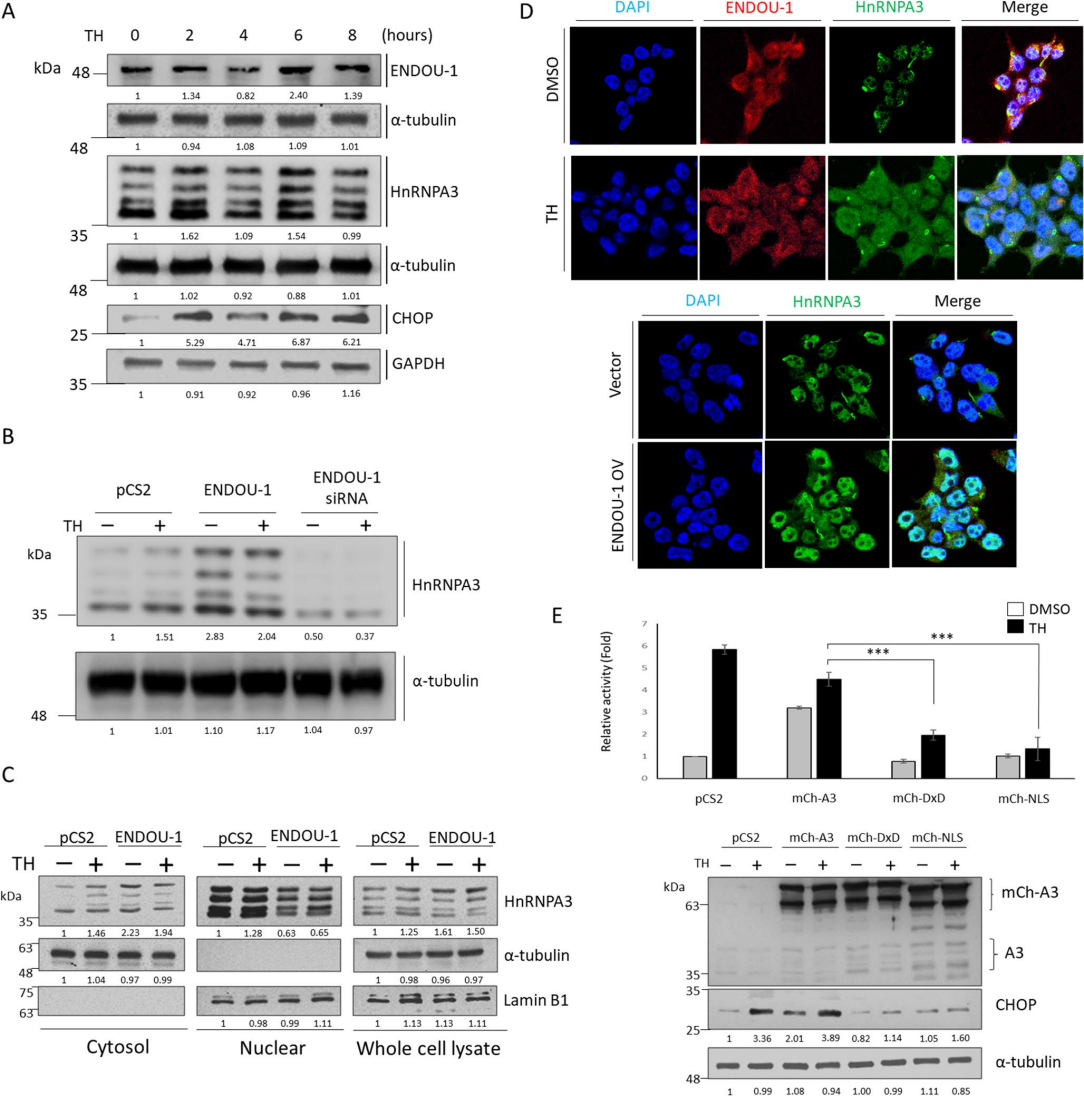
ENDOU-1 triggers the cytoplasmic translocation of HnRNPA3 to increase *CHOP* translation during ER stress. **A **Western blot analysis of the time course of expression levels of human ENDOU-1, HnRNPA3, and CHOP proteins in HEK293T cells after Thapsigargin (TH; stress inducer) treatment from 0〜8 h. The α‐tubulin and GAPDH served as internal controls. **B** Western blot analysis of HnRNPA3 in ENDOU-1-overexpressing cells or ENDOU-1-knockdown cells. The α‐tubulin served as an internal control. **C** Immunoblot of HnRNPA3 expression in cytoplasmic and nuclear fractions of HEK293T- or ENDOU-1-overexpressing cells. Lamin B1 and α-tubulin served as loading controls for nuclear and cytoplasmic fractions, respectively. Total protein lysates were used as an Input for the loading control. **D** Immunofluorescence staining of ENDOU-1 A3 (red), HnRNPA3 (green), and DAPI (blue) was obtained to observe the localization of HnRNPA3 and ENDOU-1 in HEK29ET cells under normal (DMSO) and stress conditions (TH). **E** Immunocytochemical detection of HnRNPA3 (green) and DAPI (blue) in HEK293T transfected with empty vector or pENDOU-1-flag. **F **Up: Dual-luc assay was used to analyze the effect of HnRNPA3 and its derivatives on *huORF*^*chop*^-MTI under either control (DMSO) or stress (TH) conditions in HEK293T cells. Histograms presented the luc activity obtained from HEK293T cells, which were co‐transfected simultaneously with puORFchop–luc, phRG‐TK, and each of the plasmids, as indicated, for 24 h and treated with DMSO or TH for 6 h. Relative luc activity was represented by the fold increase of Fluc/Rluc ratio over that obtained from the control group (DMSO, pCS2) normalized to 1. Data are averaged from three independent experiments and presented as mean ± SEM. ***P ≤ 0.001 (one-way ANOVA, followed by Tukey’s multiple comparison test. Down: Western blot analysis. Cells in the remaining half were used to analyze the protein levels of mch-A3 derivative and CHOP, as indicated. The a-tubulin served as an internal control. Protein levels relative to each internal control (α-tubulin or GAPDH) are presented below each lane

Nonetheless, studies have not uniformly agreed on the state of HnRNPA3 expression during stress conditions since some reports have shown down regulation [28], while others have not [29]. Therefore, to sort out the lack of consensus in the context of *CHOP* translation, a time course experiment was used to compare the expression of HnRNPA3, ENDOU-1, and CHOP proteins during ER stress. Results showed that (1) the expression of HnRNPA3 was positively corelated with those of ENDOU-1 and CHOP, (2) the dynamically fluctuant expression patterns of these three proteins were correspondently consistent, and (3) they all displayed a relatively higher level after 6 h TH treatment (Fig. 3A). Moreover, ENDOU-1-overexpression induced HnRNPA3 expression, while ENDOU-1-knockdown led to reduce HnRNPA3 expression (Fig. 3B), suggesting that HnRNPA3 is induced by ENDOU-1.

We next undertook a mechanistic study to understand the functional link between ENDOU-1 and HnRNPA3It has been reported that nuclear localization of HnRNPA3 is important for G4C2 repeat RNA degradation [19]. It has also been reported that the localization of HnRNPA3 can shift from the nucleus to the cytoplasm (nucleus/cytoplasm) in virus-infected cells [30]. In this study, we investigated whether the overexpression of ENDOU-1 might influence the nucleus/cytoplasm distribution of HnRNPA3 in cells. When nuclear and cytoplasmic extracts were isolated, we detected the respective protein level of HnRNPA3. Results showed the increase of cytoplasmic HnRNPA3 in cells treated with TH, compared to that of control (DMSO) group (Fig. 3C). However, in ENDOU-1-overexpressed cells treated either with DMSO or TH, we found that the expression of HnRNPA3 was increased significantly in the cytoplasm, but decreased in the nucleus (Fig. 3C). Moreover, as shown on the fact that the distribution of HnRNPA3 was increased in cytoplasm but decreased in nuclear were also observed in HepG2 (Fig. S3A) and HeLa cells (Fig. S3B). These evidences suggested that overexpression of ENDOU-1 can induce an increase of HnRNPA3 expression and also cause the shift of HnRNPA3 from the nucleus to the cytoplasm, providing support for the functional link between ENDOU-1 and HnRNPA3 in cells.

Next, we utilized immunofluorescence to detect the endogenous expression of ENDOU-1 and HnRNPA3. In DMSO-treated cells, ENDOU-1 was expressed in both the nucleus and the cytoplasm, whereas HnRNPA3 was predominantly distributed in the nucleus and slightly distributed in the cytoplasm. However, compared to DMSO-treated cells, cytoplasmic HnRNPA3 was increased significantly in TH-treated cells (Fig. 3D). Moreover, when ENDOU-1 was ectopically expressed, cytoplasmic HnRNPA3 was increased significantly (Fig. 3D). This result is consistent with our nucleus/cytoplasm fraction experiments.

To further investigate whether cytoplasmic HnRNPA3 is involved in *CHOP* translation in the context of stress responses, we performed experiments to study the effects of Wt HnRNPA3 and its mutants [21] on *CHOP* translation. Results showed that ectopic expression of Wt HnRNPA3 (mCh-A3) could disrupt *huORF*^*chop*^-MTI, allowing reporter translation (Fig. 3E). However, ectopic expression of mutant HnRNPA3 DxD (mCh‐A3DxD), a mutant variant that cannot bind RNA [31], failed to induce reporter and CHOP expression (Fig. 3E), suggesting that RNA binding of HnRNPA3 is essential for reporter translation. In contrast, when a strong NLS from simian virus 40 (SV40) large tumor antigen was inserted into the mCh-A3ΔM9 mutant (mCh‐A3NLS), reporter and CHOP expression was noticeably repressed (Fig. 3E), suggesting that cytoplasmic HnRNPA3 is necessary and sufficient for the disruption of *uORF*^*chop*^-MTI to, in turn, translate the DCS. It can also be deduced from these results that the ectopic expression of ENDOU-1 favors cytoplasmic translocation of HnRNPA3, leading to the disruption of *uORF*^*chop*^-MTI and, hence, the induction of *CHOP* translation under stress conditions. These results confirm that the functional link between ENDOU-1 and HnRNPA3 hinges on the ability of ENDOU-1 to upregulate the cytoplasmic translocation of HnRNPA3 to, in turn, facilitate the maximal translation of *CHOP* during ER stress.

### The reader ability of N6-methyladenosine site (m6A) on the 5’UTR of *CHOP* transcript could affect HnRNPA3-induced *CHOP* translation

**Fig. 4:**
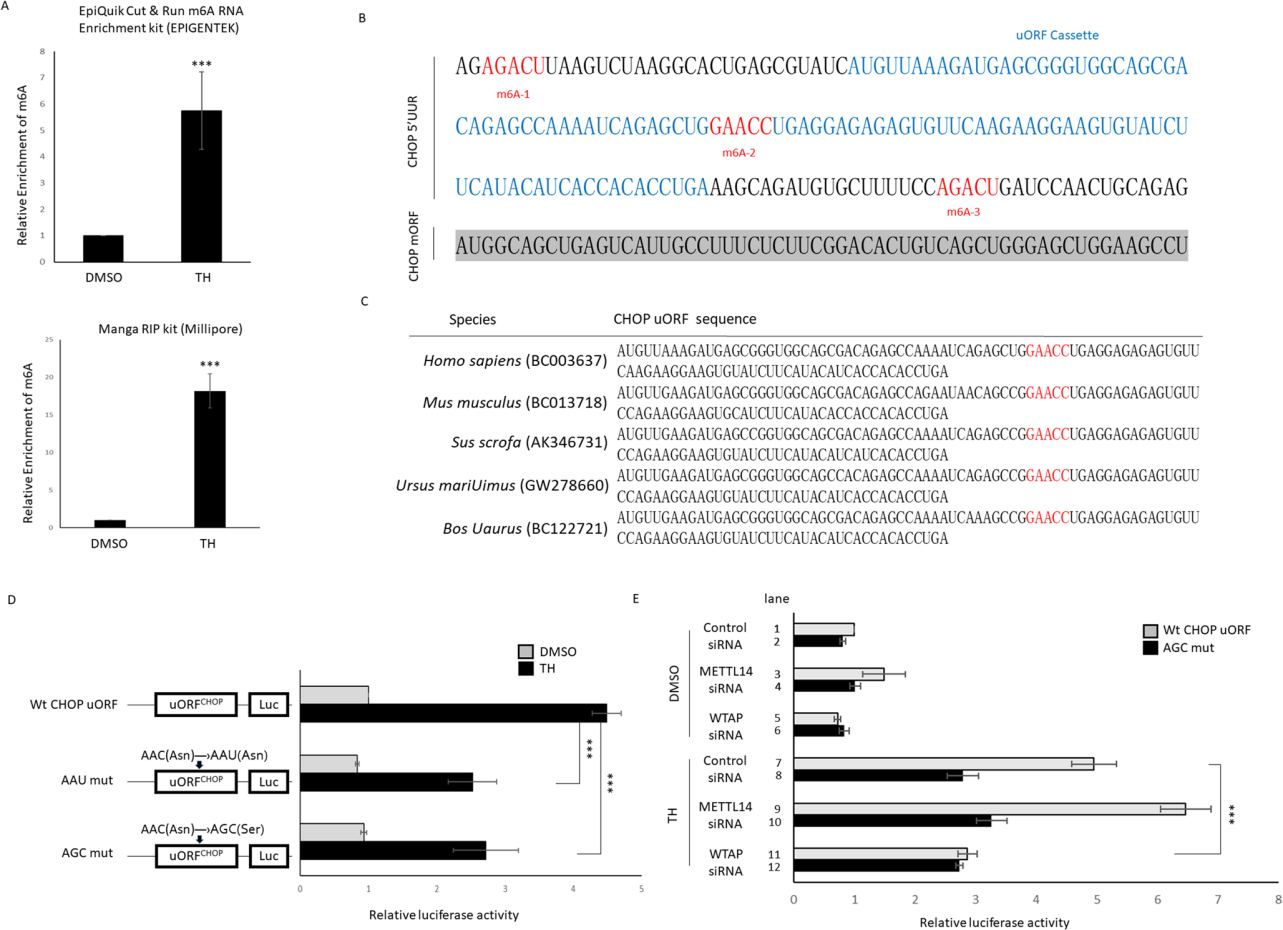
The m6A site on *huORF*^*chop*^ cassette methylated by WTAP can enhance *CHOP* translation during either non-stress or stress. **A** Using MeRIP-qPCR to analyze the m6A levels of *huORF*^*chop*^ from HEK293T cells treated with either DMSO (non-stress) or Thapsigargin (TH; stress). Two MeRIP kits, as indicated upper and lower, were performed, yielding similar results. Data are averaged from three independent experiments and presented as mean ± SEM. ***P ≤ 0.001 (student t-test). **B** Using bioinformatic to predict the potential m6A methylation sites at the 5’UTR and uORF of human *CHOP* transcript were labeled in red and blue, respectively, while the mORF of *CHOP* coding sequence was labeled in gray. **C** Sequence alignment of the uORF of *CHOP* from different vertebrates. Residues conserved between the predicted m6A site in uORF are highlighted in red. **D** Schematic representation of wild-type (Wt) and m6A-2 mutated constructs. Luc activity of HEK293T cells transfected with the indicated plasmid under normal (DMSO) or stress (TH) conditions. Relative luc activity was represented by the fold increase of Fluc/Rluc ratio over that obtained from Wt *CHOP* uORF construct transfected control group normalized as 1. **E** Luc activity of HEK293T cells co-transfected with the indicated plasmid and siRNA under normal (DMSO) or stress (TH) conditions. Relative luc activity was represented by the fold increase of the Fluc/Rluc ratio over that obtained from the co-transfected Wt *CHOP* uORF construct and the control siRNA group normalized as 1. Data presented in (**D**, **E**) were averaged from three independent experiments and are represented as mean ± SD. ***P ≤ 0.001 (one-way ANOVA, followed by Tukey’s multiple comparison test

Recent studies have shown that HnRNPA3 acts as an m6A reader protein in regulating the alternative splicing of the chromosomal translocation t(8;21) and the resulting oncofusion (AML-ETO) pre-mRNA [32]. Therefore, we hypothesized that the reader ability of adenine methylation on the 5’UTR of *CHOP* RNA could affect HnRNPA3-induced *CHOP* translation. To make such a determination, we first performed m6A-IP-RT-qPCR to quantify the relative m6A level of *CHOP* mRNA. Two different methylation RNA IP (MeRIP) assay kits were used. The results showed that *CHOP* 5’UTR m6A modification was dramatically increased in cells treated with TH, suggesting that m6A modification on the 5’UTR of *CHOP* transcript does occur during ER stress (Fig. 4A, B). Next, we predicted the m6A-binding sites of 5’UTR *CHOP* RNA using RMBase v3.0 for RNA modifications and SRAMP, an online m6A site predictor. Three potential m6A modification sites located at the 5’UTR of *CHOP* RNA were shown (Fig. 4B).

One of these three m6A sites located at the second position (m6A-2) was within the uORF segment of *CHOP* RNA and conserved among many vertebrates (Fig. 4C). We then proposed that this m6A-2 site might play role in regulation of *huORF*^*chop*^-MTI. To prove this hypothesis, we constructed a dual-luc expression system containing either Wt m6A-2, or mutated versions, and examined the reporter activity under normal and stress conditions. The *huORF*^*chop*^-MTI had codon-associated effect because Jousse et al. [1] performed a frameshift mutation within the mRNA that generates a mutated sequence of the *CHOP* uORF-encoded peptide without altering its original nucleotide length. They found that the frameshift mutant of uORF failed to repress downstream reporter expression. Young et al. [33] found that an RNA sequence encoding Ile-Phe-Ile tripeptides within the *CHOP* uORF could stall elongating ribosomes. These evidences suggest that RNA sequence and its encoded amino acid sequence are critical factors involved in *huORF*^*chop*^-MTI. Thus, in our mutant version of the *huORF*^*chop*^ transcript, we took into account both RNA sequence and encoded amino acid residues to preserve the original transcript’s regulatory mechanism, exclusive of the m6A site, and thus avoid changing reporter activity.

To mutate the m6A site within the *huORF*^*chop*^ transcript, two designs were performed. First, we replaced GAACC (17th Asn) with GAAUC (17th Asn) to generate an AAU mutant. The mRNA sequence of AAU mutant was changed, but not its encoded amino acid residues. Second, we replaced GAACC (17th Asn) by GAGCC (17th Ser) to generate an AGC mutant. The methylated Adenosine (A) residue in the AGC mutant was directly mutated, and its encoded amino acid was also changed to a Serine residue at the 17th. These two mutants were inserted individually into the Fluc reporter to prevent the GAACC site from being modified as m6A. This dual-luc assay showed that both AAU and AGC mutants exhibited lower Fluc levels in response to stress treatment compared to the Wt control (Fig. 4D), suggesting that the m6A modification site at m6A-2 located at the 5’UTR of *CHOP* RNA contributed to uORF-mediated reporter translation and that this role was independent of possible codon-associated effects.

### The m6A-2 site on *huORF*^*chop*^ transcript is essential for translation of DCS reporter

It has been reported that Methyltransferase-like 14 (METTL14) promotes *CHOP* mRNA decay through recognizing the m6A site at its 3’UTR [34]. However, we found that the METTL14-knockdown group exhibited reporter activity similar to that of the control group during stress condition (lanes 7 vs. 9), suggesting that METTL14 is not involved in recognizing the m6A-2 at 5’UTR *huORF*^*chop*^ (Fig. 4E). Moreover, Wei et al. [34] showed an increased expression of CHOP protein in METTL14-knockdout cells. Chelmicki et al. [35] also found that METTL14-knockout reduces Methyltransferase-like 3 (METTL3), but increases Wilms’ tumor 1-associating protein (WTAP) expression. Thus, it is possible that increased CHOP in METTL14-knockdout might result from WTAP overexpression. In this study, when WTAP was knocked down in HEK293T cells, the reporter activity was significantly reduced compared to that of the control group (Fig. 4E, lane 7 vs. 11), suggesting that WTAP, which regulates gene expression modifying *huORF*^*chop*^ transcript at m6A, is involved in *CHOP* translation as an m6A writer. Moreover, in AGC mutant reporter, knockdown of METTL14 or WTAP showed similar activity as that in control METTL14- or WTAP-knockdown group during either non-stress (Fig. 4E, lane 2 vs. lanes 4 and 6) or stress (Fig. 4E, lane 8 vs. lanes 10 and 12). Taken together, we conclude that the m6A-2 site methylated by WTAP on the *huORF*^*chop*^ transcript is required to promote the translation of DCS reporter in a codon-independent manner.

### HNRNPA3 regulates *huORF*^*chop*^-MTI in an m6A-dependent manner

**Fig. 5:**
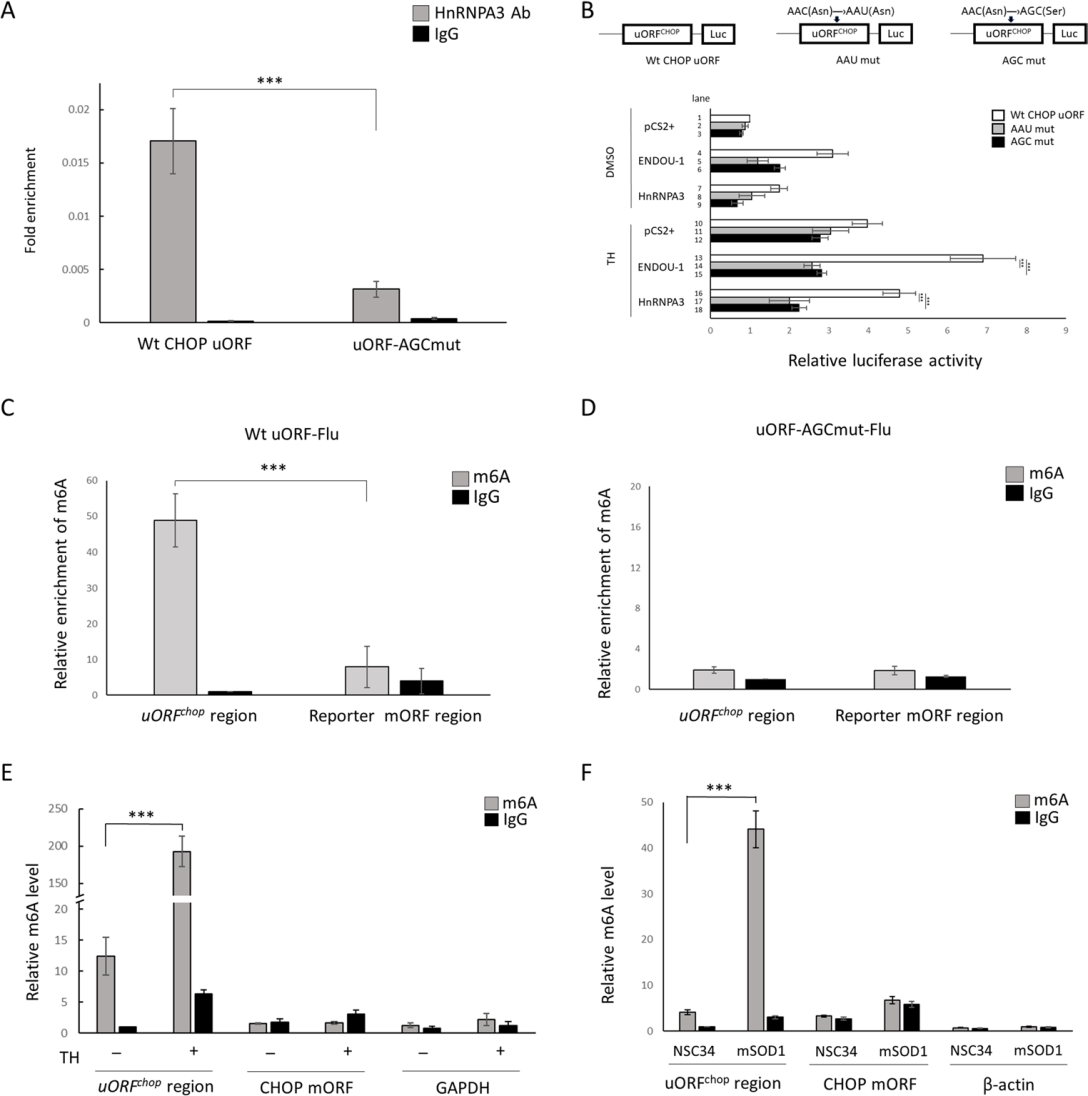
The m6A methylation on *huORF*^*chop*^ recognized by HnRNPA3 is necessary for allowing ENDOU-1 to achieve maximal translation of CHOP protein. **A** RIP-qPCR analysis showed the binding affinity of HnRNPA3 with wild-type (Wt) and mutant (AGC-mut) *huORF*^*chop*^ transcripts in HEK293T cells. IgG served as a negative control. Data were averaged from three independent experiments and presented as mean ± SEM. ***P ≤ 0.001 (one-way ANOVA, followed by Tukey’s multiple comparison test). **B** Schematic representation of Wt and two m6A-2 mutated (AAU and AGC) constructs. The luc activity of HEK293T cells transfected with the indicated plasmid, under normal (DMSO) or stress (TH) conditions. The relative luc activity was represented by the fold increase of Fluc/Rluc ratio over that obtained from pCS2  transfected control group normalized as 1. Data were averaged from three independent experiments and presented as mean ± SEM. *P ≤ 0.05; ***P ≤ 0.001 (one-way ANOVA, followed by Tukey’s multiple comparison test). **C**, **D** HEK293T cells were separately transfected with Wt uORF-luc (**C**) and AGC mutant-luc (**D**) plasmids for 48 h, and then the m6A level of *huORF*^*chop*^ and reporter DCS were evaluated using MeRIP-qPCR. Data presented in (**C**, **D**) were averaged from three independent experiments and are presented as mean ± SEM. ***P ≤ 0.001 (one-way ANOVA, followed by Tukey’s multiple comparison test). **E** HepG2 cells were treated with DMSO or TH for 6 h, and then evaluated the *huORF*^*chop*^ m6A level was evaluated using MeRIP-qPCR. The GAPDH gene was used as a control group. **F** NSC34 and NSC34-SOD1G93A (mSOD1) cells were used to evaluate *uORF*^*chop*^ m6A level using MeRIP-qPCR. The β-actin gene was used as a control. Data shown in (**E** and **F**) were averaged from three independent experiments and presented as mean ± SEM. ***P ≤ 0.001 (one-way ANOVA, followed by Tukey’s multiple comparison test)

RIP experiments were performed and revealed that HNRNPA3 directly bound at m6A-2 (Fig. 5A). If m6A sequence was mutated to AGC, the interaction between HNRNPA3 and m6A-2 was significantly weakened, suggesting that the m6A-2 of *huORF*^*chop*^ is important for HnRNPA3 binding (Fig. 5A) The dual-luc reporter assay further identified that the m6A-2 site was necessary for the disruption of *huORF*^*chop*^-mediated translational inhibition driven by either HnRNPA3 or ENDOU-1.

In ENDOU-1-overexpressed cells, both AAU and AGC mutant reporter activity were reduced significantly compared to Wt control during either non-stress (Fig. 5B, lane 4 vs. lanes 5 and 6) or stress (Fig. 5B, lane 13 vs. lanes 14 and 15). A similar effect was observed in HnRNPA3-overexpressed cells in which both AAU and AGC mutant reporter activity were reduced significantly compared to Wt control during either non-stress (Fig. 5B, lane 7 vs. lanes 8 and 9) or stress (Fig. 5B, lane 16 vs. lanes 17 and 18). MeRIP-qPCR further demonstrated that the m6A level of *huORF*^*chop*^ was significantly decreased when m6A-2 was mutated, whereas the m6A level at the *huORF*^*chop*^ downstream CDS appeared unchanged. (Figs. 5C, D). This result demonstrates a specific regulatory role of the m6A-2 site of *huORF*^*chop*^ transcript, which, when recognized and bound by HnRNPA3, results in the promotion of *CHOP* translation during ER stress through its cooperation with ENDOU-1.

The other two predicted m6A sites located in *CHOP* 5’UTR but outside of *huORF*^*chop*^ were also analyzed (Fig. S4). Irrespective of normal or stress conditions or administration of cells with overexpressed ENDOU-1, HnRNPA3, or even WTAP, the reporter activity driven by *huORF*^*chop*^ containing a mutation at the m6A-1 and m6A-3 site remained unchanged compared to that of Wt *huORF*^*chop*^ (Fig. S4). Moreover, based on secondary structure prediction, we found that the *huORF*^*chop*^ secondary structure was not altered by m6A-2 mutation, suggesting that the reduced reporter activity in m6A-2 mutant results from the failure of methylation, not change RNA structure (Fig S5).

This line of evidence suggested that ENDOU-1 upregulates the cytoplasmic translocation of HnRNPA3 which, in turn, allows it, as an m6A reader protein, to directly bind the m6A-2 methylation site on the *huORF*^*chop*^ transcript and thus act as a positive effector to increase translation of the DCS of mORF in the *huORF*^*chop*^ transcript and effectively abrogate *uORF*^*chop*^-MTI, reaching maximal translation of CHOP protein during ER stress.

### Increased CHOP protein is correspondent with increased m6A on *uORF*^*chop*^

The HepG2 cells are commonly used to study ER stress [36]. Here, we showed that the protein level of CHOP was increased in HepG2 cells under stress (Fig. S6A). Meanwhile, MeRIP-qPCR revealed that the m6A level of *huORF*^*chop*^ was greatly increased in stressed HepG2 cells compared to normal conditions (Fig. 5E). It was noticeable that the *huORF*^*chop*^ cassette was methylated during ER stress, but not the DCS of *CHOP* mORF cassette (Fig. 5E), suggesting that the m6A methylation on *huORF*^*chop*^ is critical for *CHOP* translation during ER stress.

We also employed superoxide dismutase 1 (SOD1) G93A mutant NSC34 cells (mSOD1), which are thought to be responsible for ER stress in ALS neurodegenerative disease [37–39]. We found that, compared to Wt NSC34 cells, the protein level of CHOP in mSOD1 cells was elevated (Fig. S6B). Meanwhile, MeRIP-qPCR revealed that the m6A level of *uORF*^*chop*^ in mSOD1 cells was much higher than that of Wt NSC34 cells (Fig. 5F). Taken together, in our mSOD1 ALS cell model, methylation on *uORF*^*chop*^ is highly expressed when *CHOP* is increasingly translated.

## Discussion

The m6A is a common mRNA post-transcriptional modification, in eukaryotes, playing a pivotal role in such biological processes as mRNA translation, decay, alternative splicing, and nuclear export [40–42]. Although transcriptome-wide m6A mapping indicates an asymmetric distribution of m6A methylation sites enriched near the stop codon [43, 44], they also occur in the coding region and 5’ UTR [45, 46]. Moreover, 5’UTR m6A can promote cap-independent translation under stresses [45]. Recent studies have focused on the role of methylation modification of stress-related mRNA. For example, Zhou et al. [46] identified a methylation site located in ATF4 uORF2 and proved that the m6A of this *uORF2*^*atf4*^ is important during amino acid starvation. Here, we found another example showing that the methylation at 5’UTR of *huORF*^*chop*^ transcript is necessary for *CHOP* translation to reach maximal level during ER stress.

Although uORF-mediated translational control and RNA methylation play important roles in fine-tuning gene expression, little is known about the proteins involved in both roles that act as mediators of mRNA translation. In this study, we revealed that HnRNPA3, an m6A reader protein, cooperates with a RNA endoribonuclease, ENDOU-1, to mediate human *CHOP* translation through repressing the inhibitory *huORF*^*chop*^ cassette. We also found that (1) ENDOU-1 can induce HnRNPA3 to shift from nucleus to cytoplasm; and (2) *uORF*^*chop*^ could be methylated by WTAP and recognized by cytoplasmic HnRNPA3. As suggested by this line of evidence, by inducing cytoplasmic HnRNPA3 to directly bind the m6A-2 methylation site on the *uORF*^*chop*^ transcript, ENDOU-1 is able to repress *uORF*^*chop*^-MTI which results in maximal translation of the DCS of mORF within the human *CHOP* transcript.

**Fig. 6:**
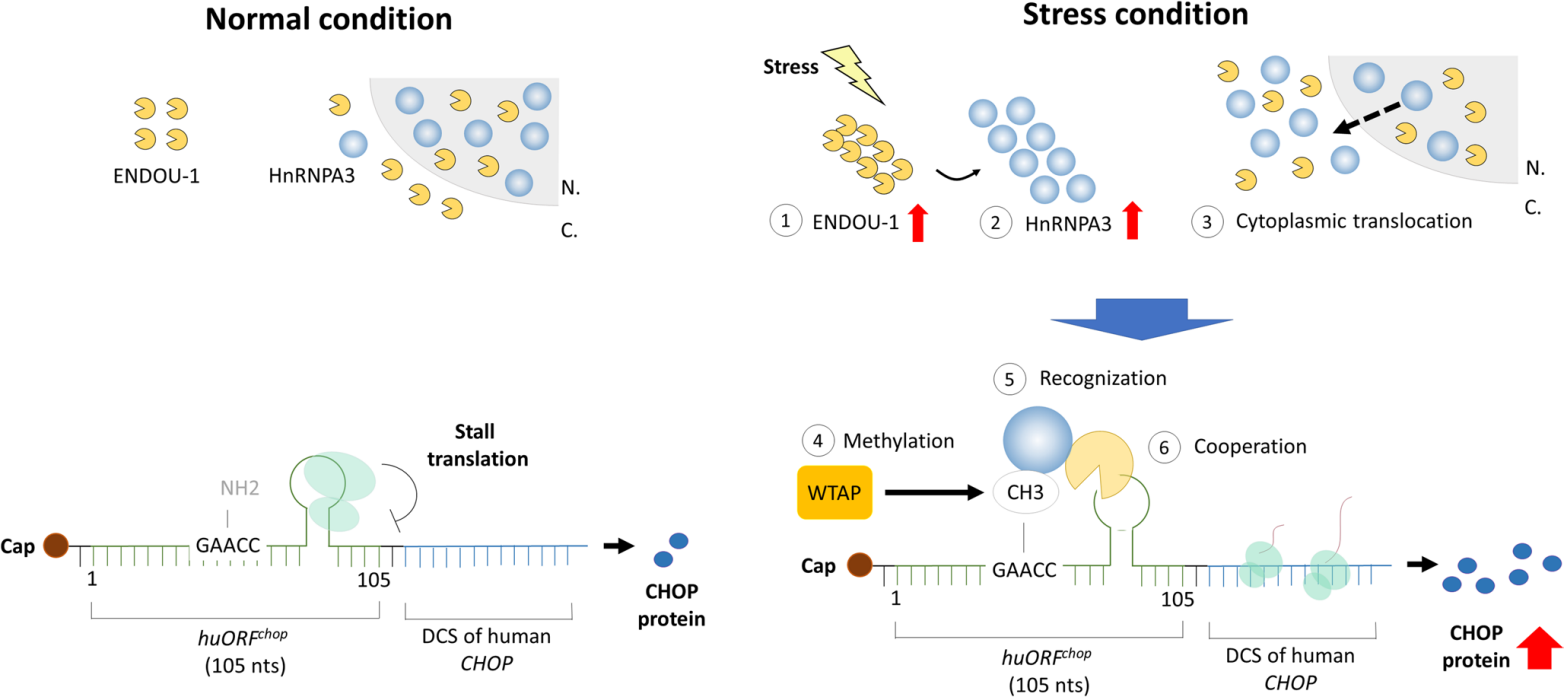
Schematic shows how HnRNPA3 recognizes the m6A site within *uORF*^*chop*^ and acts as a positive modulator of human *CHOP* mRNA translation. In the absence of stress, the methylation of *uORF*^*chop*^ transcript is relatively low, and the translation of CHOP protein is blocked by *uORF*^*chop*^-MTI. In the presence of stress, (1) ENDOU-1 is increased, (2) Increased ENDOU-1 induces the highly expressed HnRNPA3, (3) Increased ENDOU-1 also induces the shift of HnRNPA3 from nucleus to cytoplasm, (4) WTAP methylates N6-adenosine site on the *uORF*^*chop*^ transcript; (5) cytoplasmic HnRNPA3 recognizes and binds to an existing m6A methylation site on the *uORF*^*chop*^ transcript, and, finally, (6) ENDOU-1 promotes *CHOP* translation by cooperating with HnRNPA3 in an m6A-dependent manner, thereby overcoming *uORF*^*chop*^‐MTI. N.: Nucleus; C.: Cytoplasm

Park et al. [47] found that an endonuclease cooperated with m6A reader protein and selectively recognized different m6A sites, cleaved mRNA, and facilitated m6A mRNA decay. Here, we found another example showing that ENDOU-1, an endonuclease, cooperates with HnRNPA3, an m6A reader, to promote DCS translation. Moreover, our luc assay indicates that the m6A-2 at *uORF*^*chop*^ cassette is necessary and sufficient for ENDOU-1 to increase *CHOP* translation to its maximal level during ER stress (Fig. 5, and 6). Thus, we propose that the m6A-2 methylation site of *uORF*^*chop*^ is not only important for HnRNPA3 recognition, but permissive for ENDOU-1 to play its repressive role in *CHOP* translation inhibition. The methylation of RNA may affect RNA folding, and m6A-mediated RNA structural changes may create new protein binding sites or new epitopes for protein interaction [48]. The detailed molecular mechanism that explains how *uORF*^*chop*^ m6A-2 and HnRNPA3 cooperate with ENDOU-1 to facilitate *CHOP* translation during ER stress is worth further investigation. Moreover, since the m6A in 5’UTR can promote cap-independent translation [45], it is also worth investigating whether *uORF*^*chop*^ m6A-2 and HnRNPA3 play a role on cap-independent translation after ENDOU-1 cleaves the 105-nt *huORF*^*chop*^ transcript at position 80G-81U reported by Lee et al. [4].

Finally, both ENDOU-1 and HnRNPA3 were reported to process small nucleolar RNA (snoRNA) known to guide the chemical modification of ribosomal RNAs [49–51], suggesting that ENDOU-1 and HnRNPA3 may also cooperate in snoRNA biosynthesis. The presence and function of m6A in spliceosomal RNAs (snRNAs), such as the spliceosomal U6 snRNA, could also reveal that m6A could serve as a regulator of the post-transcriptional modification of a subset of mRNAs [52]. Therefore, we cannot rule out the possibility that ENDOU-1 and HnRNPA3 might cooperate in recognizing the m6A site and the cleaving of snRNA, thereby contributing to *CHOP* translation through yet another unknown mechanism during ER stress.

## Supplementary Information

Supplementary Material 1.

Supplementary Material 2.

Supplementary Material 3.

Supplementary Material 4.

Supplementary Material 5.

Supplementary Table S1

Supplementary Table S2

## Data Availability

All datasets are provided within the main text or Supplementary Materials and can be obtained from the corresponding authors upon request. This study includes no data deposited in external repositories.
