# Supplementary material for: ENDOU-1-induced cytoplasmic HnRNPA3 recognizes m6A methylation on the upstream reading frame of human CHOP transcripts to achieve maximal CHOP translation
Source: Cell Mol Life Sci. 2026 Mar 28;83(1):194. doi: 10.1007/s00018-026-06180-7 (PMC13049129; doi:10.1007/s00018-026-06180-7)
Supplement: Supplementary file 1 — Supplementary Material 1. [file 18_2026_6180_MOESM1_ESM.docx]

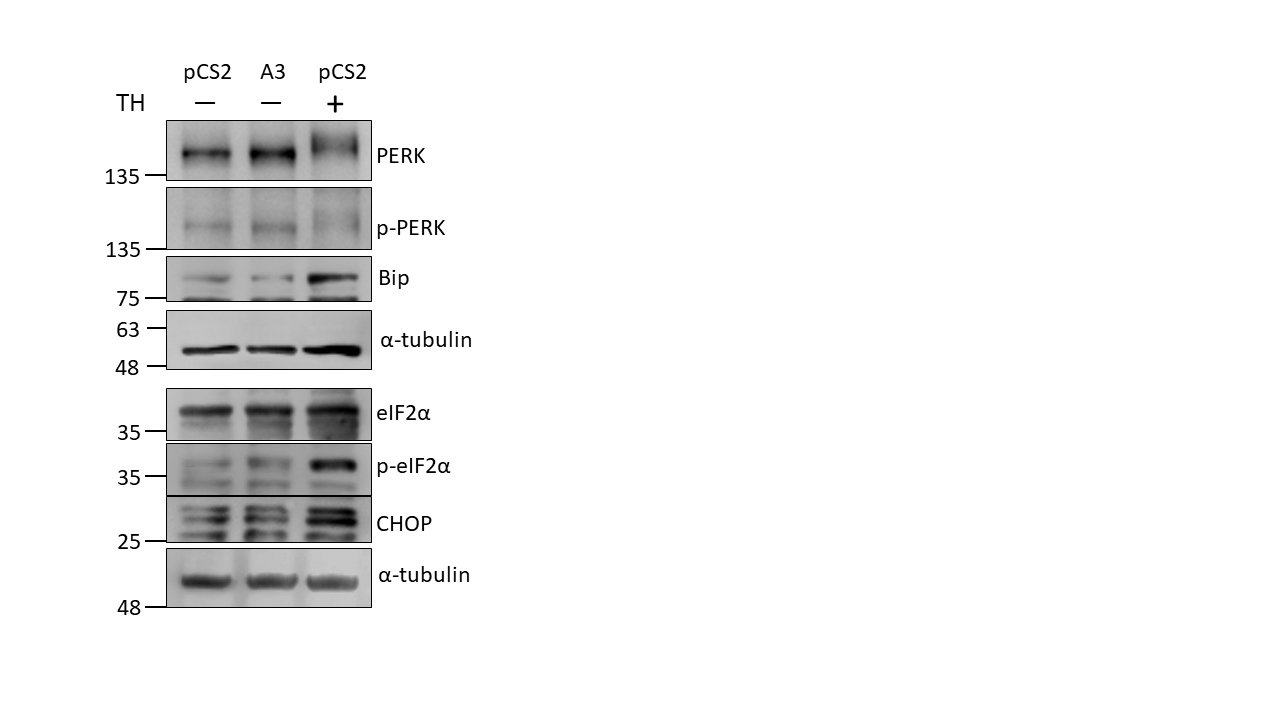


**Figure S1. Overexpression of HnRNPA3 increases *CHOP* translation.**

Western blot analysis of proteins, as indicated, expressed in HEK293T cells overexpressing an empty vector (pCS2) or a vector containing HnRNPA3 cDNA (A3). Cells were treated without Thapsigargin (TH; -) or with TH (+) served as a positive control. The α‐tubulin served as an internal control.


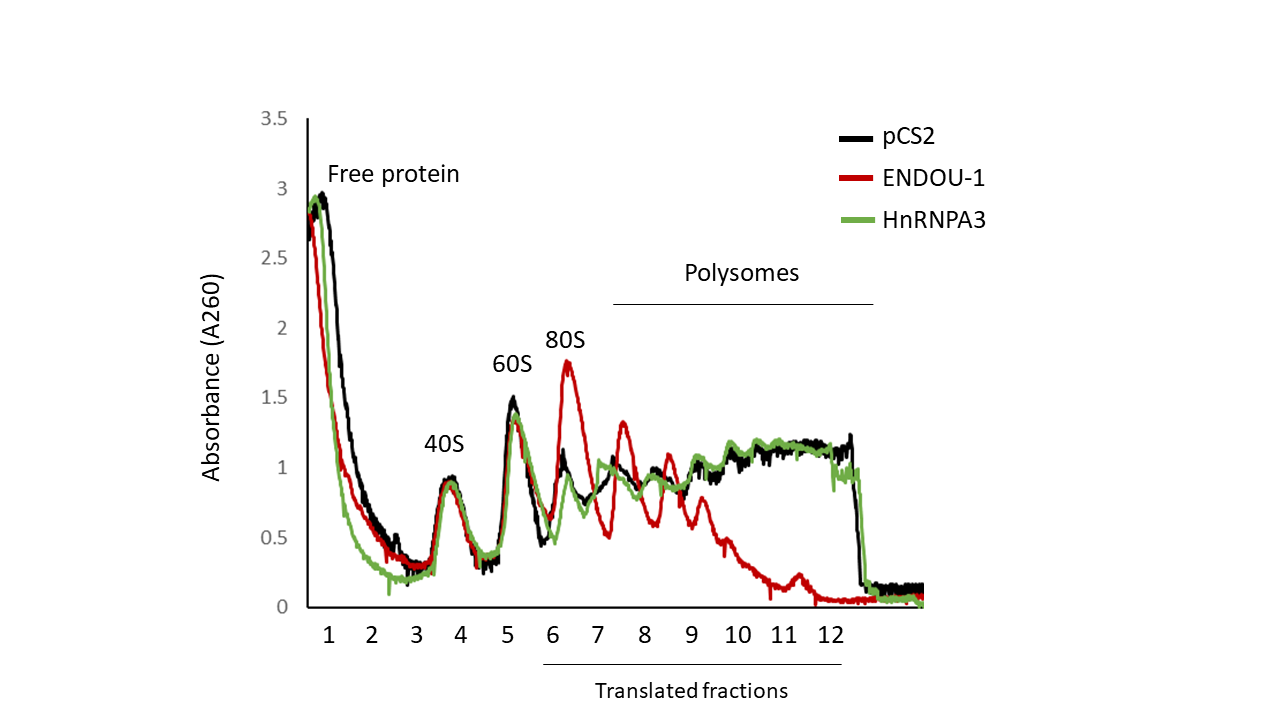


**Figure S2.** Sucrose gradient absorbance profiles from HEK293T cells transfected with an empty expression plasmid (black), a plasmid containing ENDOU-1 (red), or a plasmid containing HnRNPA3 (green). Polysome profile analysis (PPA) was performed on the lysates of transfected cells. The sucrose gradient, from top to bottom, was shown from left to right, respectively. In order, the first peak contains cytosolic-free proteins, the sedimentation of 40S and 60S ribosomal subunits, 80S monosomes, and, finally, polysomes.


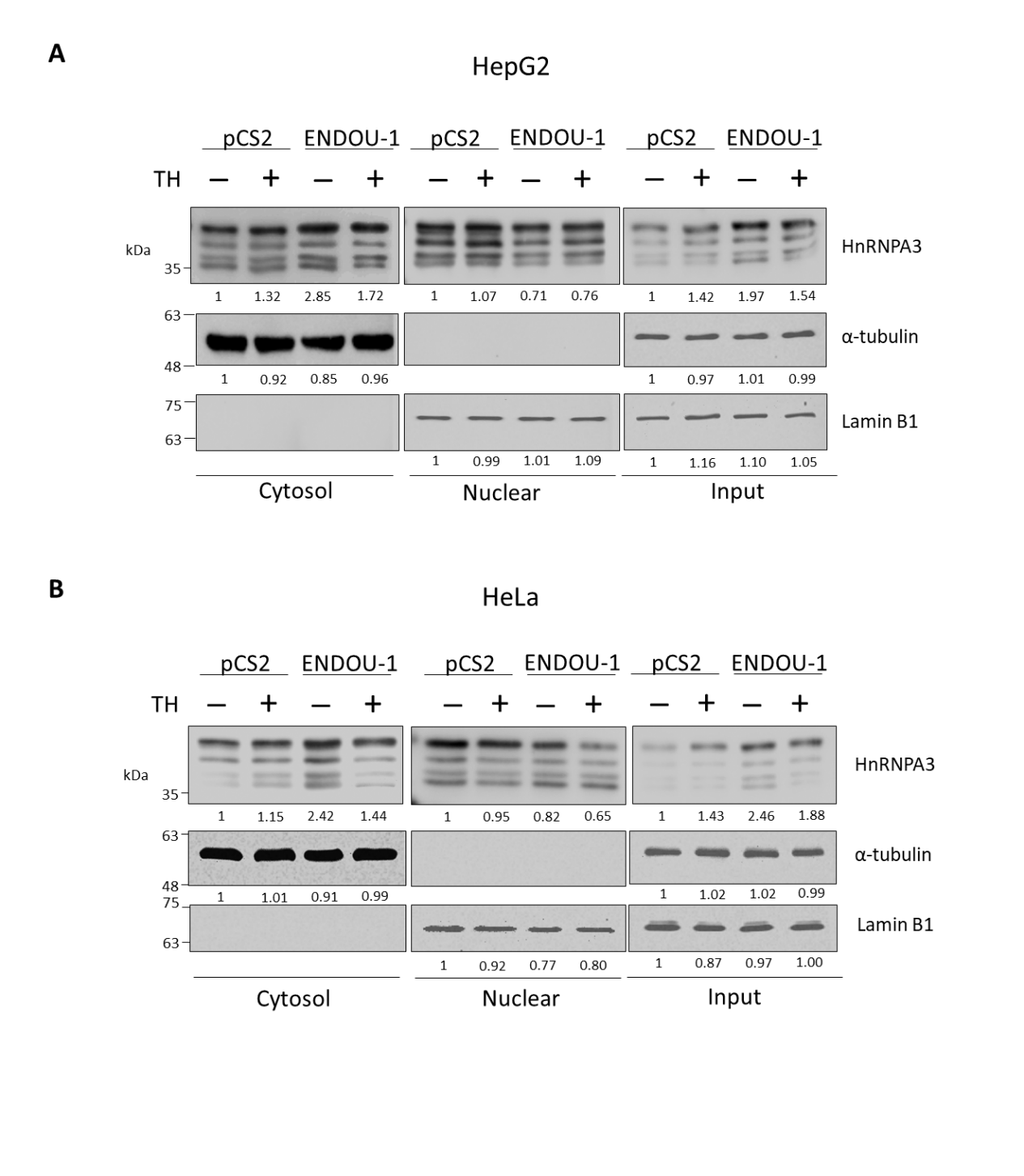


**Figure S3. Overexpression of ENDOU-1 induced HnRNPA3 shift from the nucleus to the cytoplasm.** The HepG2 (A) and HeLa (B) Cells were transfected with indicated plasmid and treated with DMSO (TH-) or TH for 6 h and then the cells were used to analyze the protein level of HnRNPA3 from whole cell lysates, cytosolic fractions, and nuclear fractions. Lamin B1 and α-tubulin served as loading controls for nuclear and cytoplasmic fractions, respectively. Protein levels relative to each internal control (α-tubulin) are presented below each lane.


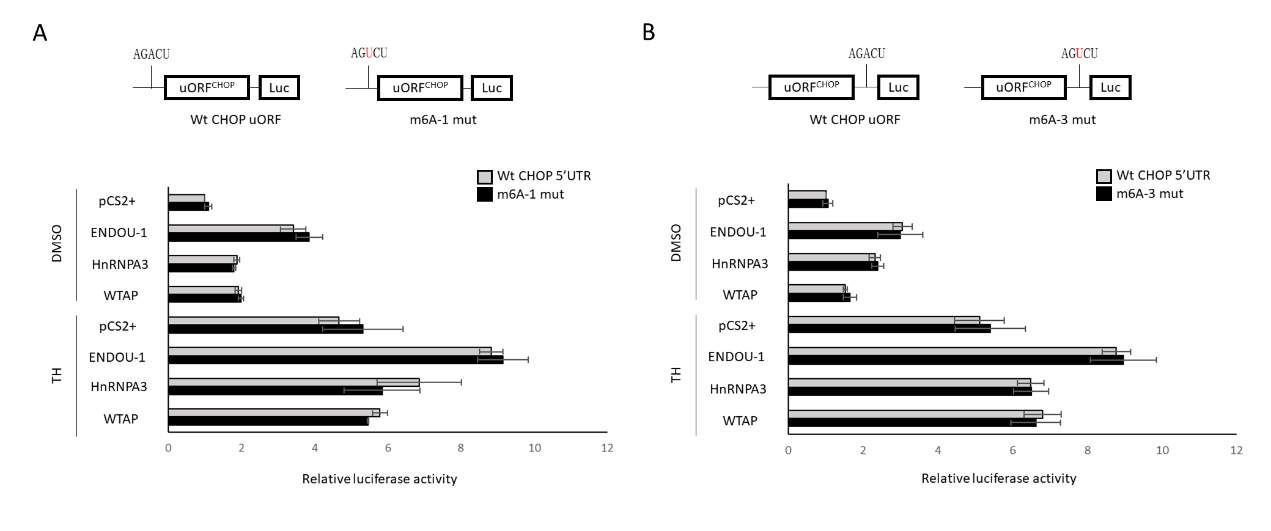


**Figure S4. m6A-1 and m6A-3 in *CHOP* 5’UTR involved in HnRNPA3/ENDOU-1/methylation-mediated translation during ER stress.**

(A). Schematic representation of wild-type (Wt) *CHOP* 5’UTR and m6A-1 mutated constructs. The luc activity of HEK293T cells transfected with the indicated plasmid, under normal (DMSO) or stress (TH) conditions. (B) Schematic representation of Wt *CHOP* 5’UTR and m6A-3 mutated constructs. The luc activity of HEK293T cells transfected with the indicated plasmid, under normal (DMSO) or stress (TH) conditions. The relative luc activity was represented by the fold increase of Fluc/Rluc ratio over that obtained from pCS2+ transfected control group normalized as 1. Data in are presented as mean ± SEM.


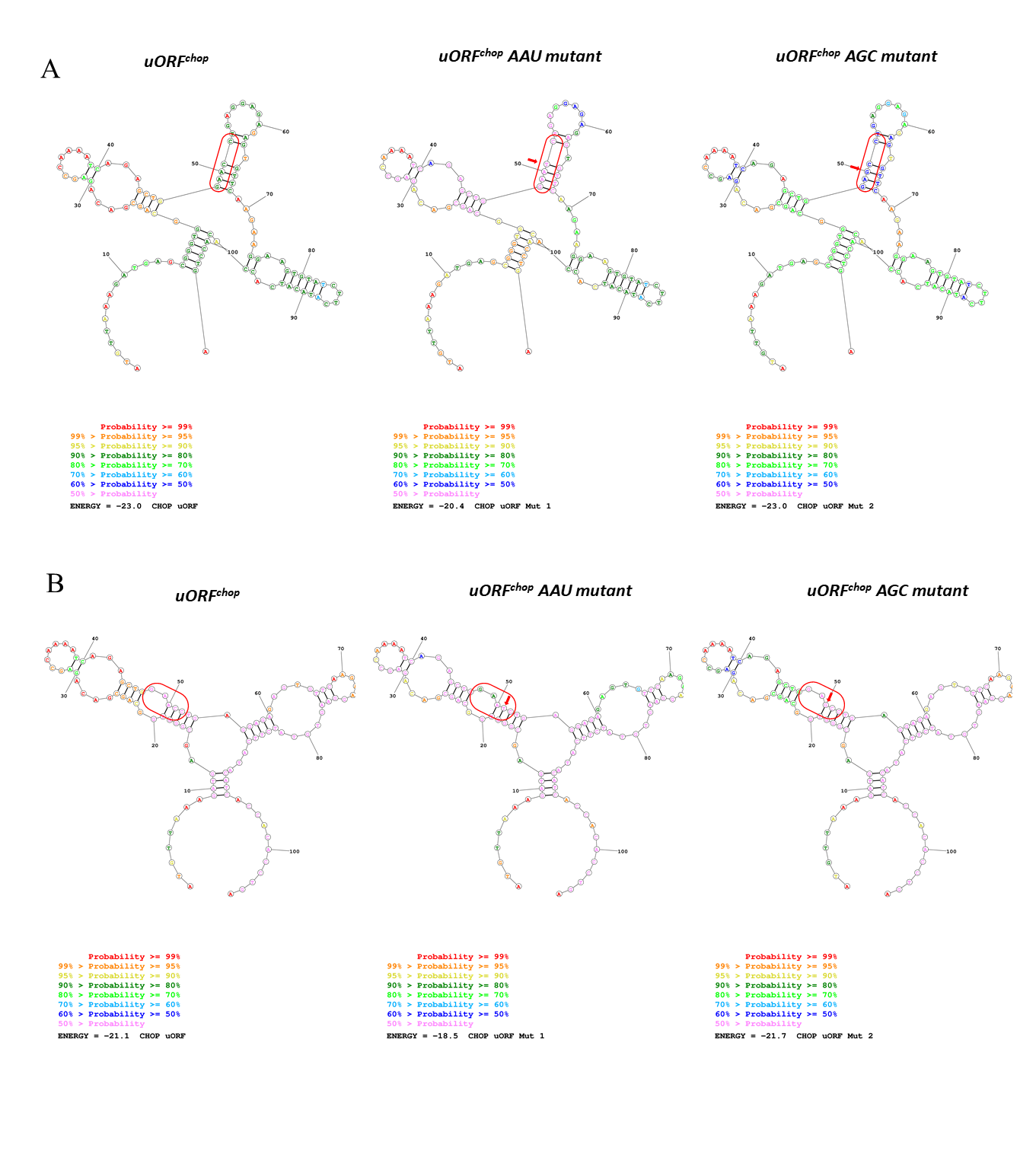


**Figure S5. Schematic representation of secondary structure of the *uORF^chop^* transcript and its mutants.**

(A, B) Two secondary RNA structures of the *uORF^chop^* transcript and its AAU and AGC mutant were predicted using Predict a Secondary Structure Web Server (<https://rna.urmc.rochester.edu/RNAstructureWeb/index.html>). The m6A-2 site was round with red circle. The red arrows indicates the mutated residue.


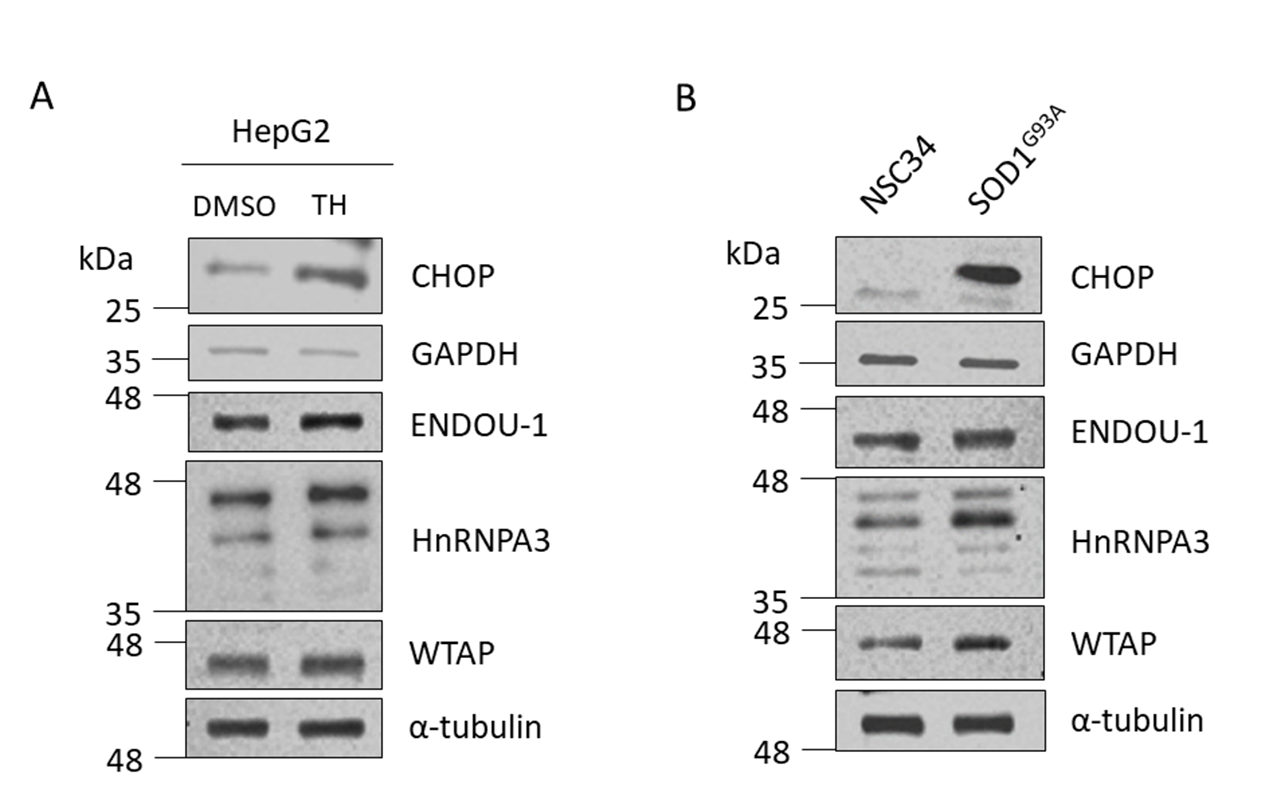


**Figure S6. Increased CHOP protein is correspondent with increased ENDOU-1, HnRNPA3 and WTAP.**

(A)HepG2 cells were treated with DMSO or Thapsigargin (TH) for 6 h and then evaluated the protein level of CHOP, ENDOU-1 HnRNPA3, WTAP. The a-tubulin gene was used as a control group. (B)The NSC34 and NSC34-SOD1G93A (mSOD1) cells were used to evaluate the protein level of CHOP, ENDOU-1 HnRNPA3, WTAP. The a-tubulin and GAPDH were used as internal controls.
