# Supplementary material for: ENDOU-1-induced cytoplasmic HnRNPA3 recognizes m6A methylation on the upstream reading frame of human CHOP transcripts to achieve maximal CHOP translation
Source: Cell Mol Life Sci. 2026 Mar 28;83(1):194. doi: 10.1007/s00018-026-06180-7 (PMC13049129; doi:10.1007/s00018-026-06180-7)
Supplement: Supplementary file 2 — Supplementary Material 2. [file 18_2026_6180_MOESM2_ESM.pdf]

Figure 1B

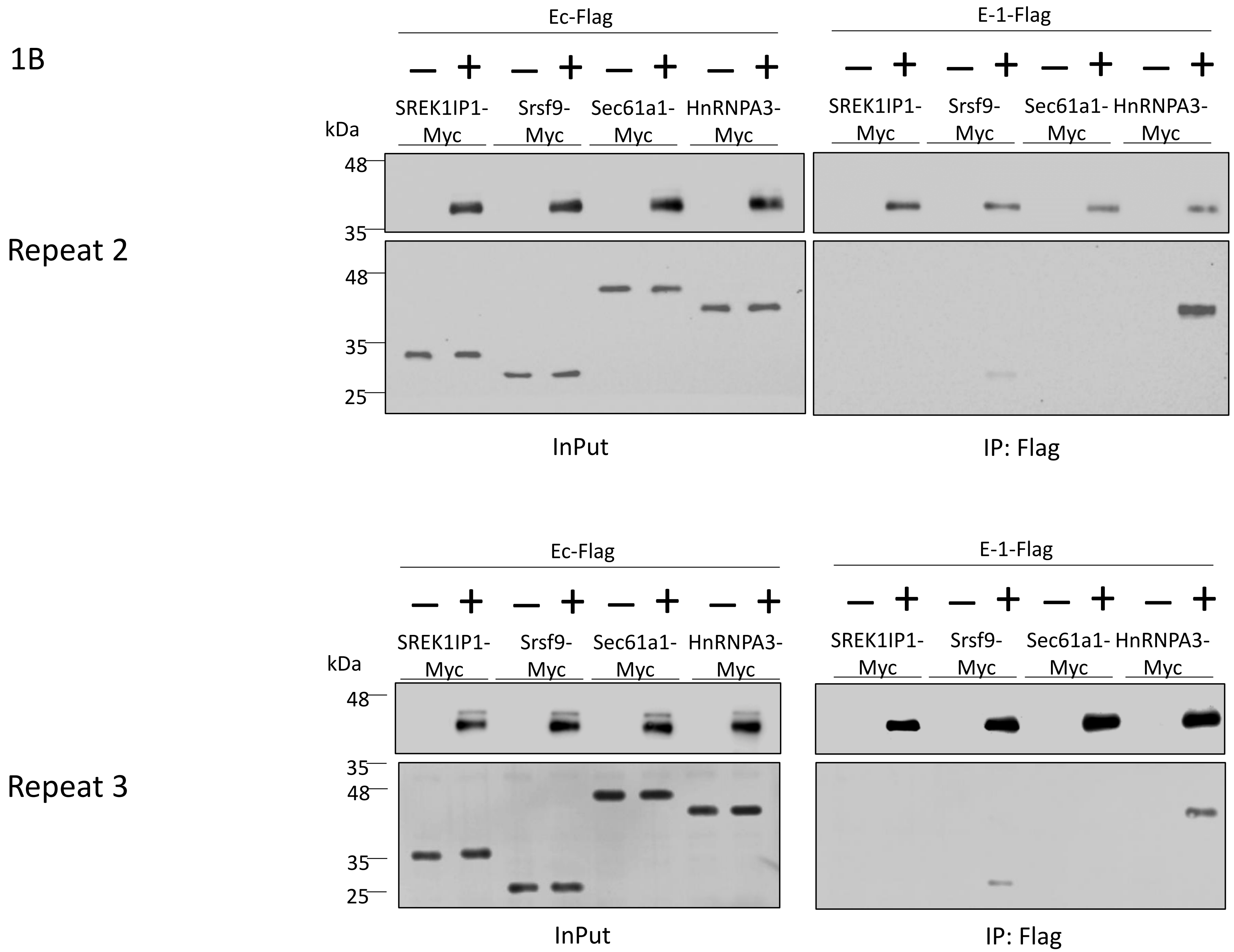

Repeat 2
Ec-Flag
E-1-Flag
– + – + – + – +
– + – + – + – +
SREK1IP1-Myc
Srsf9-Myc
Sec61a1-Myc
HnRNPA3-Myc
SREK1IP1-Myc
Srsf9-Myc
Sec61a1-Myc
HnRNPA3-Myc
kDa
48
35
48
35
25
InPut
IP: Flag
Repeat 3
Ec-Flag
E-1-Flag
– + – + – + – +
– + – + – + – +
SREK1IP1-Myc
Srsf9-Myc
Sec61a1-Myc
HnRNPA3-Myc
SREK1IP1-Myc
Srsf9-Myc
Sec61a1-Myc
HnRNPA3-Myc
kDa
48
35
48
35
25
InPut
IP: Flag

Figure 1C

Repeat 2

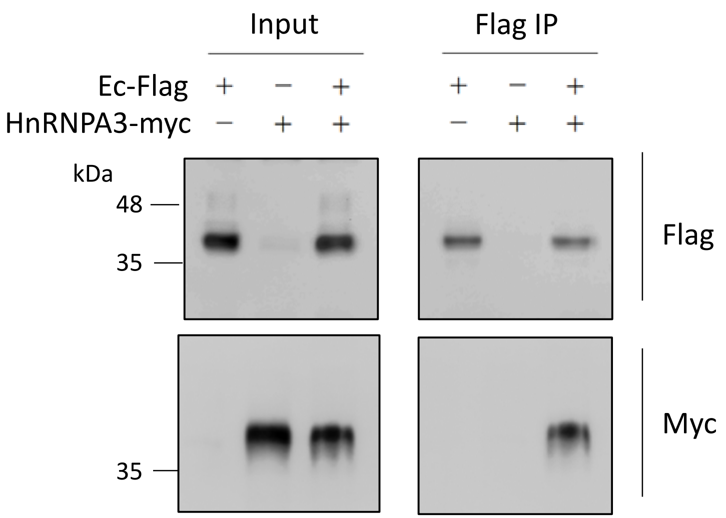

Repeat 3

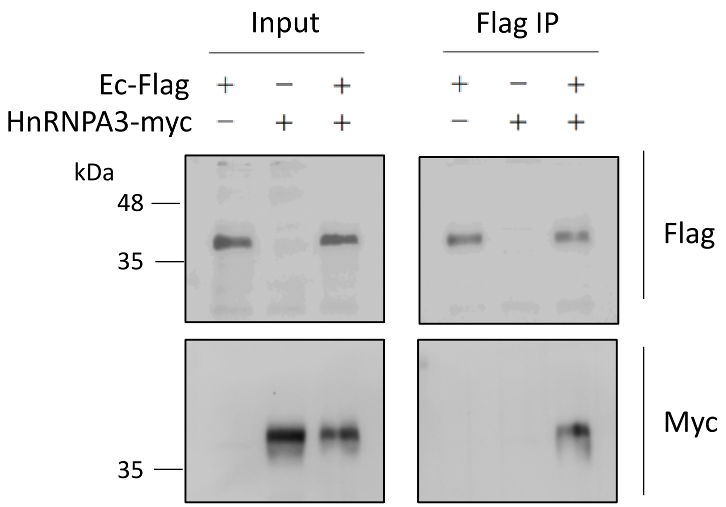

Figure 1D

Repeat 2

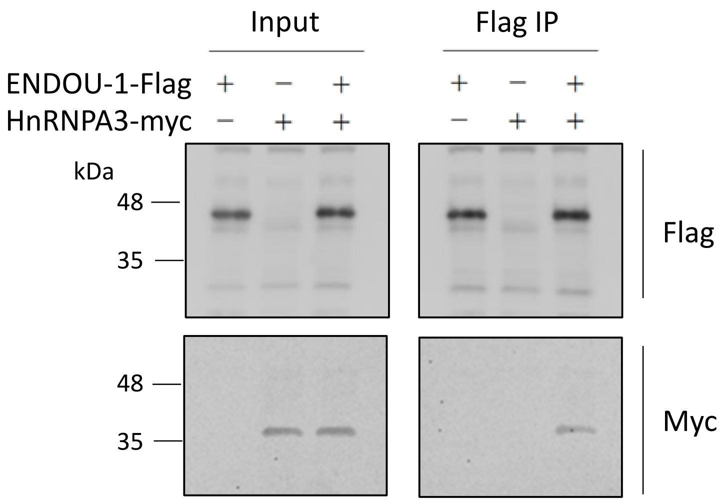

Repeat 3

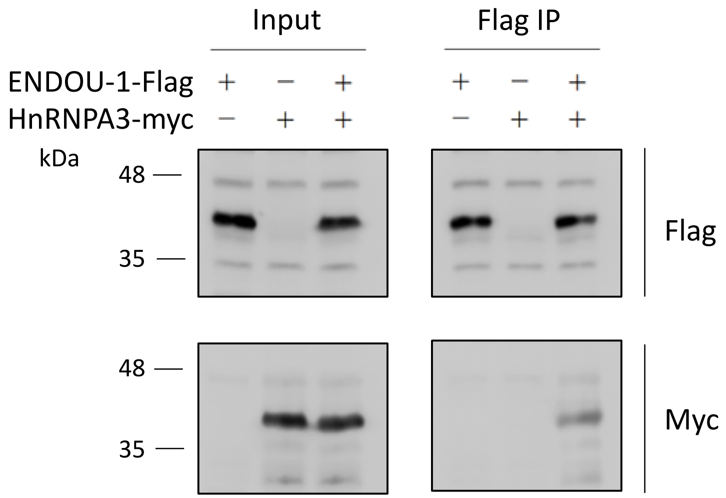

Figure 1F

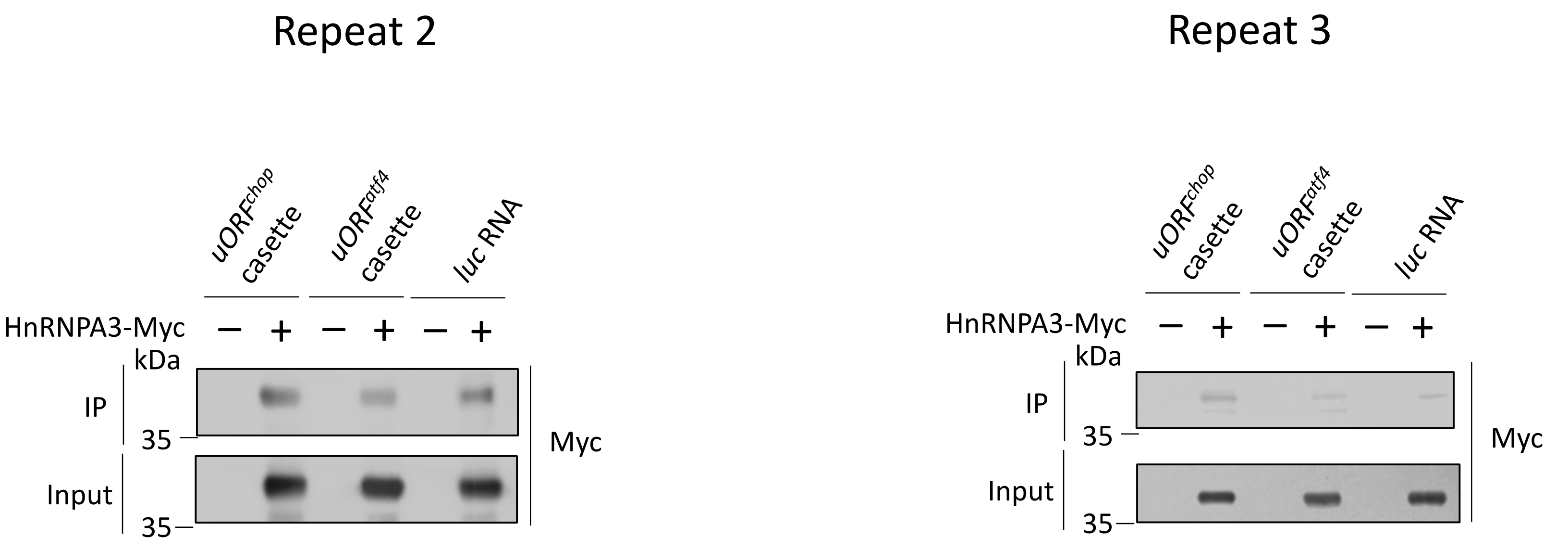
Repeat 2
uORF^chop casette
uORF^atf4 casette
luc RNA
HnRNPA3-Myc − + − + − +
kDa
IP
35
Input
35
Myc
Repeat 3
uORF^chop casette
uORF^atf4 casette
luc RNA
HnRNPA3-Myc − + − + − +
kDa
IP
35
Input
35
Myc

Figure 2D

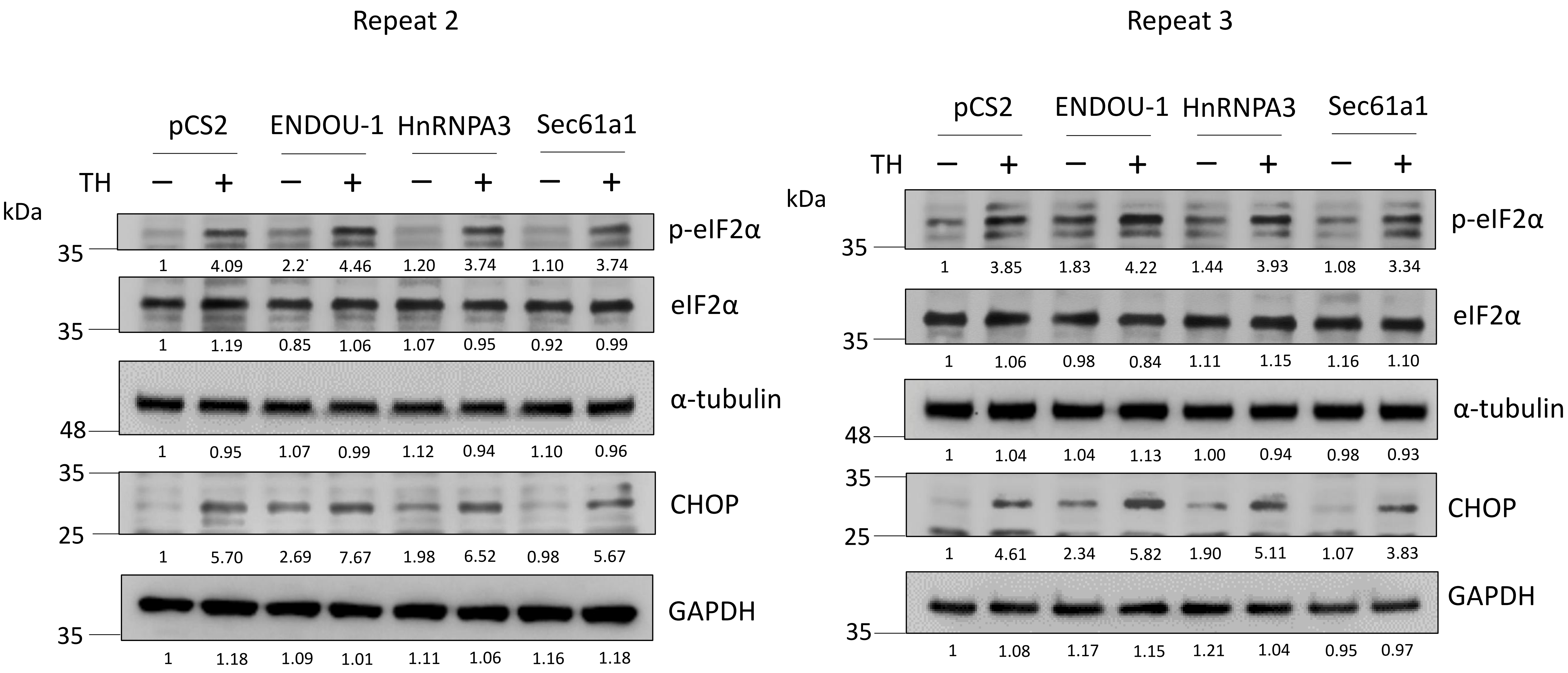

Repeat 2
pCS2
ENDOU-1
HnRNPA3
Sec61a1
TH − + − + − + − +
kDa
35
p-eIF2α
1 4.09 2.2 4.46 1.20 3.74 1.10 3.74
35
eIF2α
1 1.19 0.85 1.06 1.07 0.95 0.92 0.99
48
α-tubulin
1 0.95 1.07 0.99 1.12 0.94 1.10 0.96
35
25
CHOP
1 5.70 2.69 7.67 1.98 6.52 0.98 5.67
35
GAPDH
1 1.18 1.09 1.01 1.11 1.06 1.16 1.18
Repeat 3
pCS2
ENDOU-1
HnRNPA3
Sec61a1
TH − + − + − + − +
kDa
35
p-eIF2α
1 3.85 1.83 4.22 1.44 3.93 1.08 3.34
35
eIF2α
1 1.06 0.98 0.84 1.11 1.15 1.16 1.10
48
α-tubulin
1 1.04 1.04 1.13 1.00 0.94 0.98 0.93
35
25
CHOP
1 4.61 2.34 5.82 1.90 5.11 1.07 3.83
GAPDH
35
1 1.08 1.17 1.15 1.21 1.04 0.95 0.97

Figure 2G

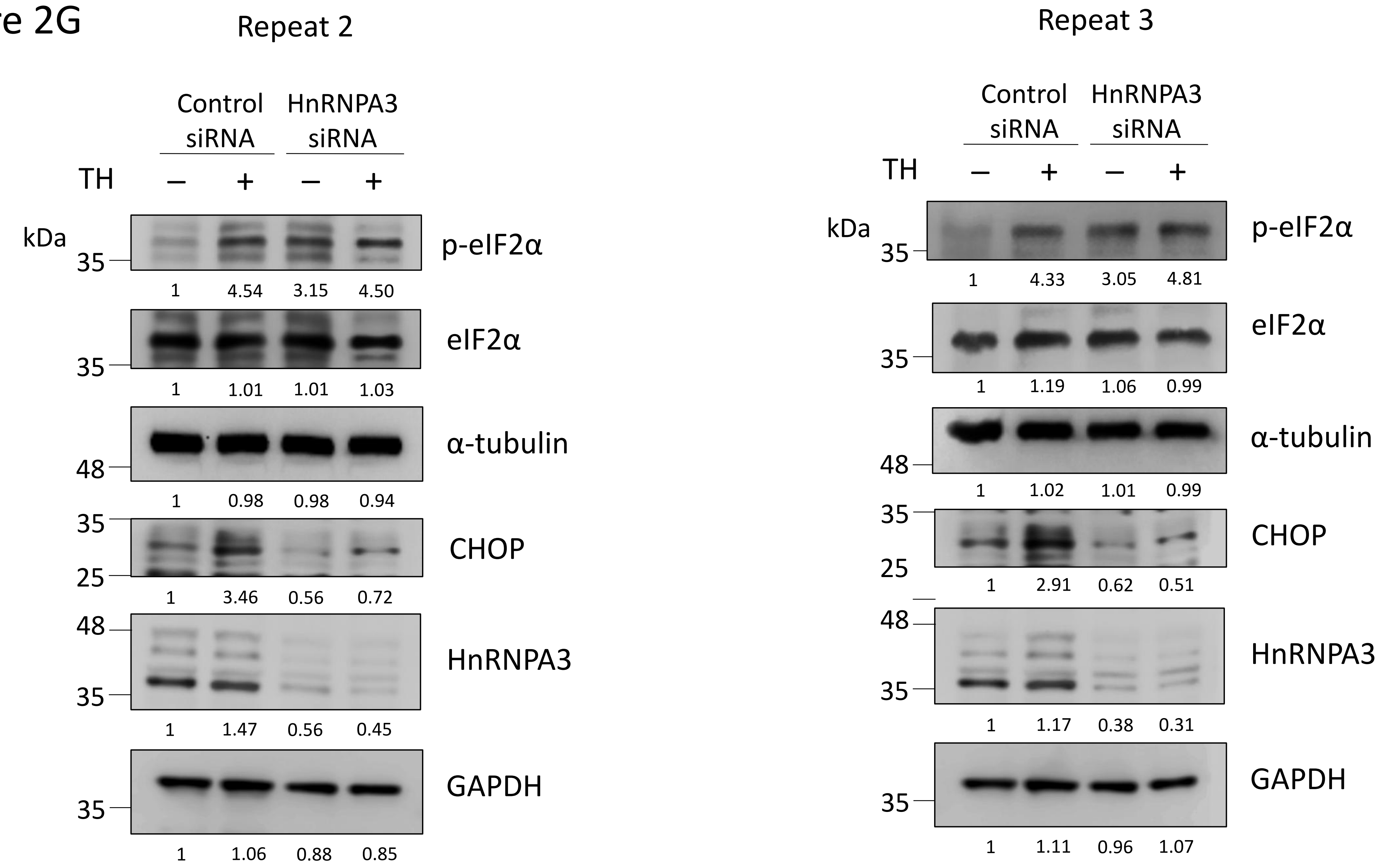
Repeat 2
Control siRNA
HnRNPA3 siRNA
TH
–
+
–
+
kDa
35
p-eIF2α
1
4.54
3.15
4.50
35
eIF2α
1
1.01
1.01
1.03
48
α-tubulin
1
0.98
0.98
0.94
35
25
CHOP
1
3.46
0.56
0.72
48
35
HnRNPA3
1
1.47
0.56
0.45
35
GAPDH
1
1.06
0.88
0.85
Repeat 3
Control siRNA
HnRNPA3 siRNA
TH
–
+
–
+
kDa
35
p-eIF2α
1
4.33
3.05
4.81
35
eIF2α
1
1.19
1.06
0.99
48
α-tubulin
1
1.02
1.01
0.99
35
25
CHOP
1
2.91
0.62
0.51
48
35
HnRNPA3
1
1.17
0.38
0.31
35
GAPDH
1
1.11
0.96
1.07

Figure 3A

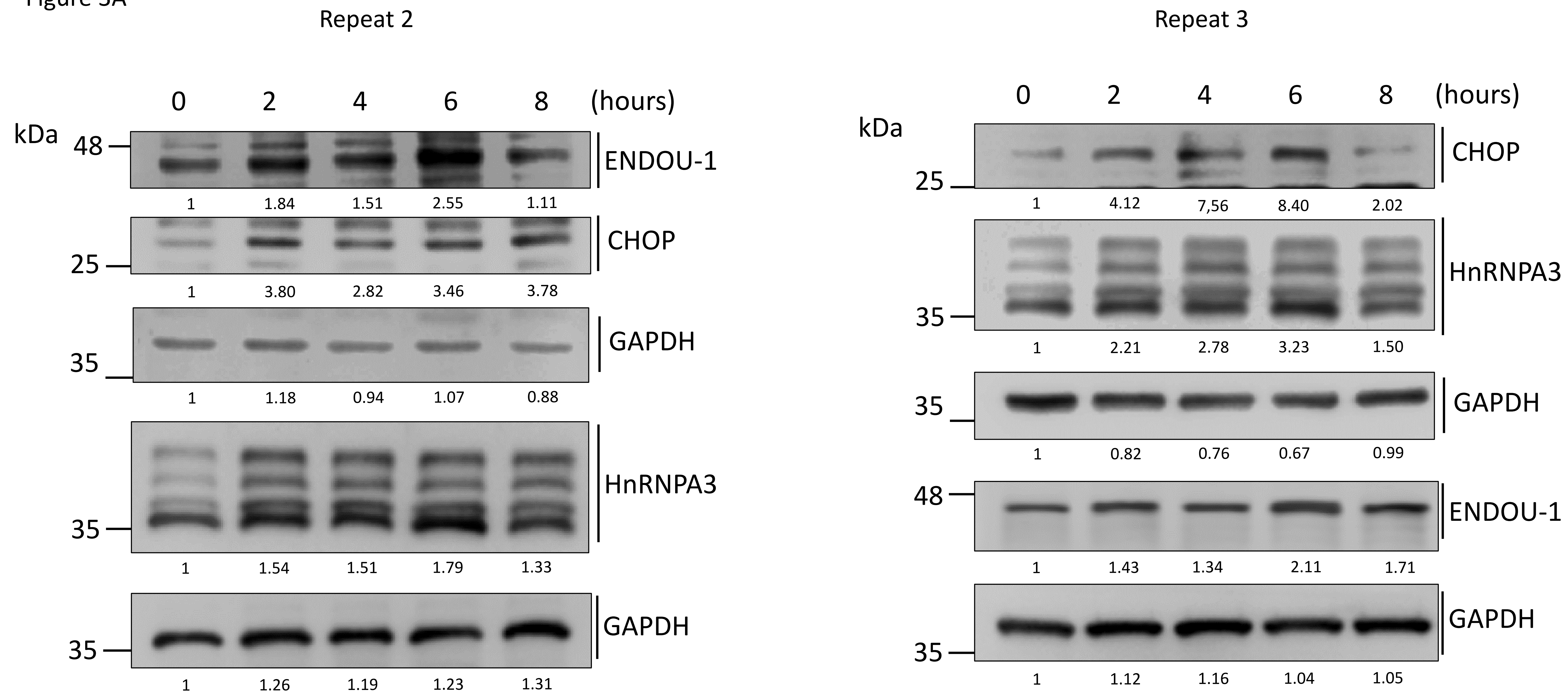

Repeat 2
0 2 4 6 8 (hours)
kDa
48
ENDOU-1
1 1.84 1.51 2.55 1.11
25
CHOP
1 3.80 2.82 3.46 3.78
35
GAPDH
1 1.18 0.94 1.07 0.88
35
HnRNPA3
1 1.54 1.51 1.79 1.33
35
GAPDH
1 1.26 1.19 1.23 1.31
Repeat 3
0 2 4 6 8 (hours)
kDa
25
CHOP
1 4.12 7,56 8.40 2.02
35
HnRNPA3
1 2.21 2.78 3.23 1.50
35
GAPDH
1 0.82 0.76 0.67 0.99
48
ENDOU-1
1 1.43 1.34 2.11 1.71
35
GAPDH
1 1.12 1.16 1.04 1.05

Figure 3B

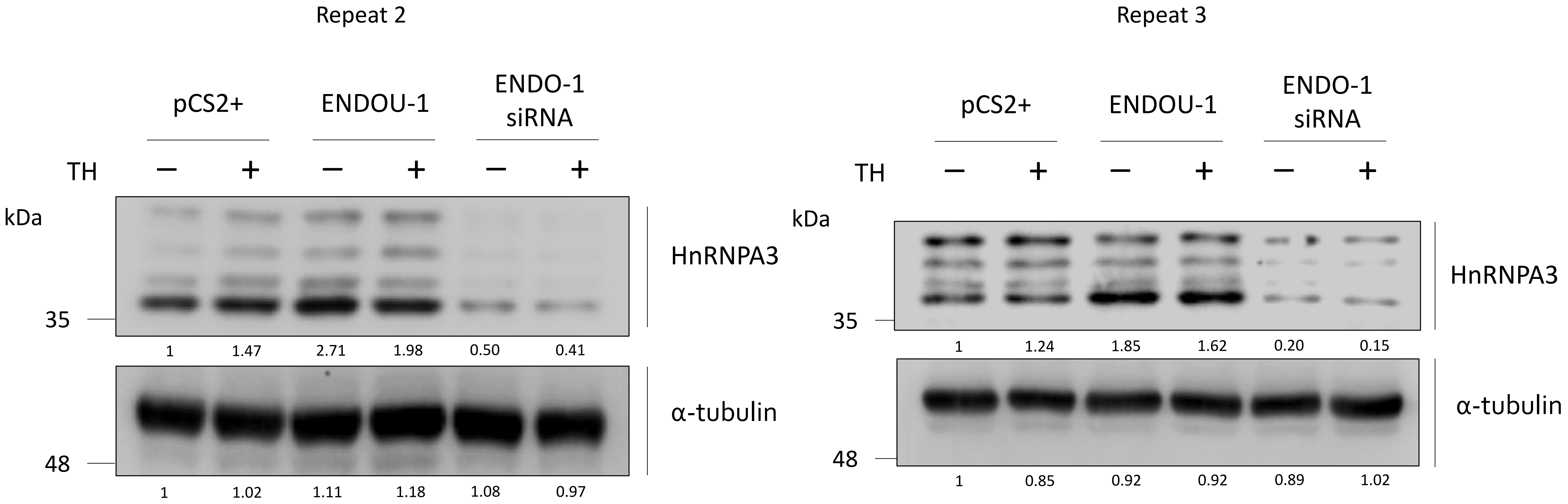
Repeat 2
pCS2+
ENDOU-1
ENDO-1 siRNA
TH
−
+
−
+
−
+
kDa
HnRNPA3
35
1
1.47
2.71
1.98
0.50
0.41
α-tubulin
48
1
1.02
1.11
1.18
1.08
0.97
Repeat 3
pCS2+
ENDOU-1
ENDO-1 siRNA
TH
−
+
−
+
−
+
kDa
HnRNPA3
35
1
1.24
1.85
1.62
0.20
0.15
α-tubulin
48
1
0.85
0.92
0.92
0.89
1.02

Figure 3C

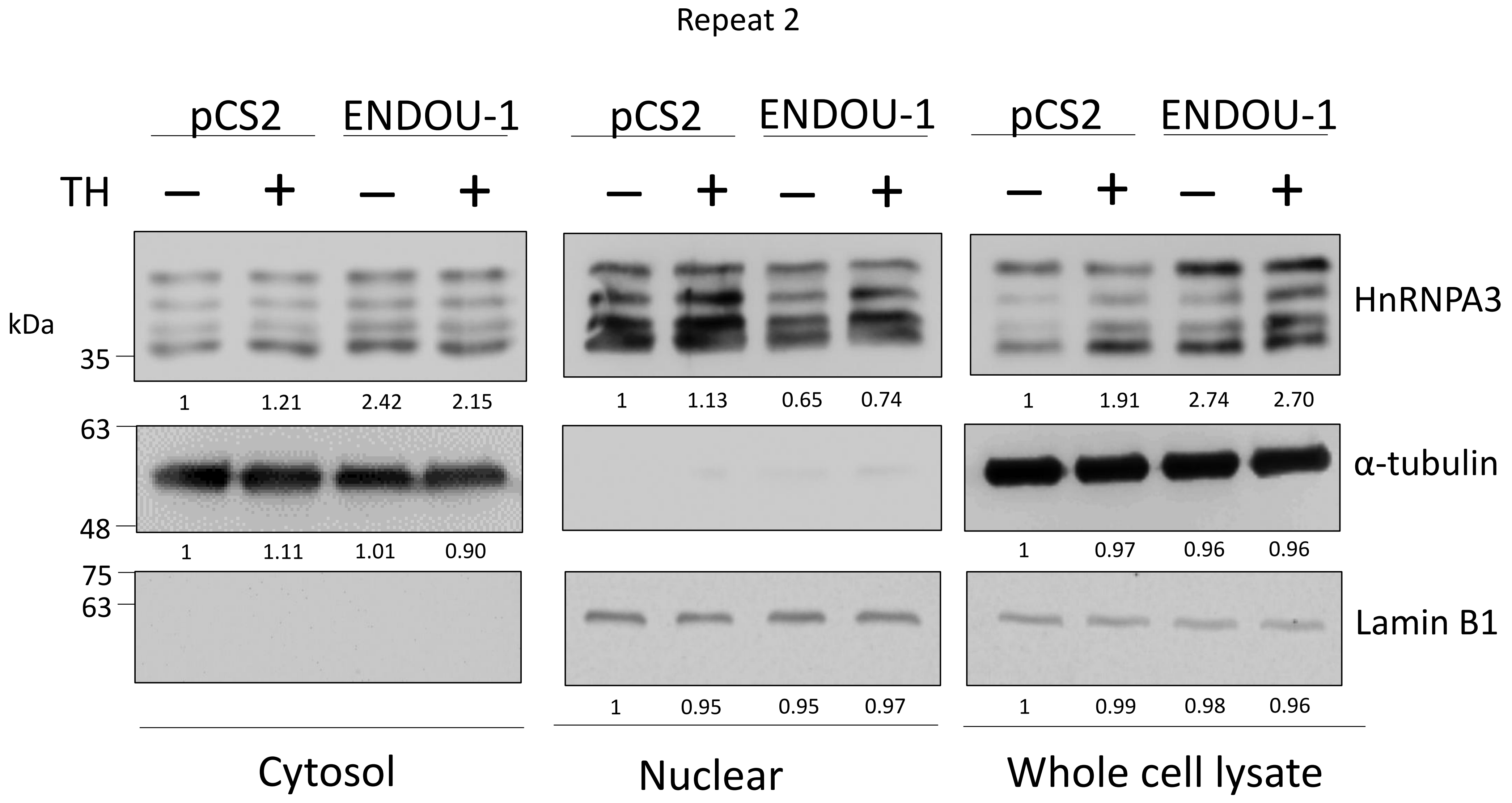

Repeat 2
pCS2
ENDOU-1
pCS2
ENDOU-1
pCS2
ENDOU-1
TH
–
+
–
+
–
+
–
+
–
+
–
+
kDa
35
HnRNPA3
1
1.21
2.42
2.15
1
1.13
0.65
0.74
1
1.91
2.74
2.70
63
48
α-tubulin
1
1.11
1.01
0.90
1
0.97
0.96
0.96
75
63
Lamin B1
1
0.95
0.95
0.97
1
0.99
0.98
0.96
Cytosol
Nuclear
Whole cell lysate

Figure 3C

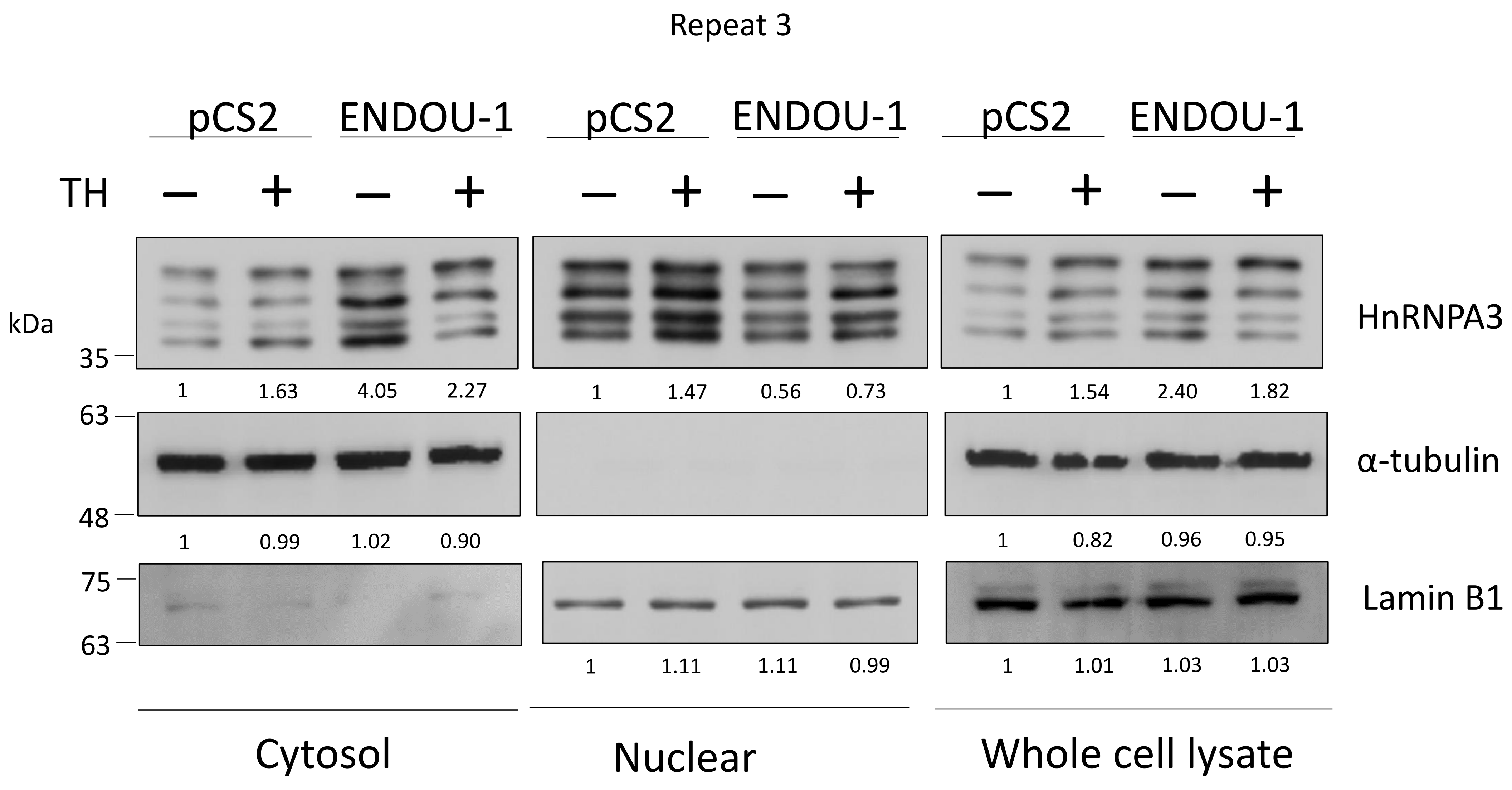
Repeat 3
pCS2
ENDOU-1
pCS2
ENDOU-1
pCS2
ENDOU-1
TH
kDa
35
63
48
75
63
HnRNPA3
α-tubulin
Lamin B1
1 1.63 4.05 2.27
1 1.47 0.56 0.73
1 1.54 2.40 1.82
1 0.99 1.02 0.90
1 0.82 0.96 0.95
1 1.11 1.11 0.99
1 1.01 1.03 1.03
Cytosol
Nuclear
Whole cell lysate

Figure 3E

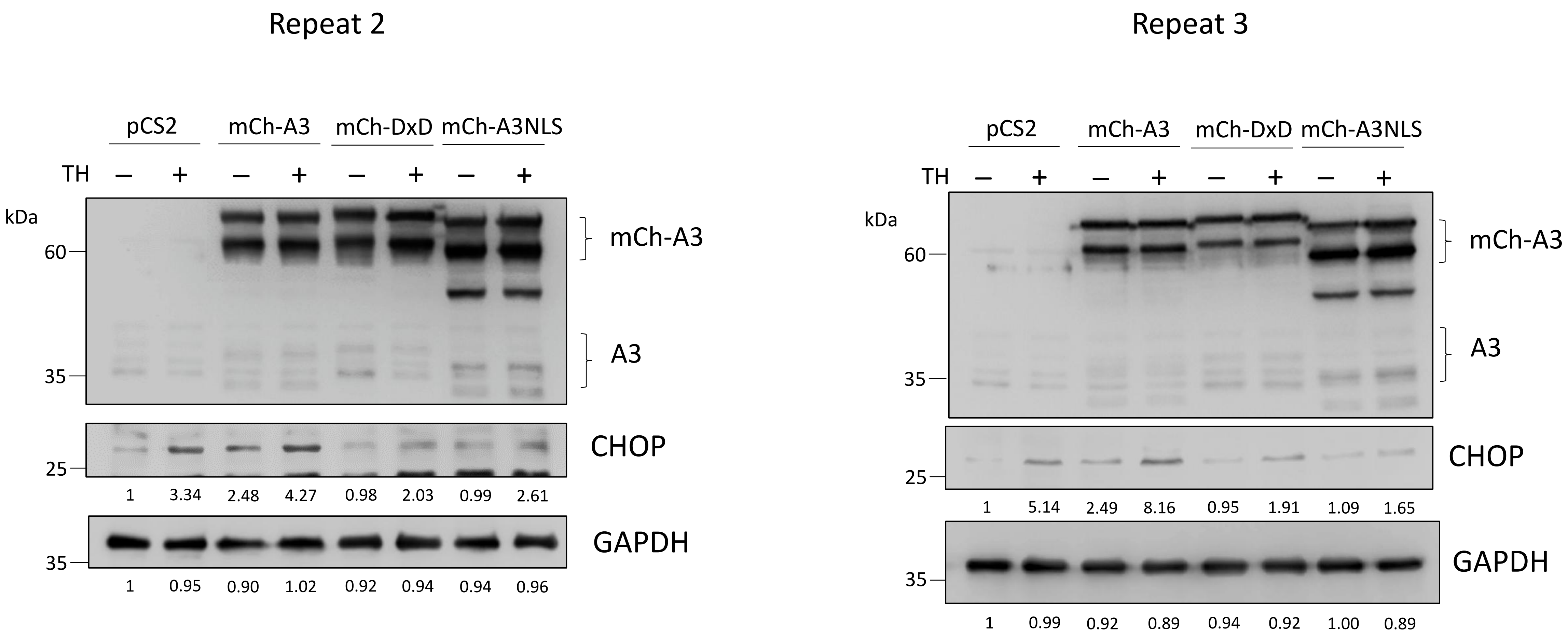
Repeat 2
pCS2
mCh-A3
mCh-DxD
mCh-A3NLS
TH − + − + − + − +
kDa
60
35
mCh-A3
A3
CHOP
25
1 3.34 2.48 4.27 0.98 2.03 0.99 2.61
GAPDH
35
1 0.95 0.90 1.02 0.92 0.94 0.94 0.96
Repeat 3
pCS2
mCh-A3
mCh-DxD
mCh-A3NLS
TH − + − + − + − +
kDa
60
35
mCh-A3
A3
CHOP
25
1 5.14 2.49 8.16 0.95 1.91 1.09 1.65
GAPDH
35
1 0.99 0.92 0.89 0.94 0.92 1.00 0.89
