# Supplementary material for: ENDOU-1-induced cytoplasmic HnRNPA3 recognizes m6A methylation on the upstream reading frame of human CHOP transcripts to achieve maximal CHOP translation
Source: Cell Mol Life Sci. 2026 Mar 28;83(1):194. doi: 10.1007/s00018-026-06180-7 (PMC13049129; doi:10.1007/s00018-026-06180-7)
Supplement: Supplementary file 3 — Supplementary Material 3. [file 18_2026_6180_MOESM3_ESM.pdf]

Figure 1

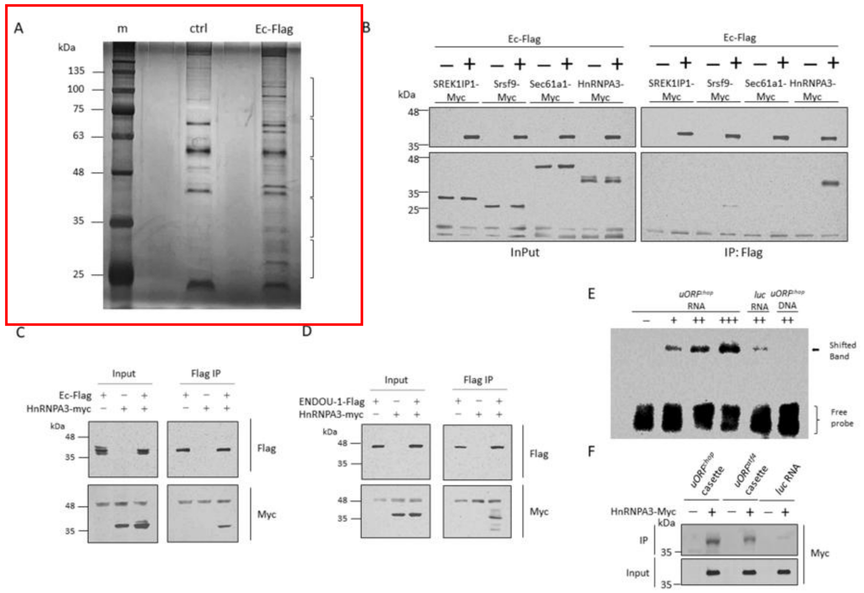

A
m
ctrl
Ec-Flag
kDa
135
100
75
63
48
35
25
B
Ec-Flag
SREK1IP1-Myc
Srsf9-Myc
Sec61a1-Myc
HnRNPA3-Myc
InPut
IP: Flag
C
Input
Flag IP
Ec-Flag
HnRNPA3-myc
Flag
Myc
D
ENDOU-1-Flag
E
Shifted Band
Free probe
F
HnRNPA3-Myc
IP
Input

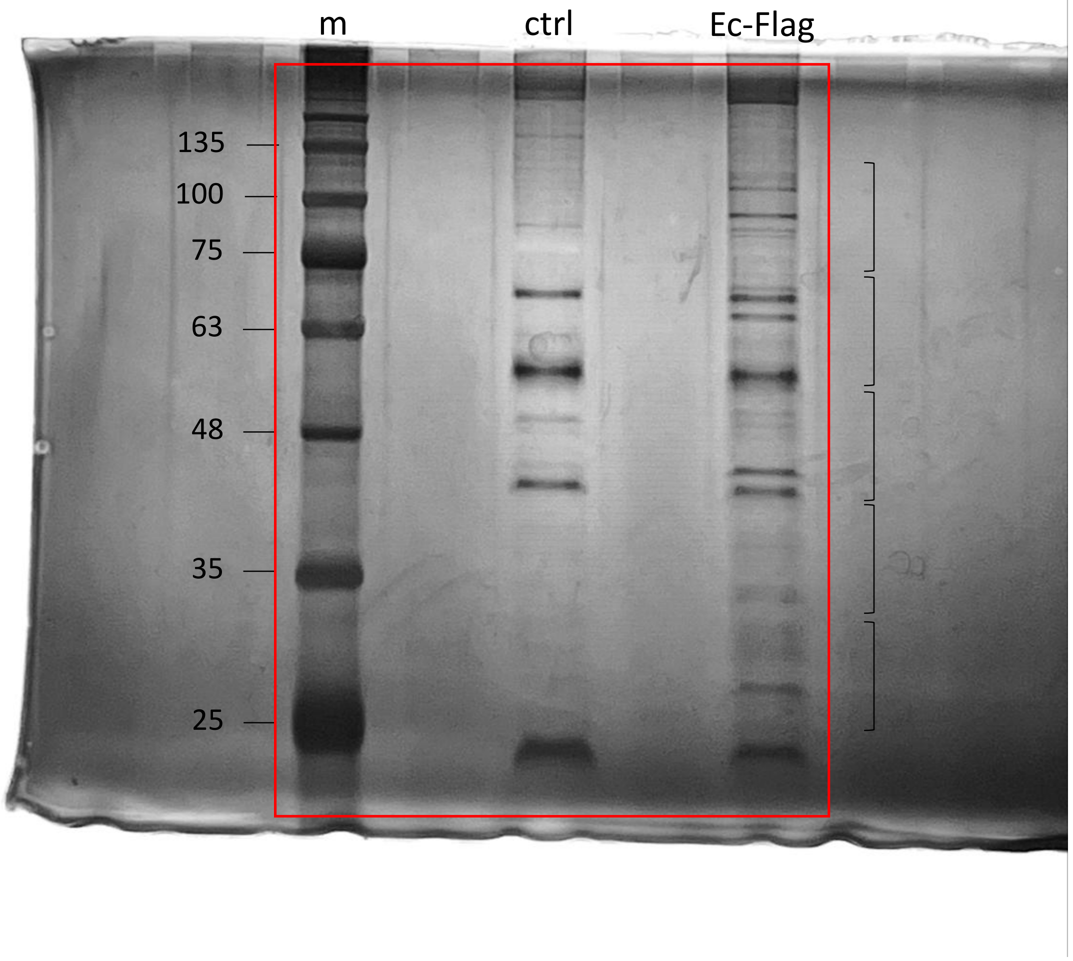

m
ctrl
Ec-Flag
135
100
75
63
48
35
25

Figure 1

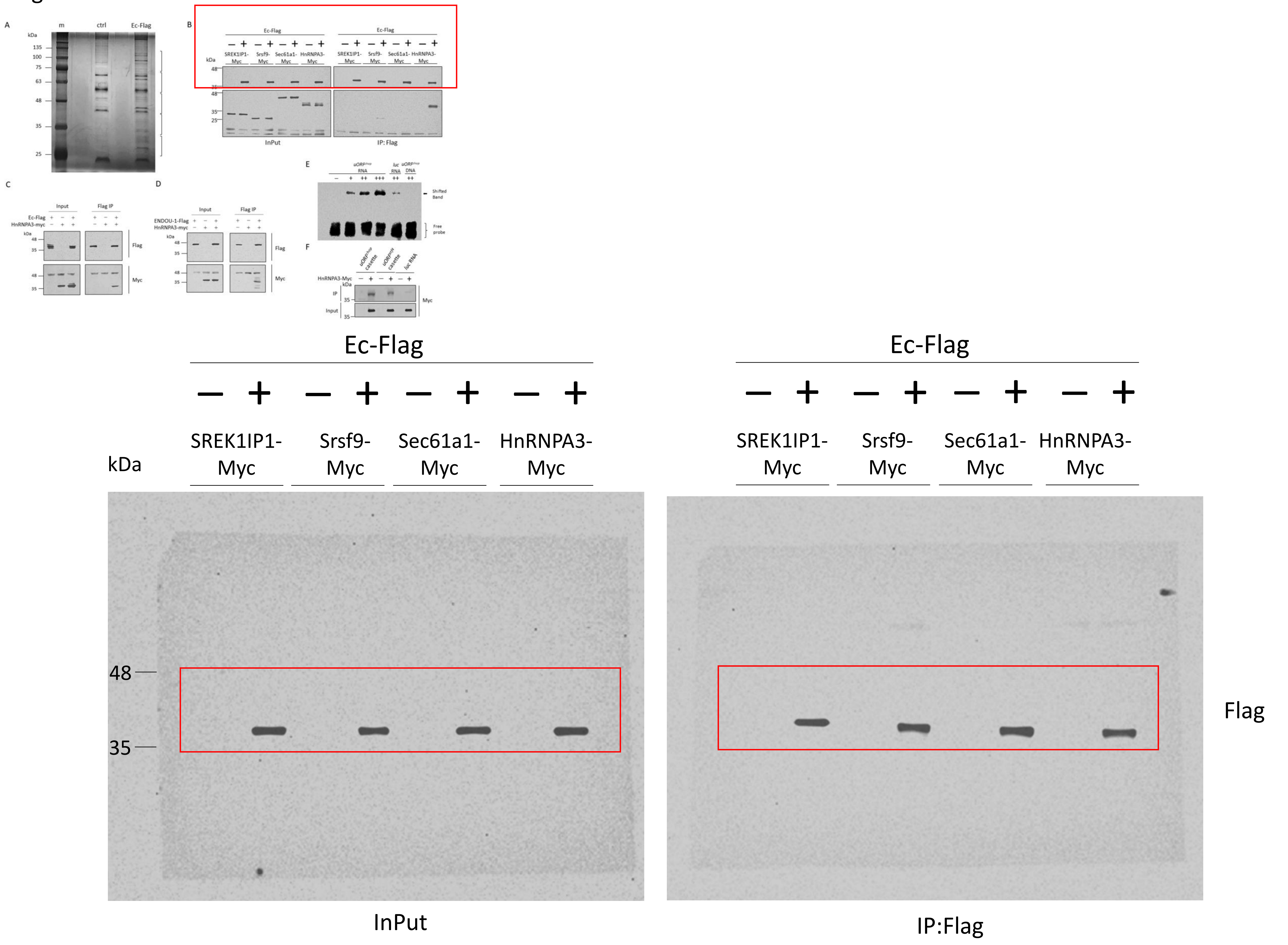

A
m
ctrl
Ec-Flag
kDa
135
100
75
63
48
35
25
B
C
D
E
F
Ec-Flag
SREK1IP1-Myc
Srsf9-Myc
Sec61a1-Myc
HnRNPA3-Myc
kDa
48
35
InPut
IP:Flag
Flag

Figure 1

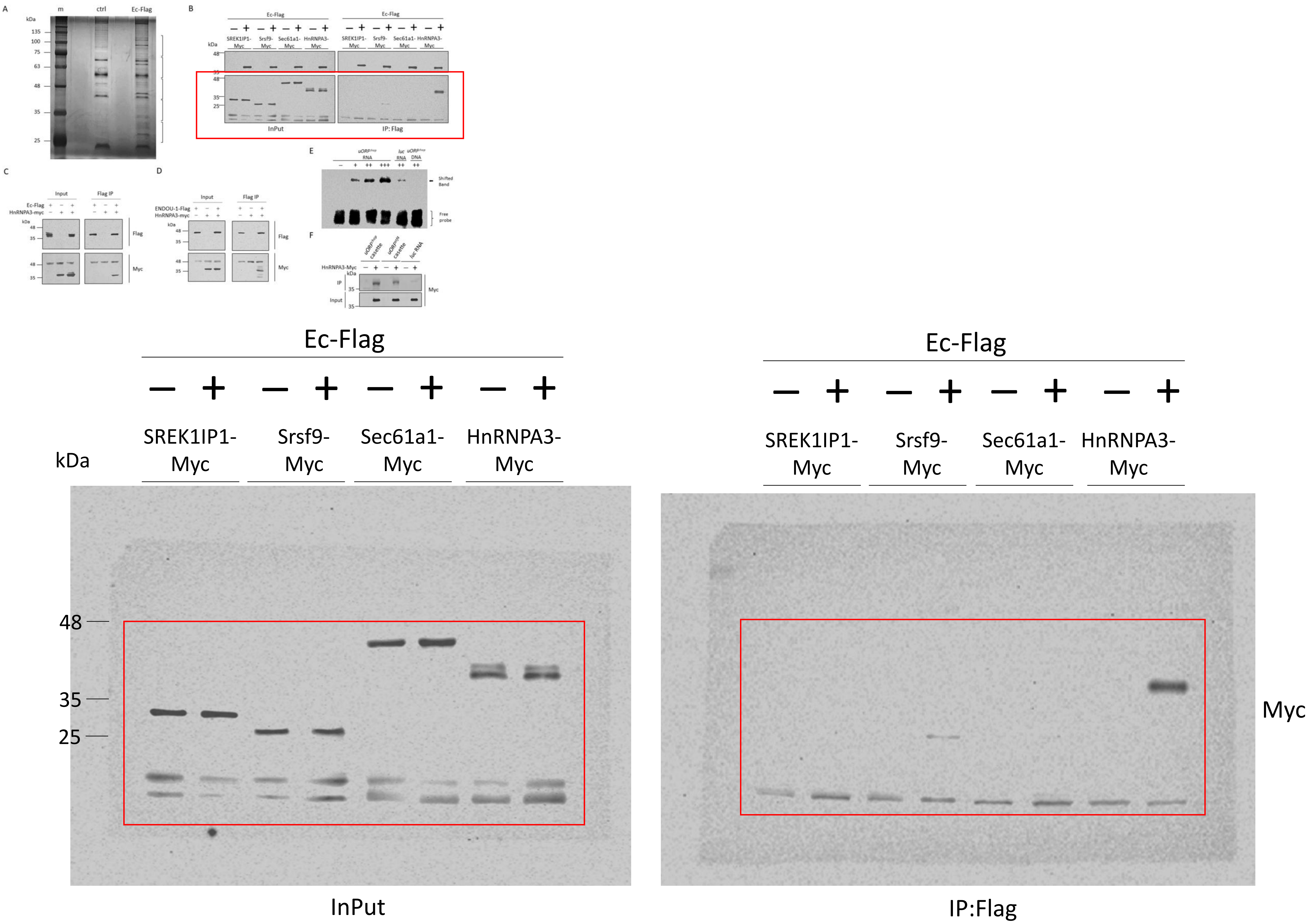

A
B
C
D
E
F
Ec-Flag
SREK1IP1-Myc
Srsf9-Myc
Sec61a1-Myc
HnRNPA3-Myc
kDa
48
35
25
Myc
InPut
IP:Flag

Figure 1

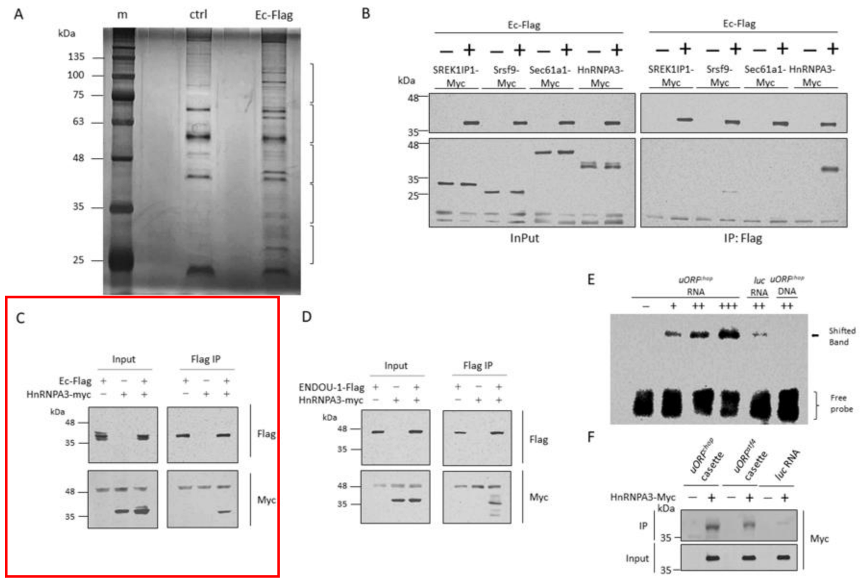

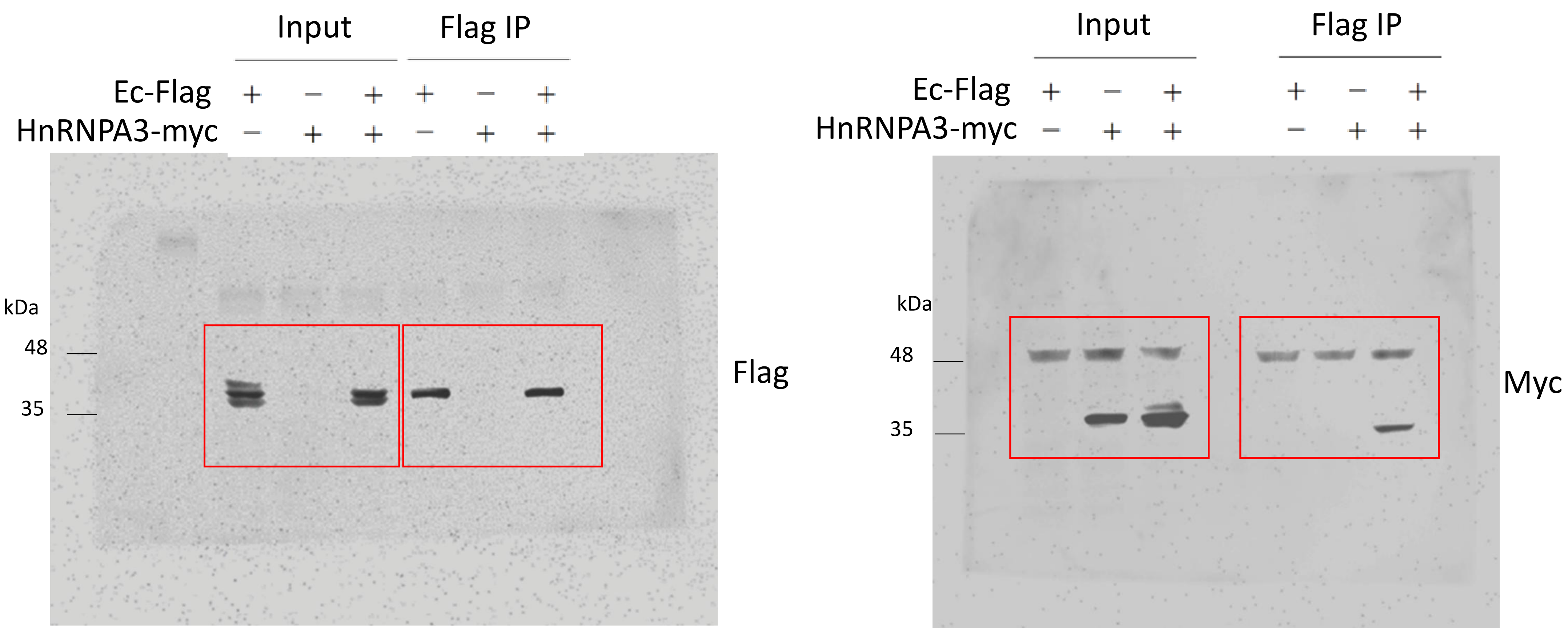

Input
Flag IP
Ec-Flag
HnRNPA3-myc
kDa
48
35
Flag
Input
Flag IP
Ec-Flag
HnRNPA3-myc
kDa
48
35
Myc

Figure 1

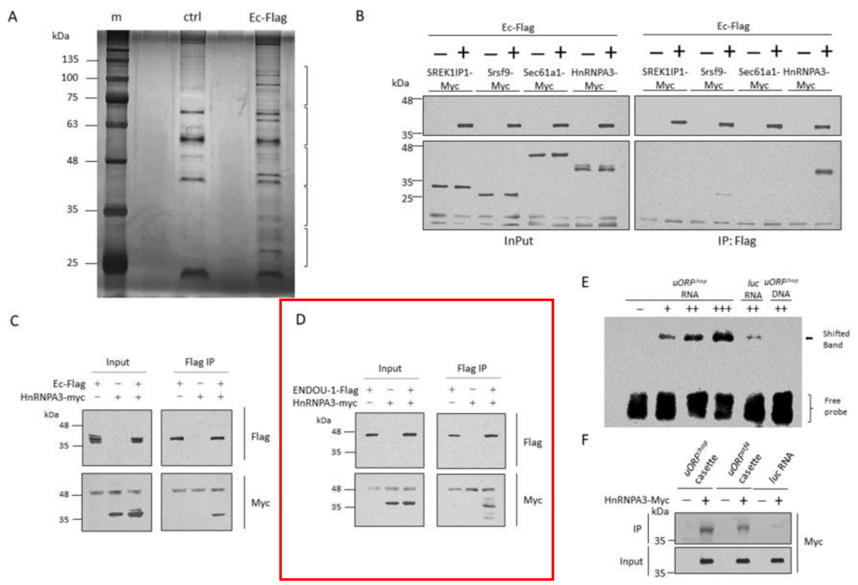

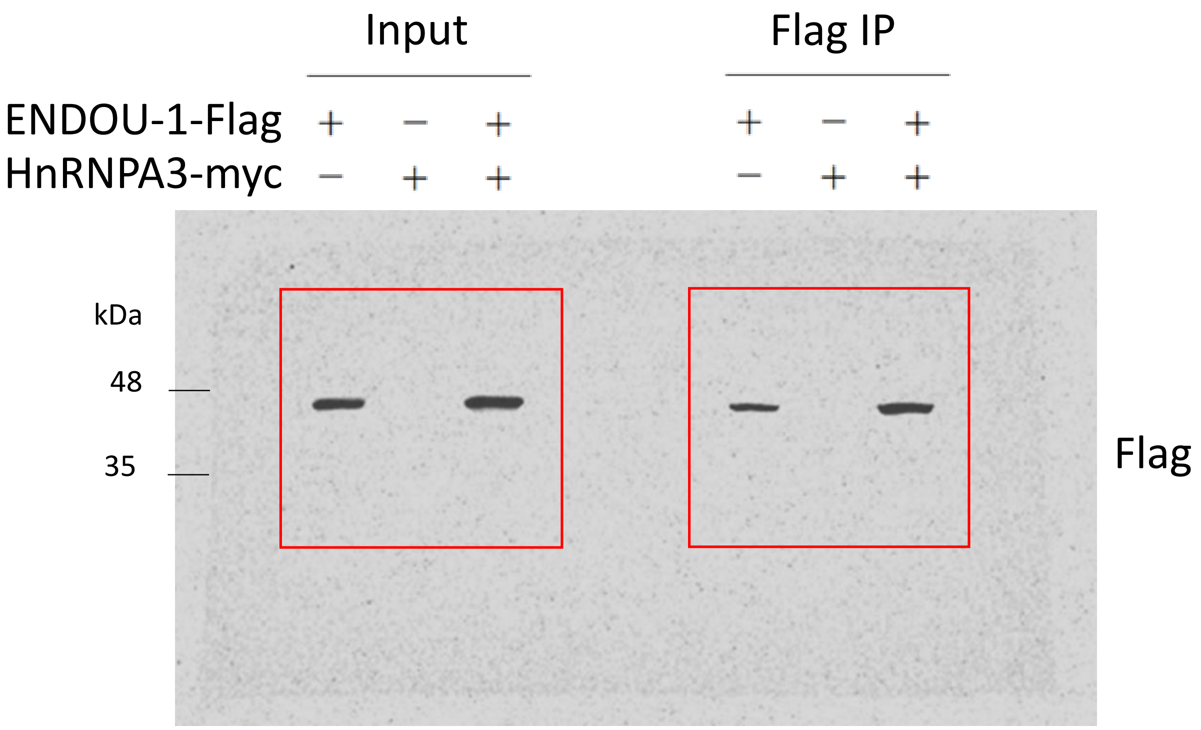
Input
Flag IP
ENDOU-1-Flag + − + + − +
HnRNPA3-myc − + + − + +
kDa
48
35
Flag

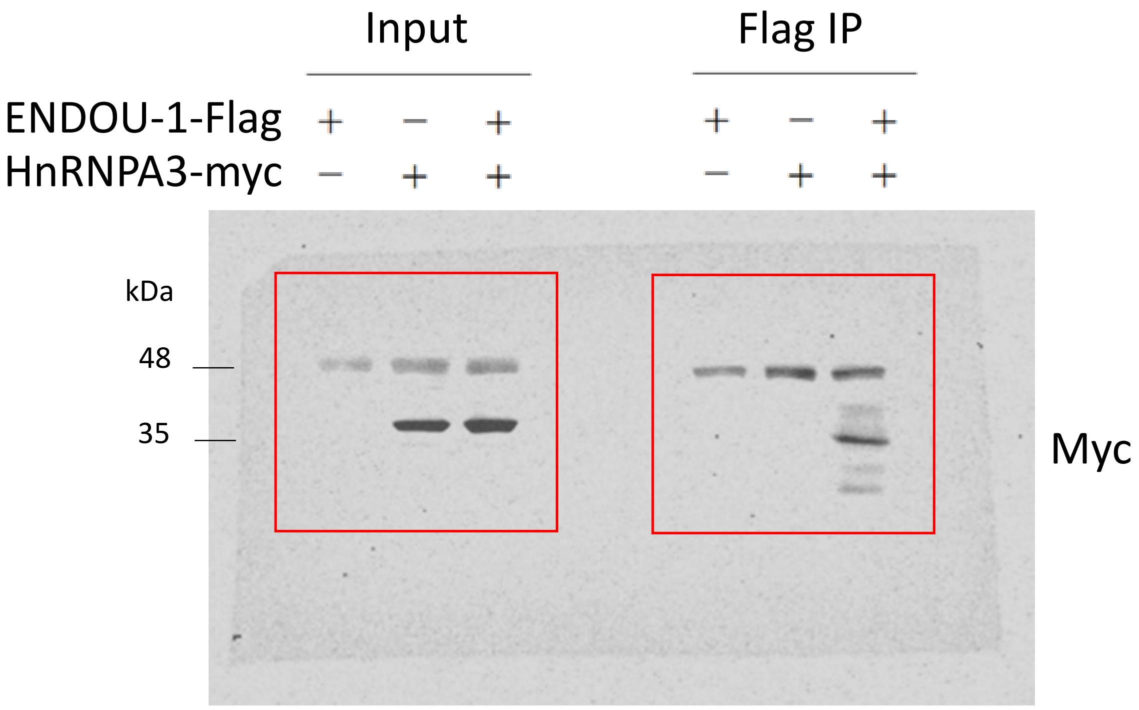
Input
Flag IP
ENDOU-1-Flag + − + + − +
HnRNPA3-myc − + + − + +
kDa
48
35
Myc

Figure 1

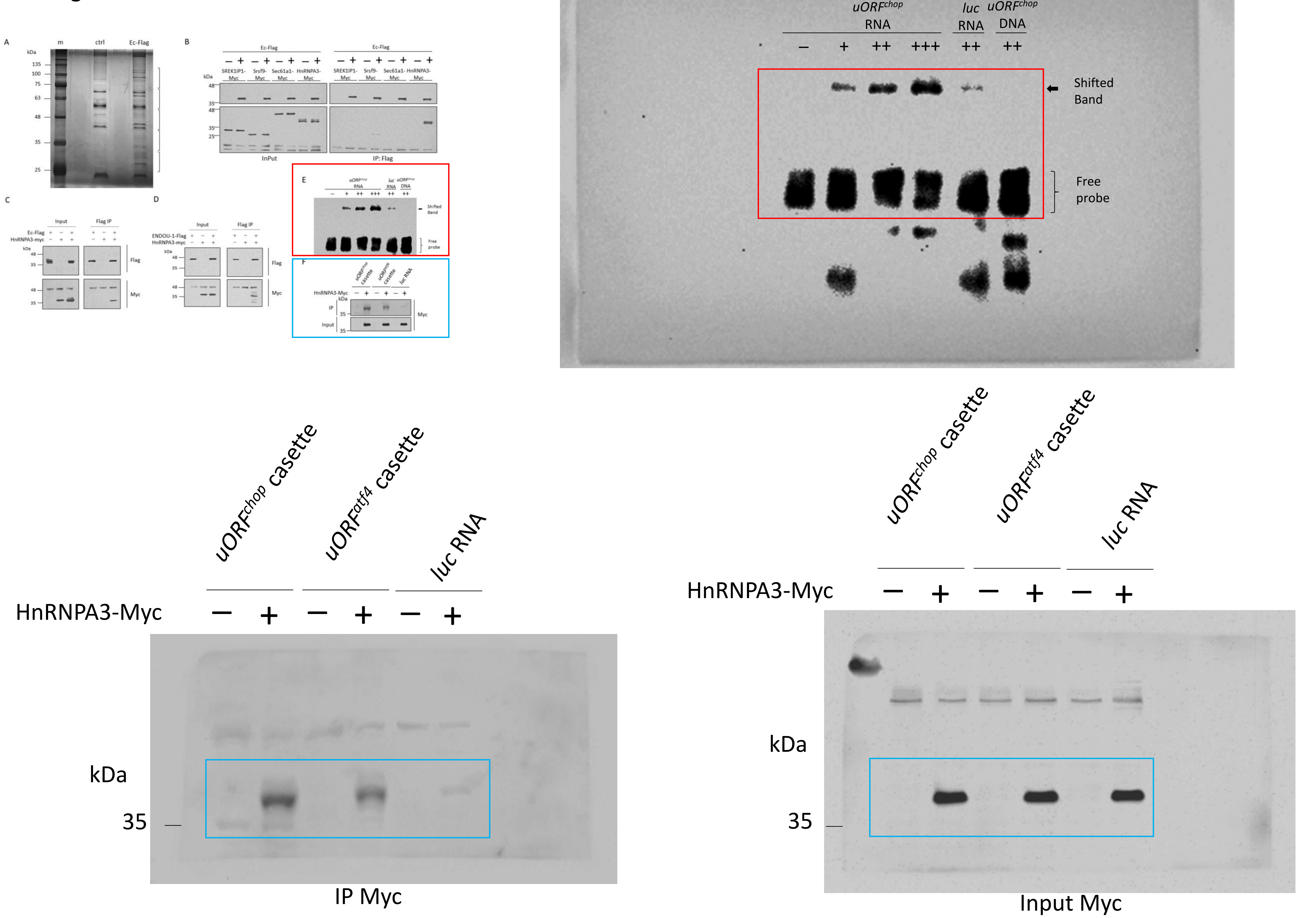
uORF^chop RNA
luc RNA
uORF^chop DNA
– + ++ +++ ++ ++
Shifted Band
Free probe
uORF^chop casette
uORF^atf4 casette
luc RNA
HnRNPA3-Myc
– + – + – +
kDa
35
IP Myc
Input Myc

Figure 2

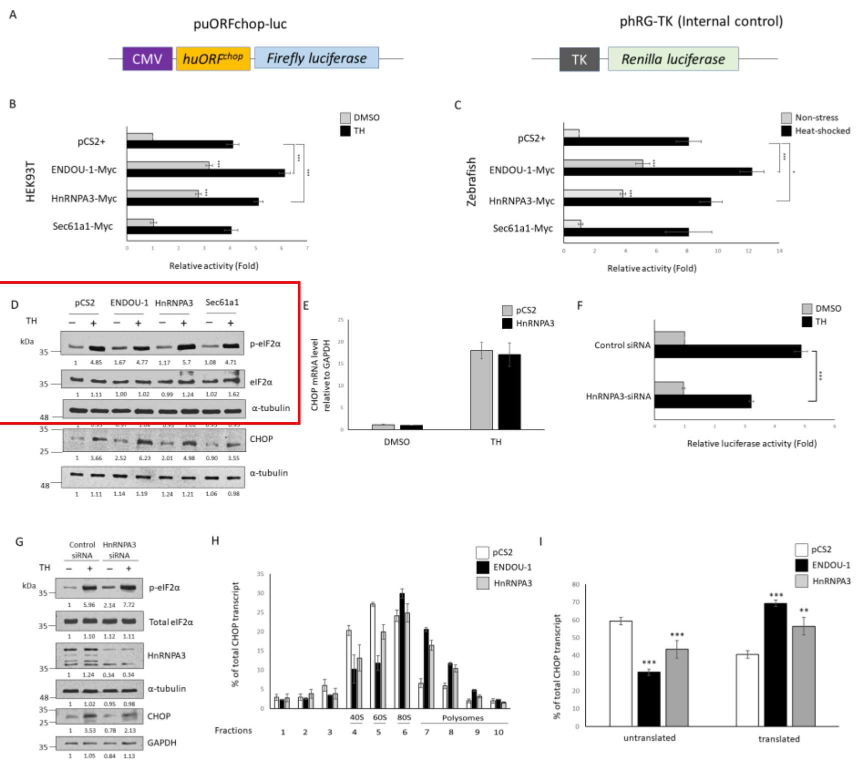

D

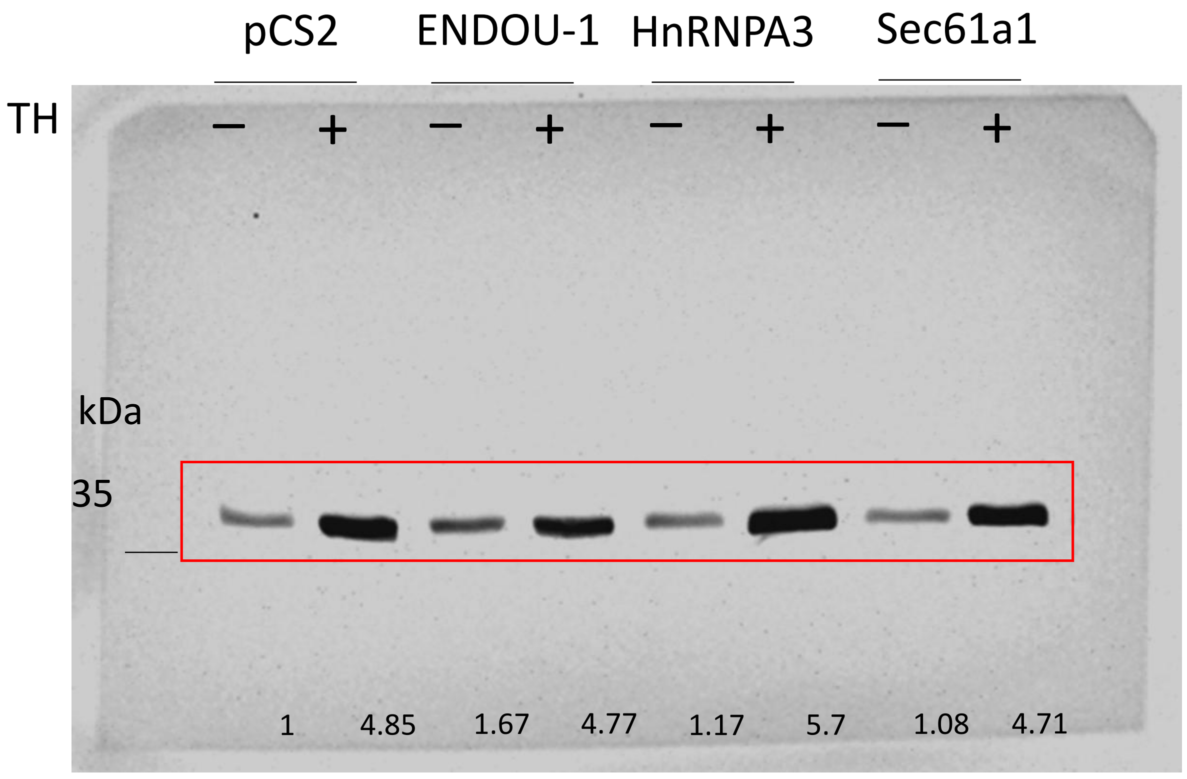

p-eIF2α

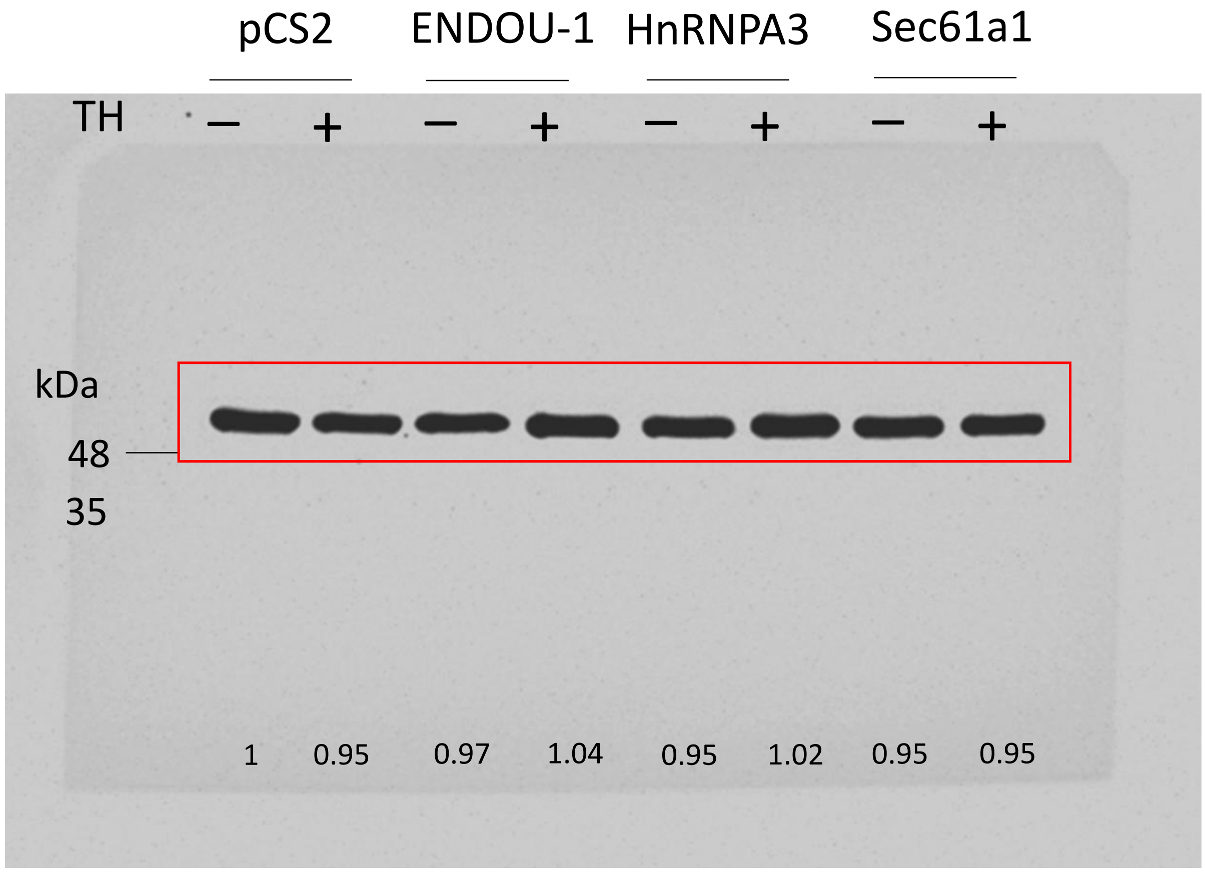

α-tubulin

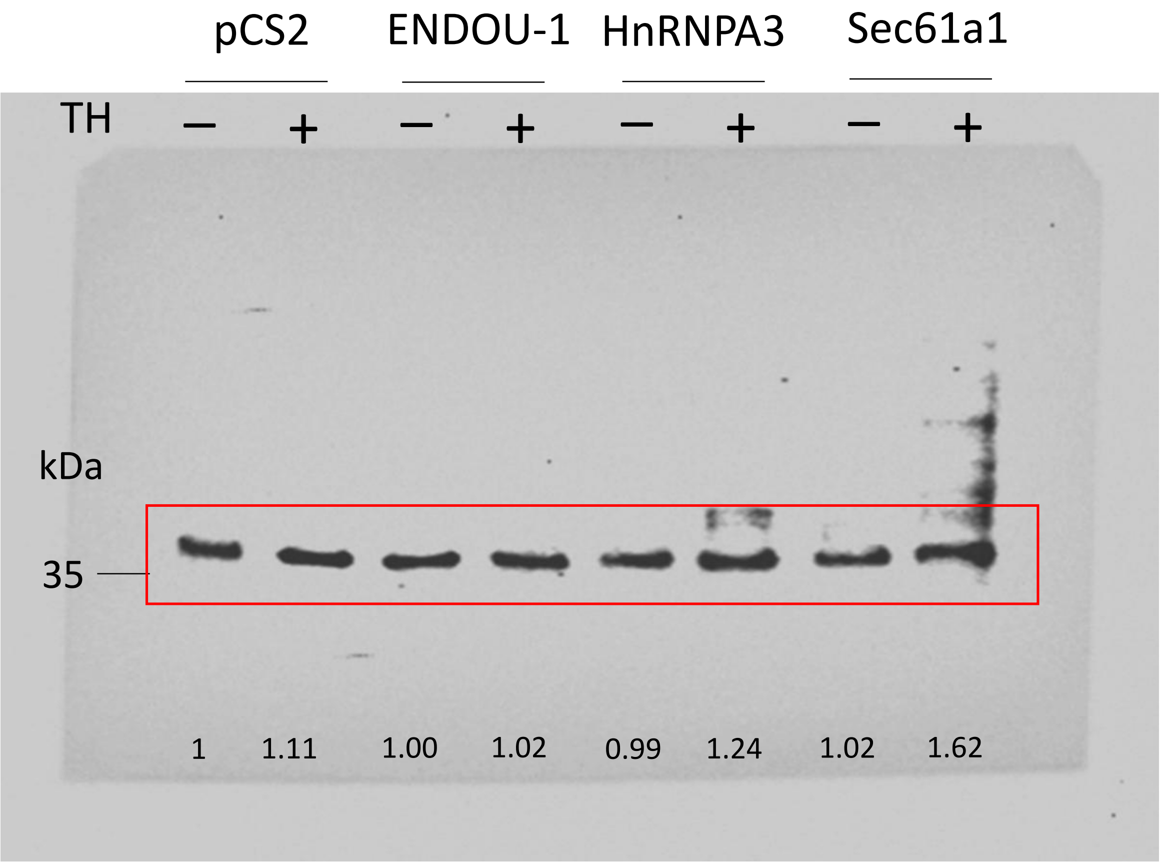

Total eIF2α

Figure 2

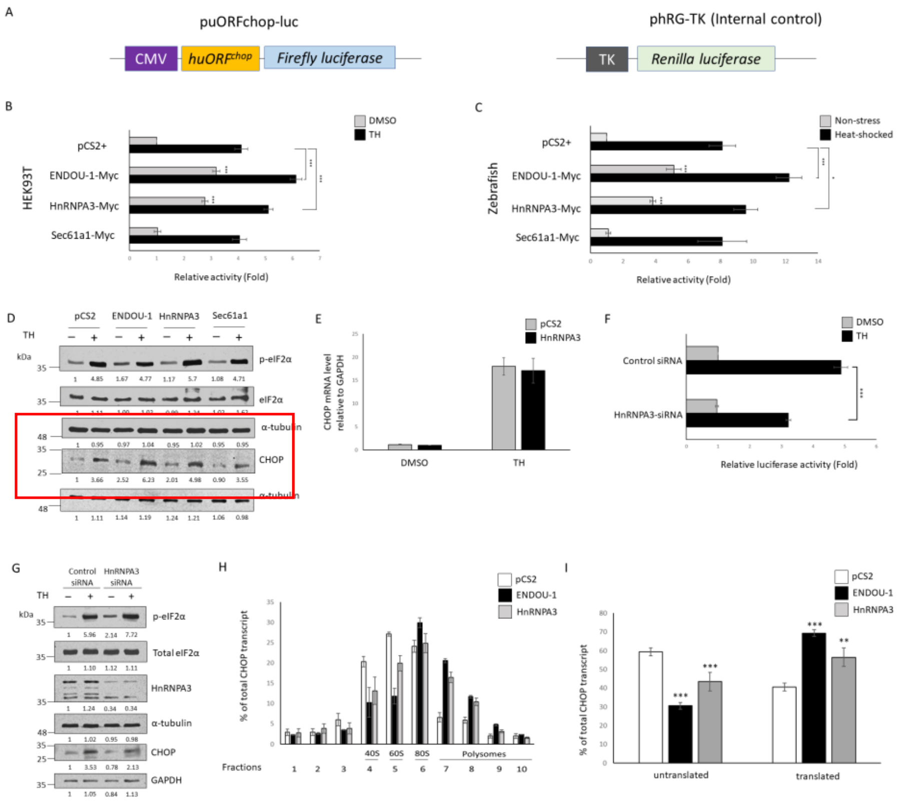

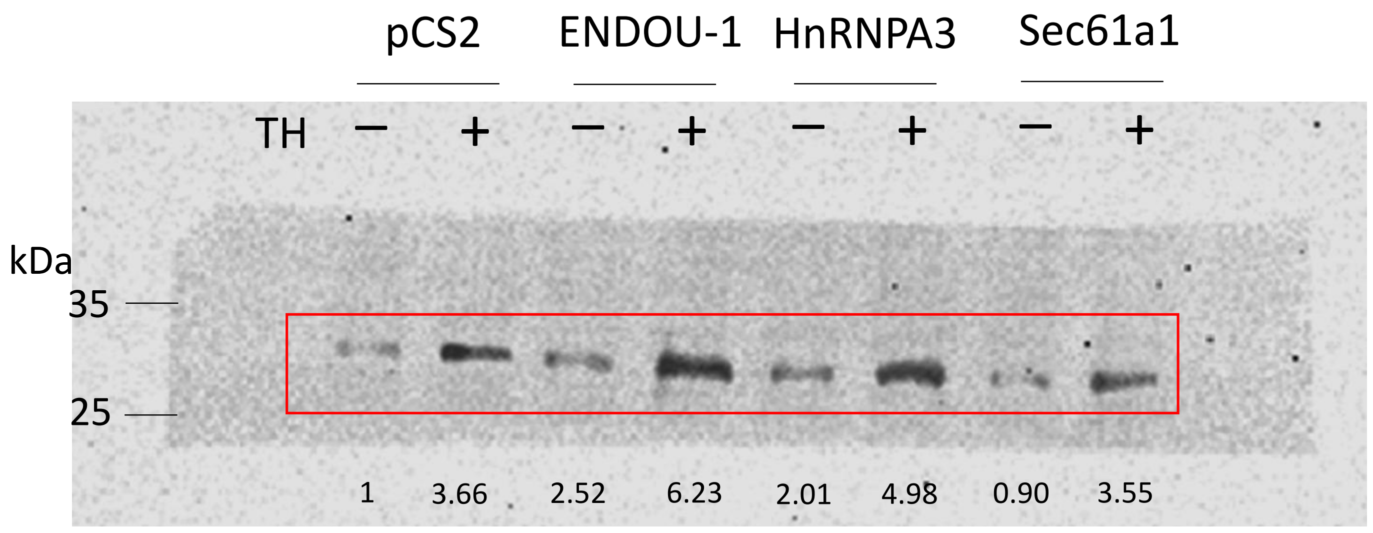

CHOP

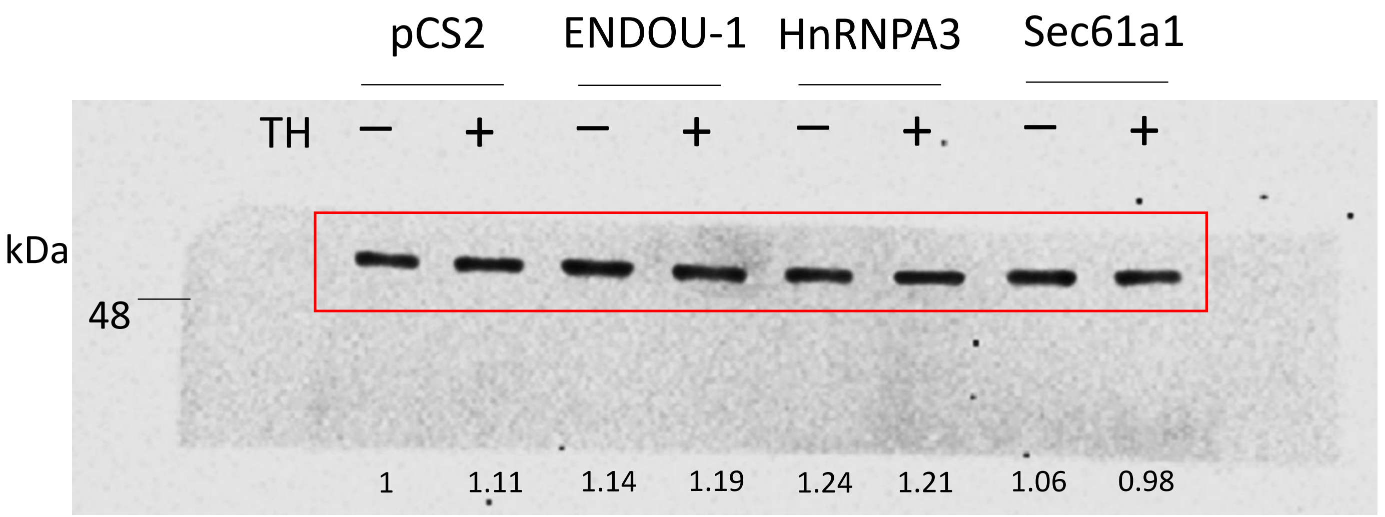

α-tubulin

Figure 2

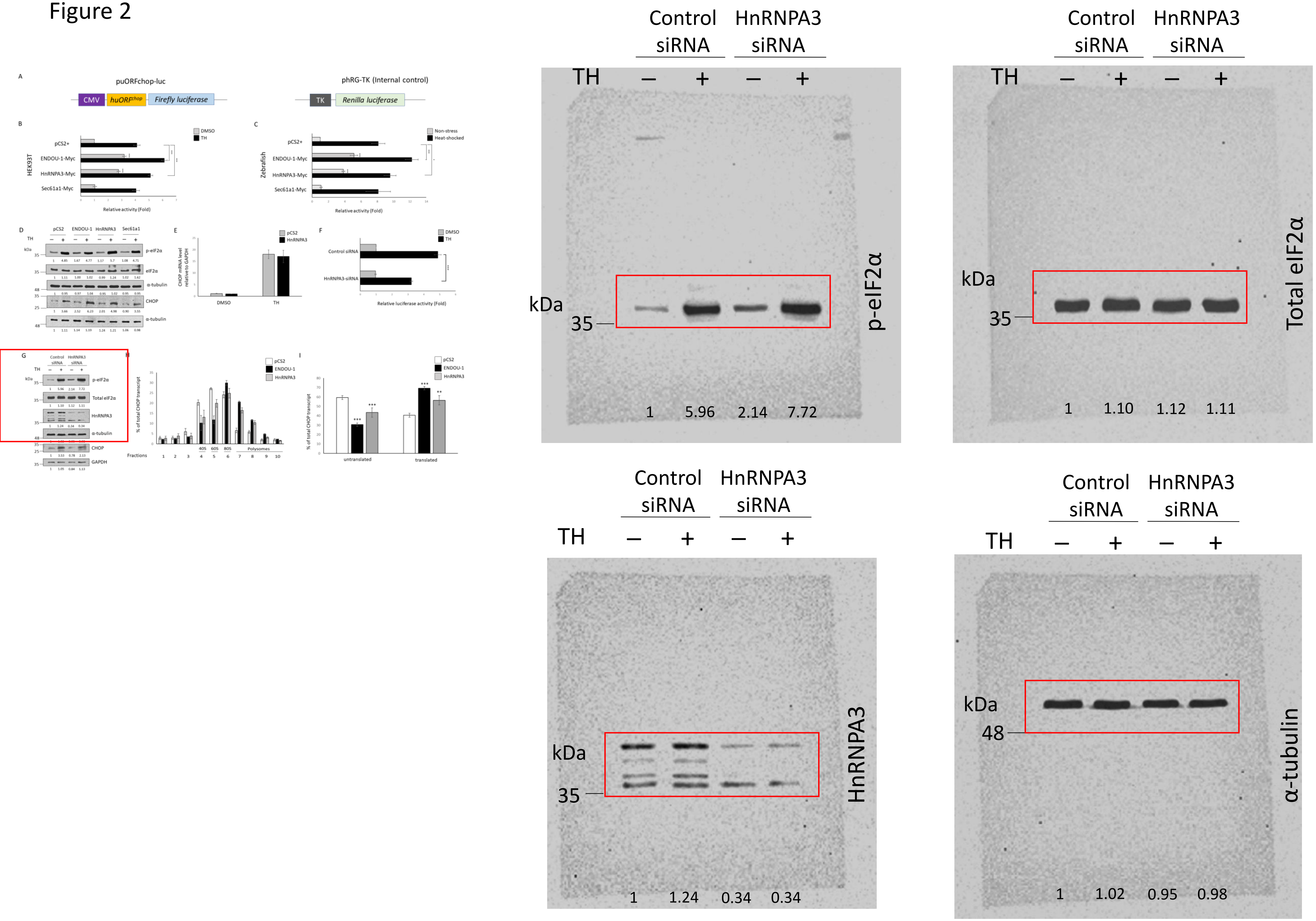

Control siRNA
HnRNPA3 siRNA
TH – + – +
kDa
35
p-eIF2α
1 5.96 2.14 7.72
Control siRNA
HnRNPA3 siRNA
TH – + – +
kDa
35
Total eIF2α
1 1.10 1.12 1.11
Control siRNA
HnRNPA3 siRNA
TH – + – +
kDa
35
HnRNPA3
1 1.24 0.34 0.34
Control siRNA
HnRNPA3 siRNA
TH – + – +
kDa
48
α-tubulin
1 1.02 0.95 0.98

Figure 2

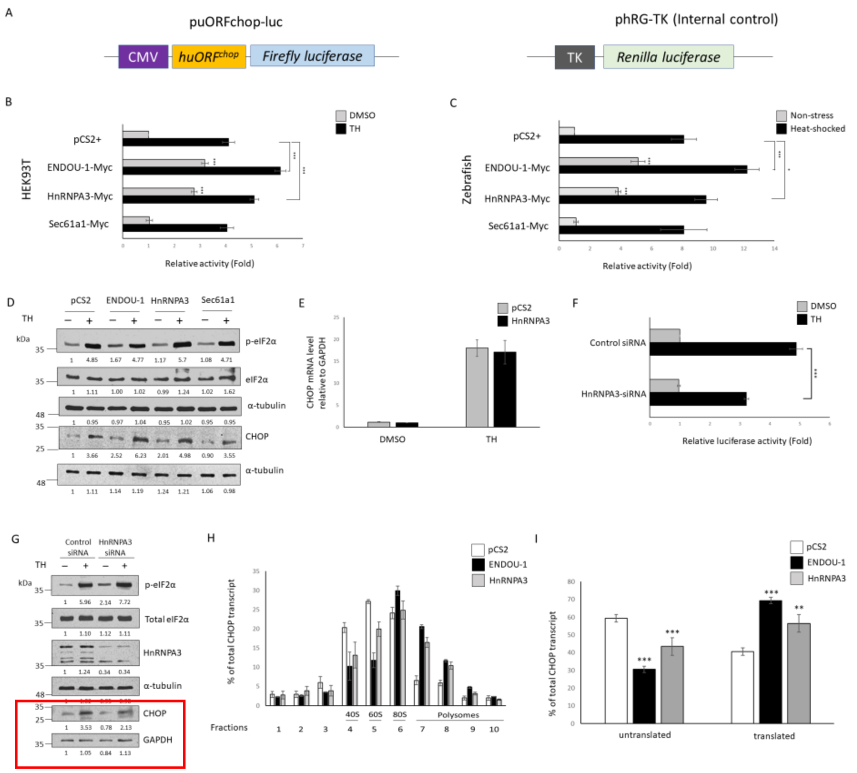

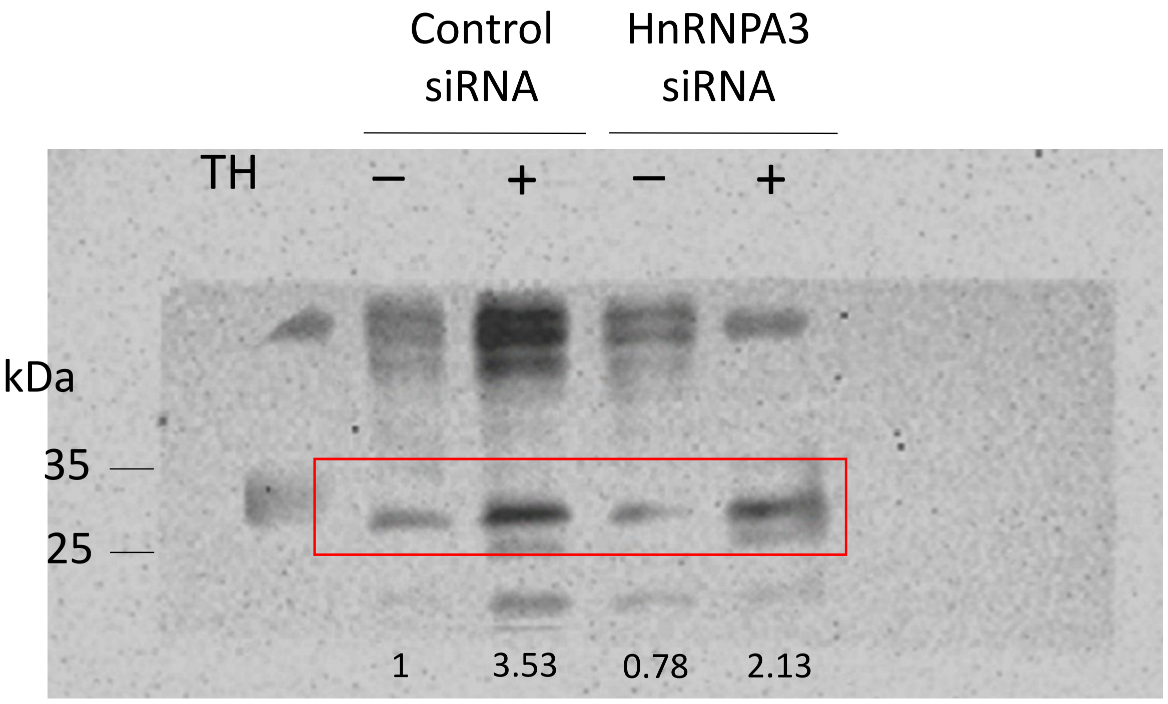

CHOP

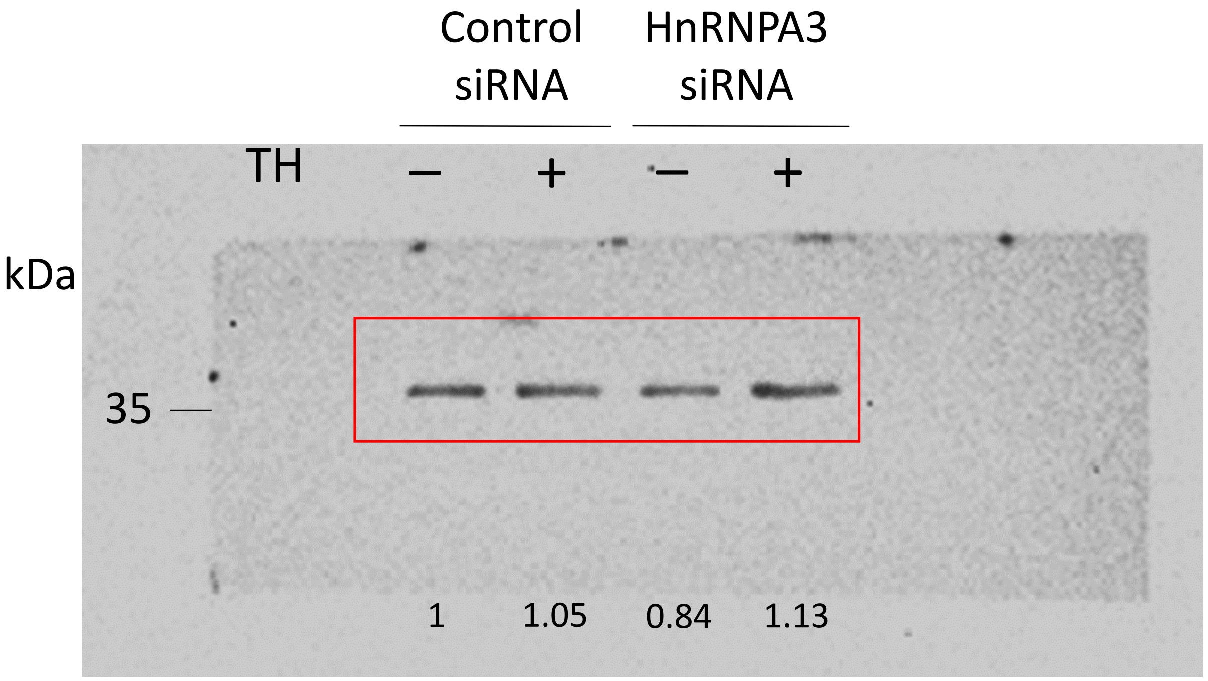

GAPDH

Figure 3

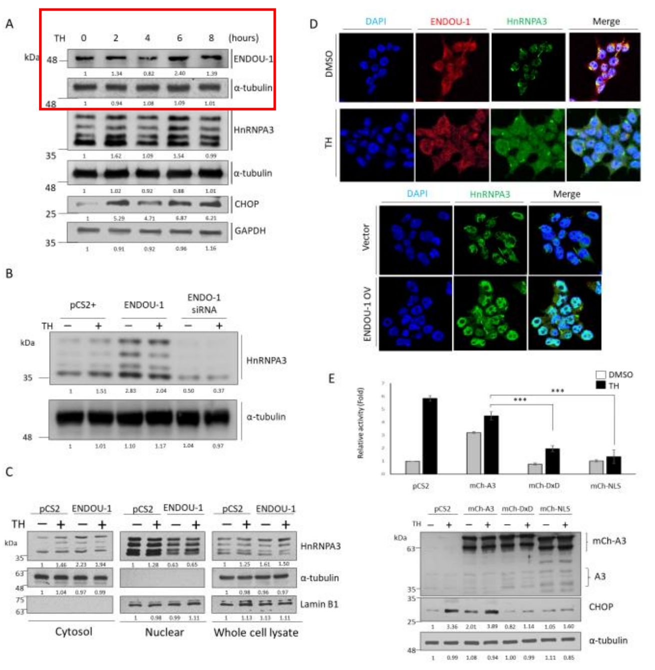

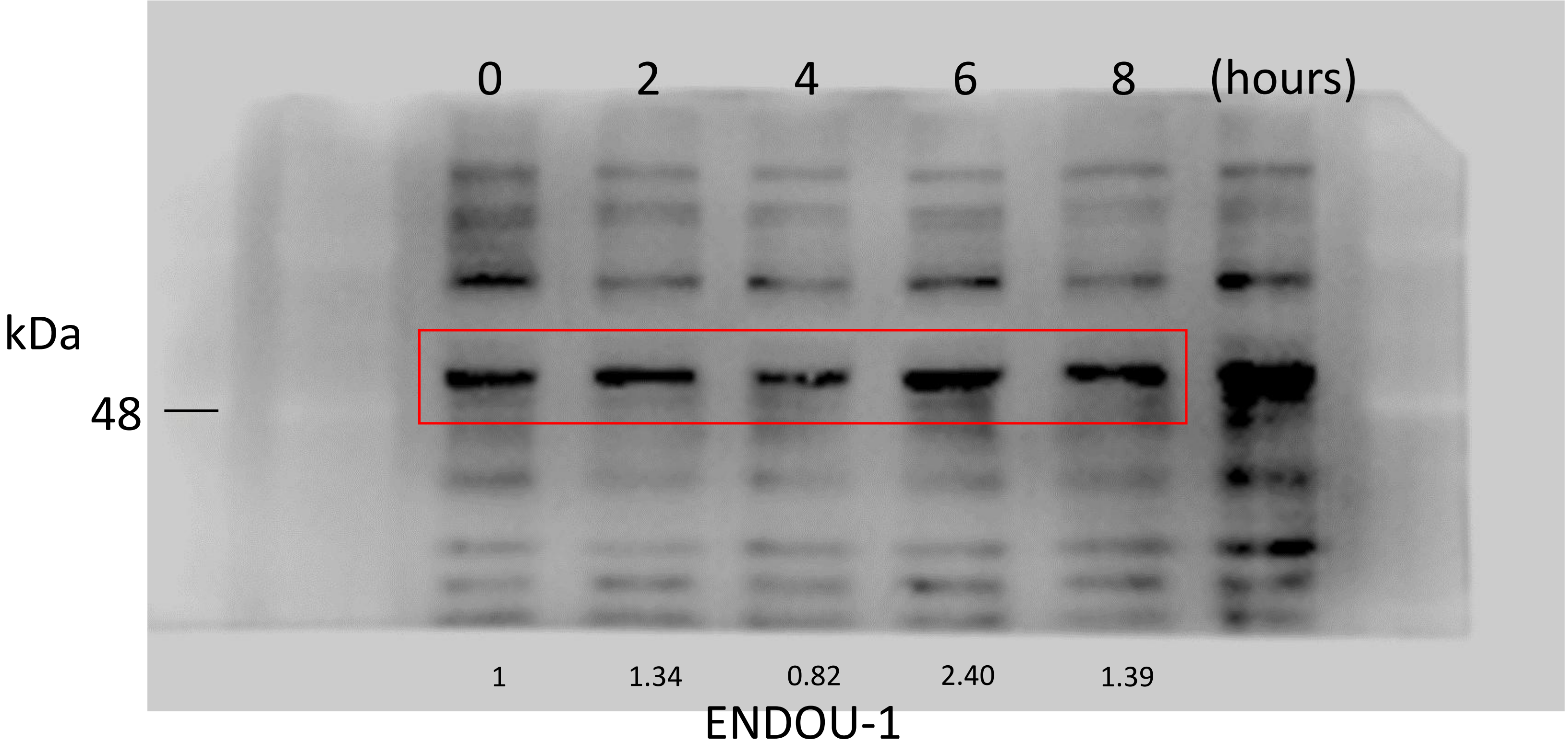

ENDOU-1

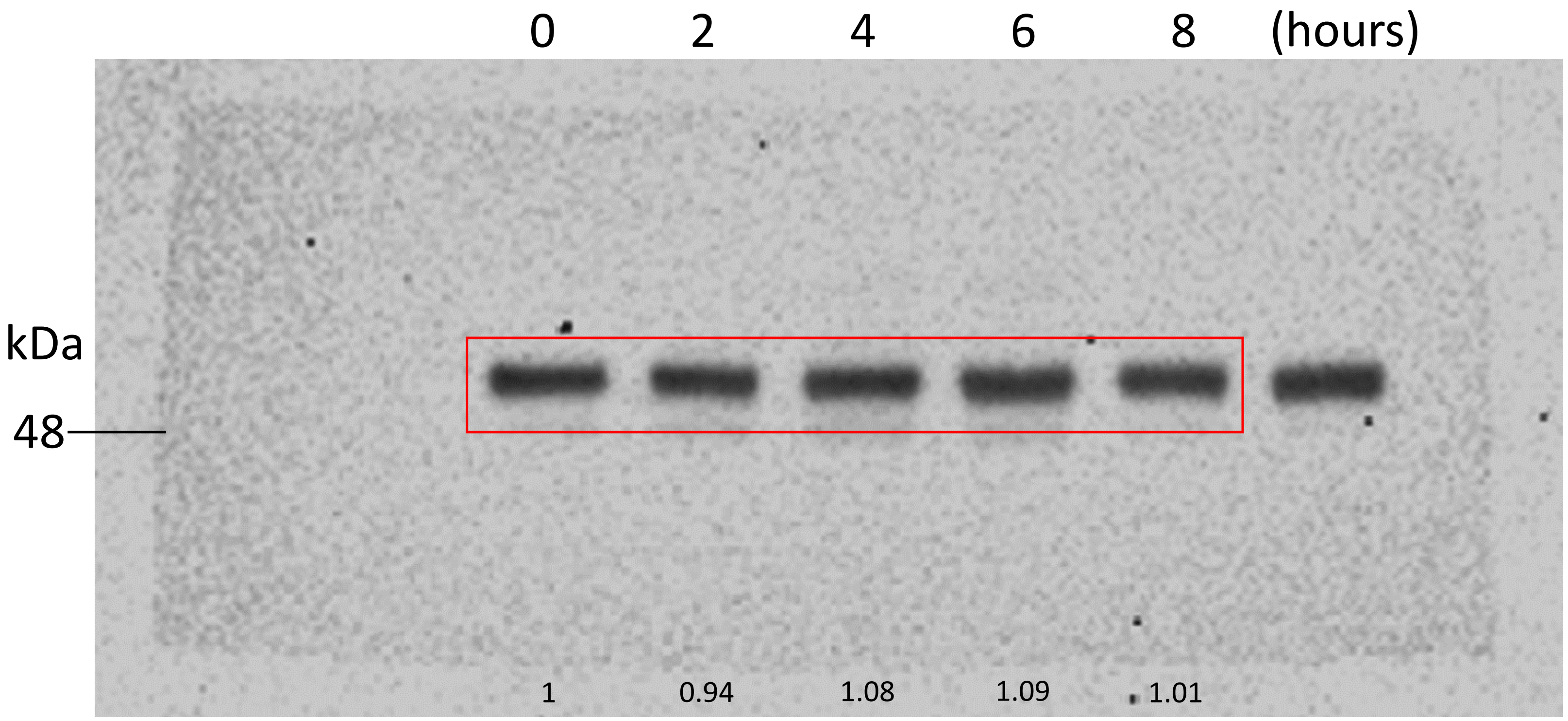

α-tubulin

Figure 3

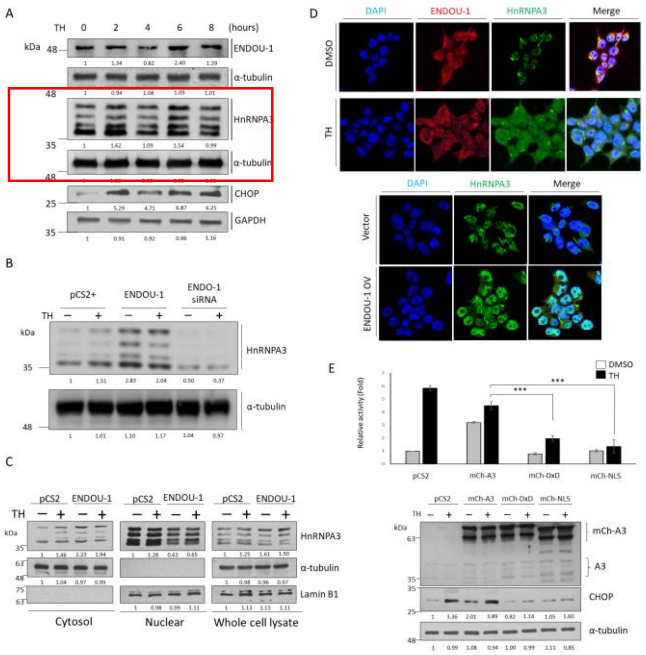

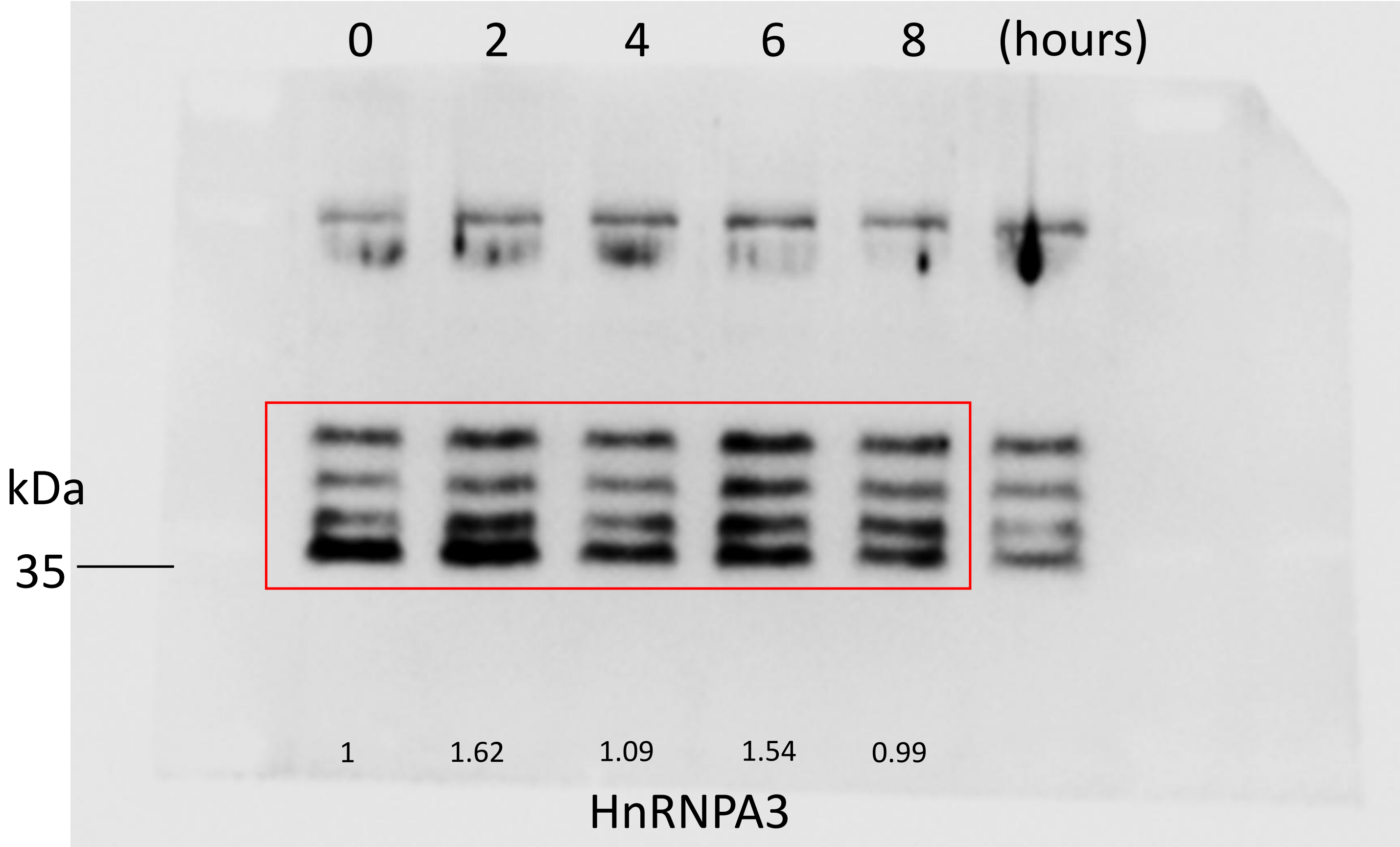
0 2 4 6 8 (hours)
kDa
35
1 1.62 1.09 1.54 0.99
HnRNPA3

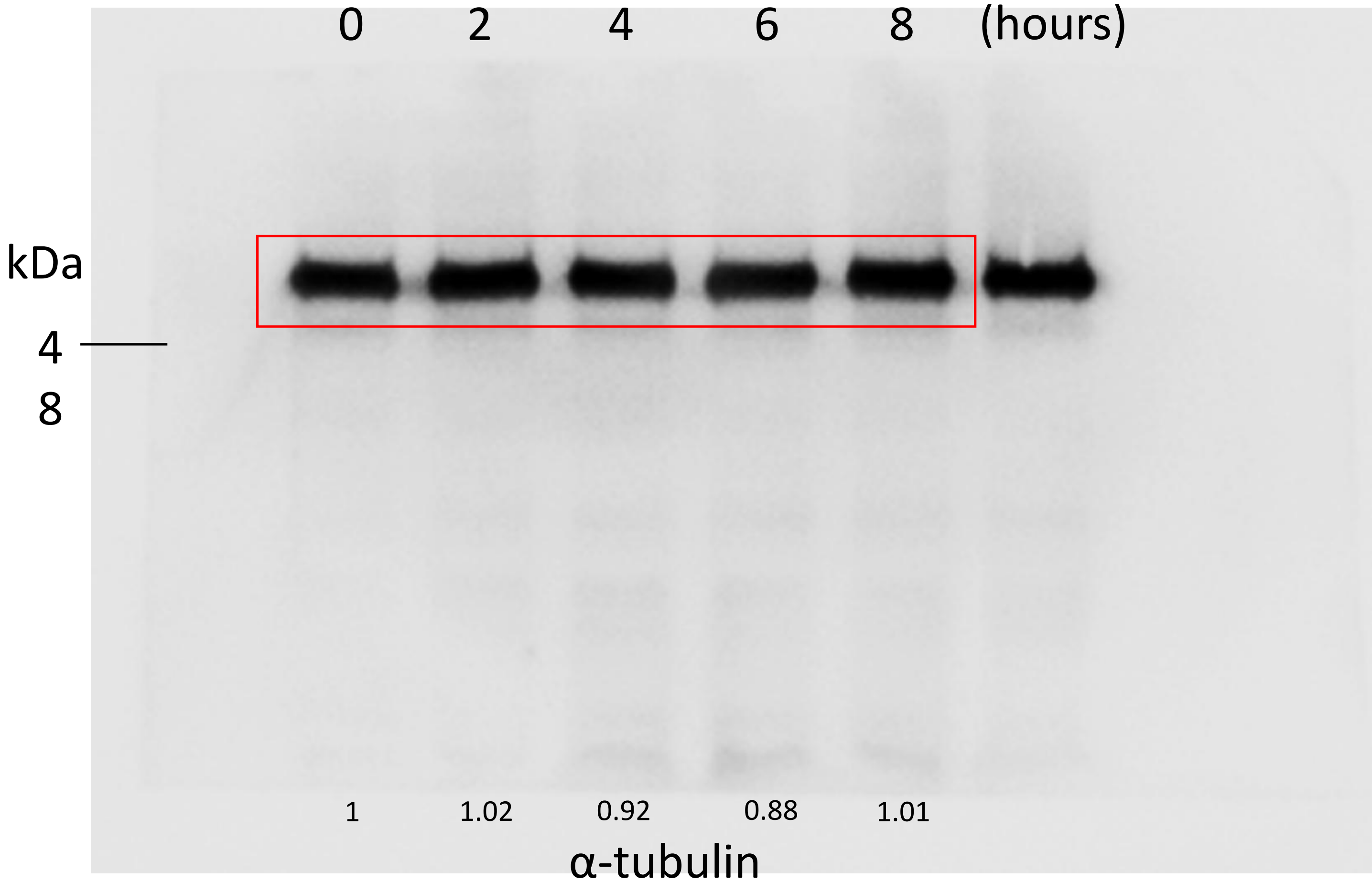
0 2 4 6 8 (hours)
kDa
4
8
1 1.02 0.92 0.88 1.01
α-tubulin

Figure 3

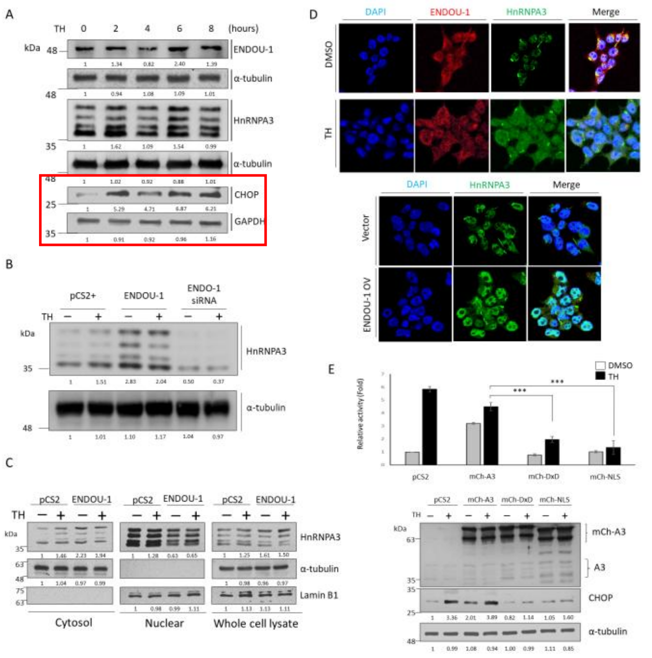

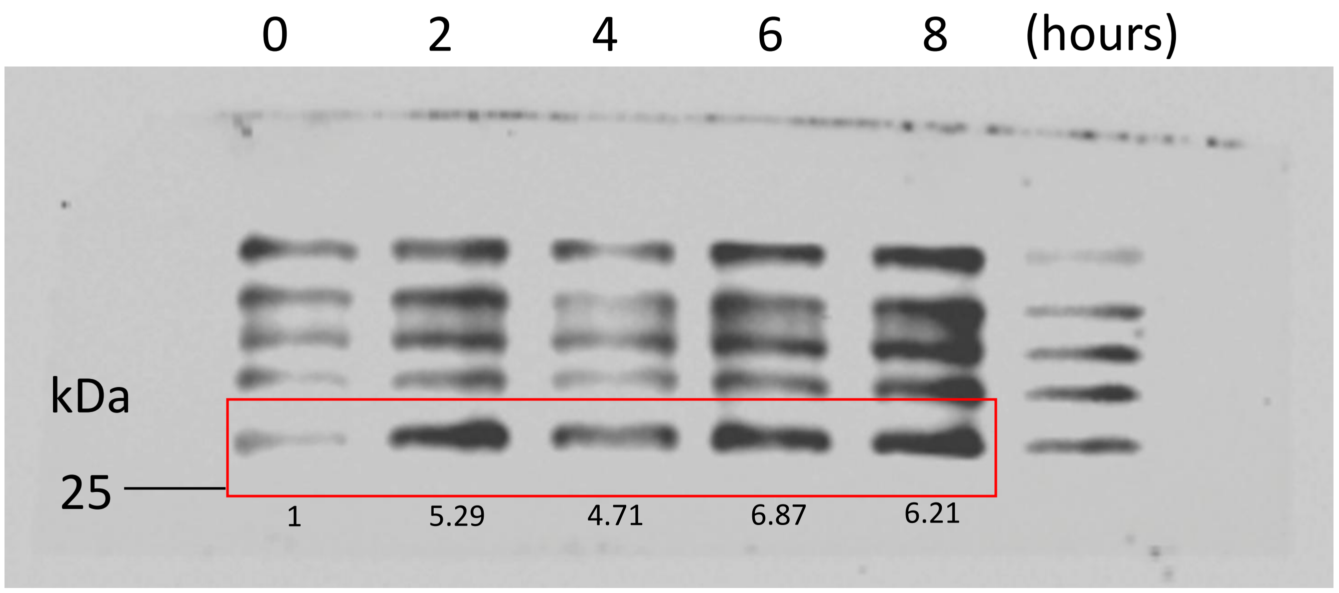

CHOP

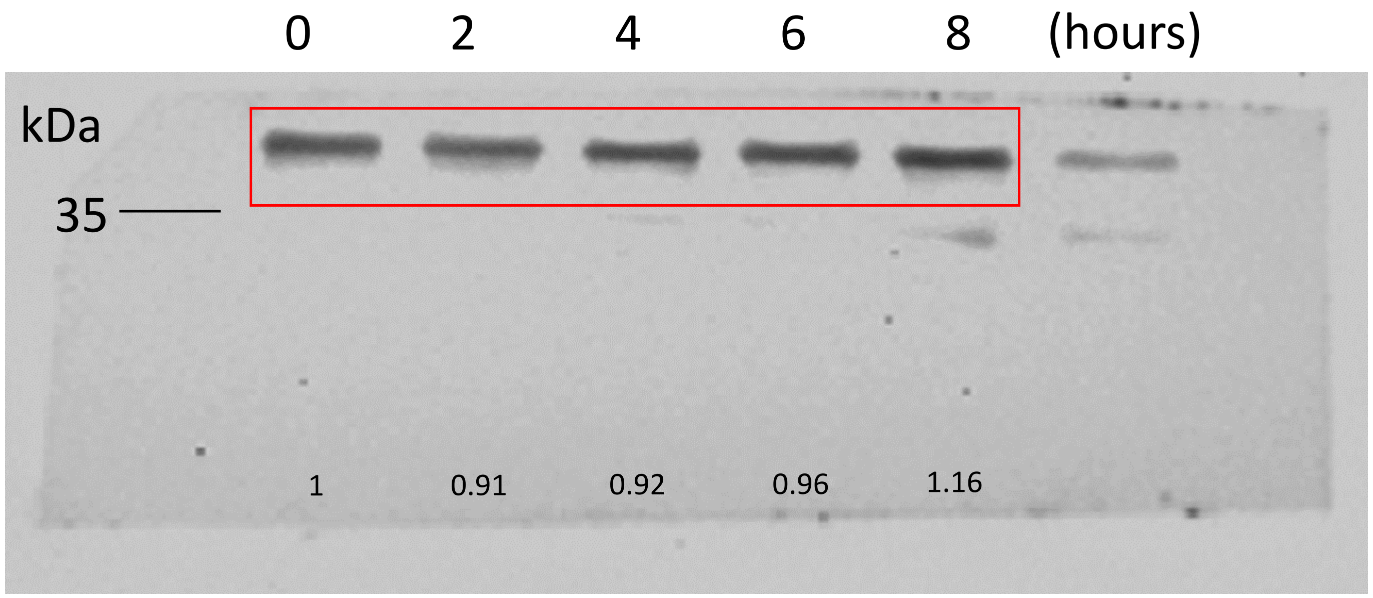

GAPDH

Figure 3

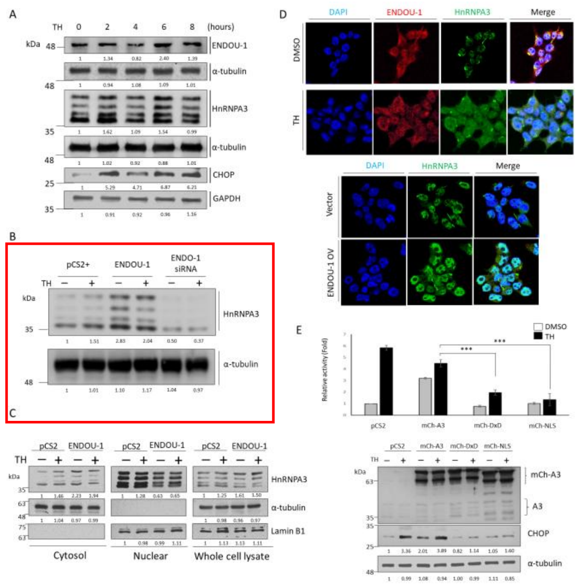

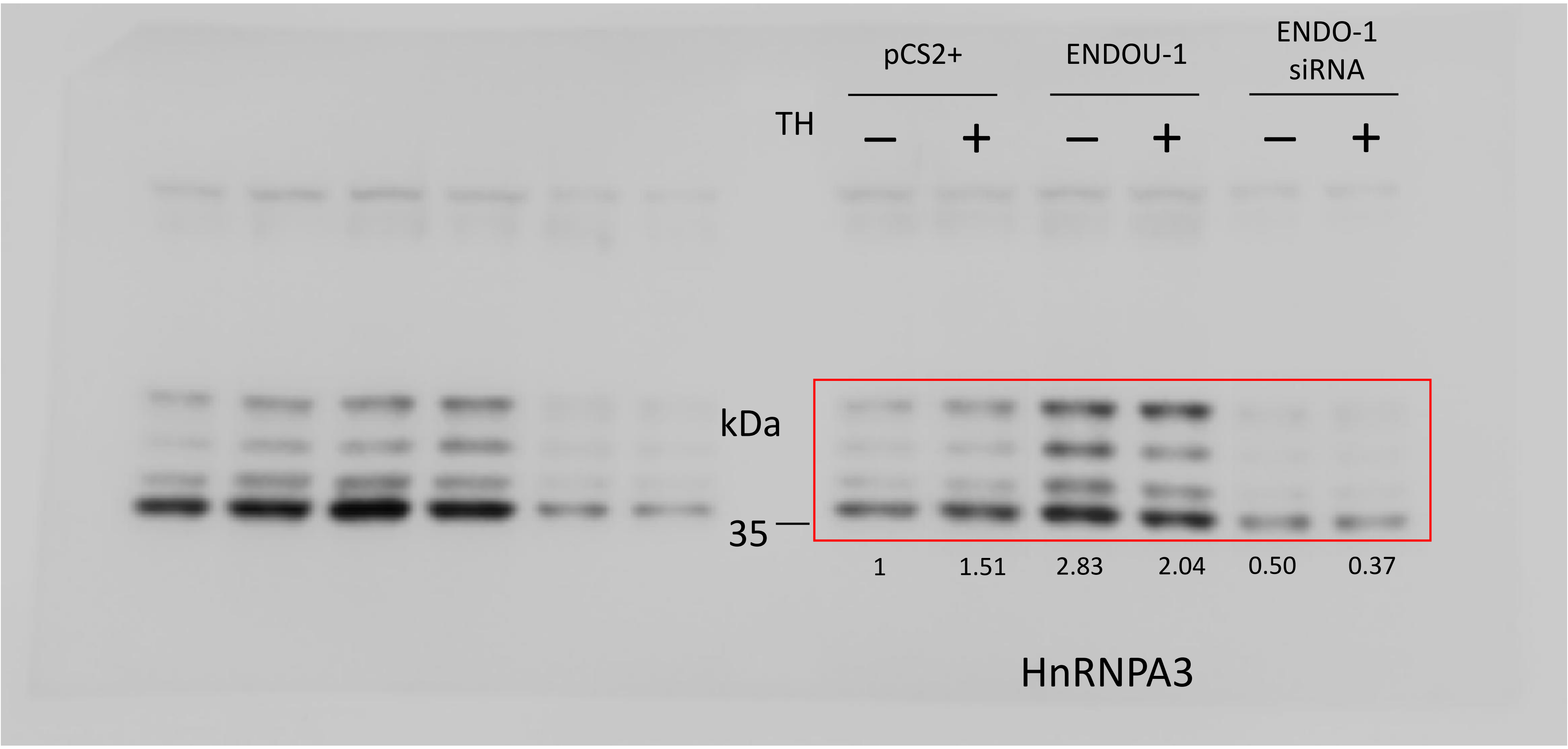
pCS2+
ENDOU-1
ENDO-1 siRNA
TH
– + – + – +
kDa
35
1 1.51 2.83 2.04 0.50 0.37
HnRNPA3

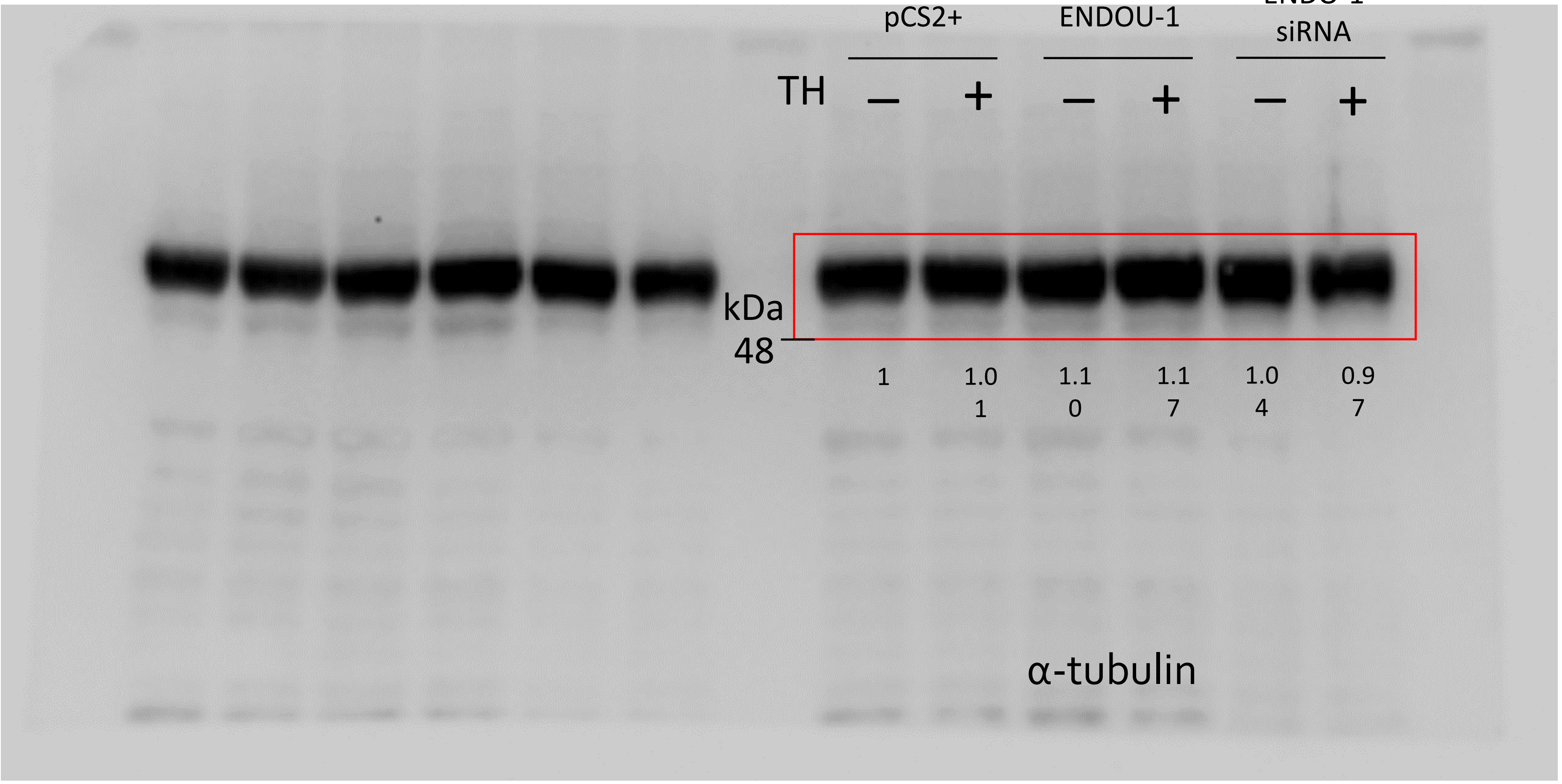
pCS2+
ENDOU-1
ENDO-1 siRNA
TH
– + – + – +
kDa
48
1 1.01 1.10 1.17 1.04 0.97
α-tubulin

Figure 3

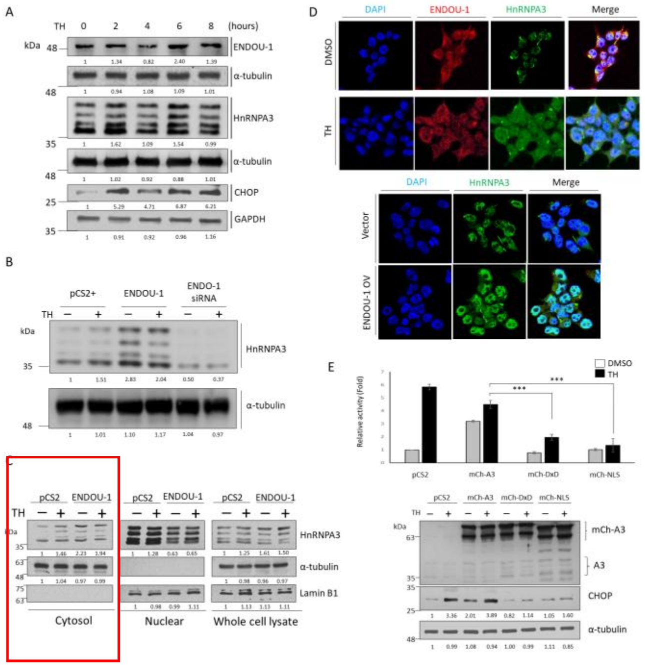

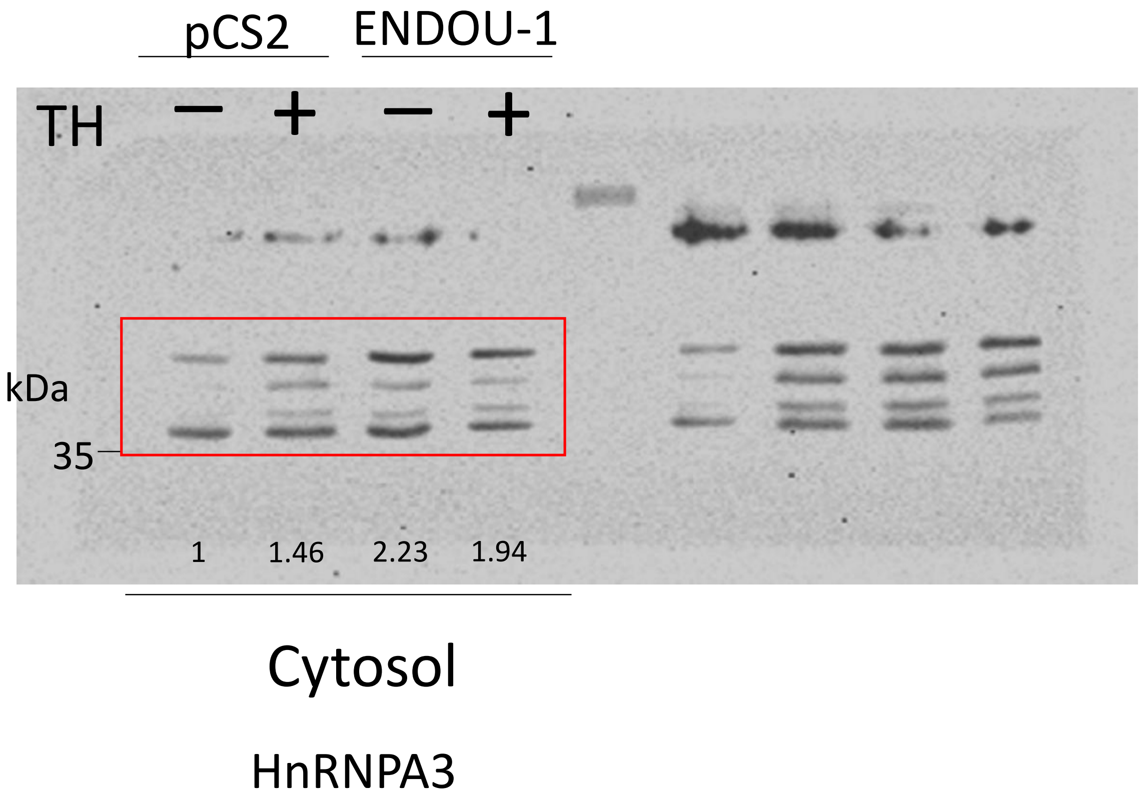
pCS2
ENDOU-1
TH − + − +
kDa
35
1 1.46 2.23 1.94
Cytosol
HnRNPA3

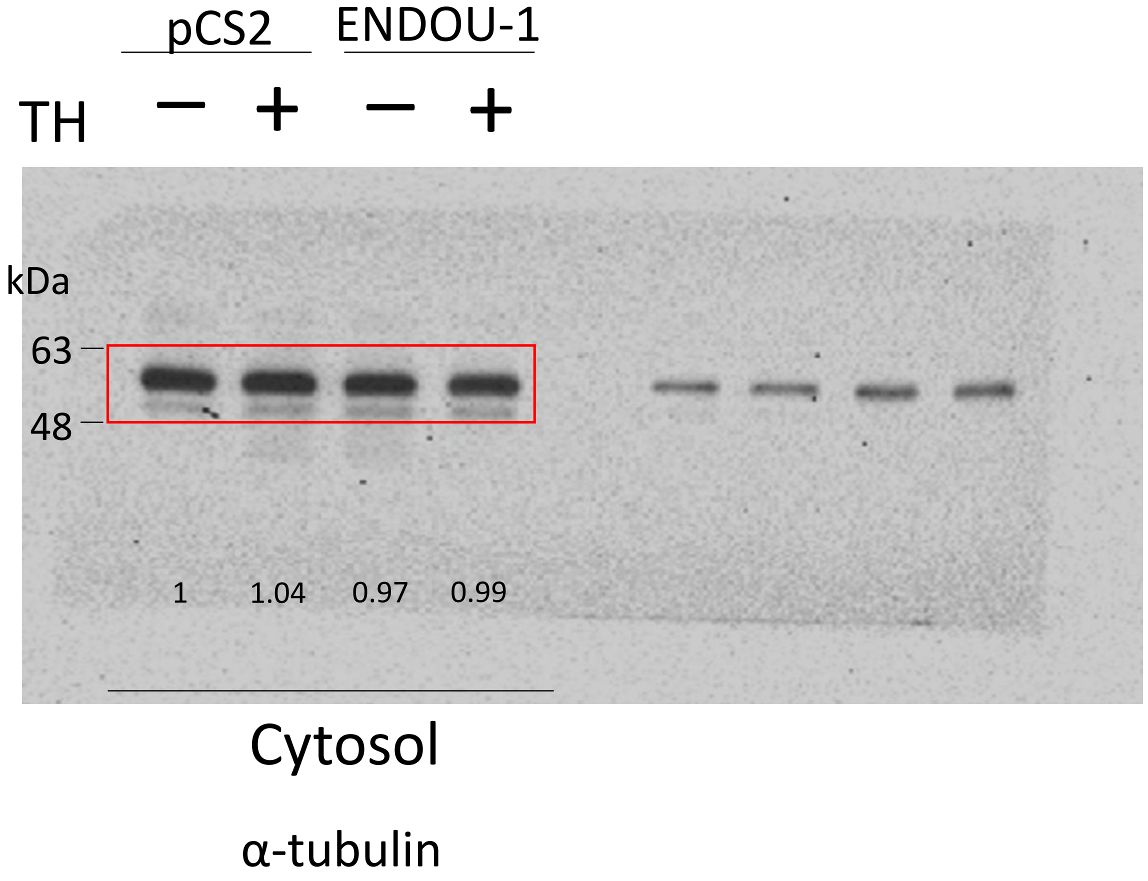
pCS2
ENDOU-1
TH − + − +
kDa
63
48
1 1.04 0.97 0.99
Cytosol
α-tubulin

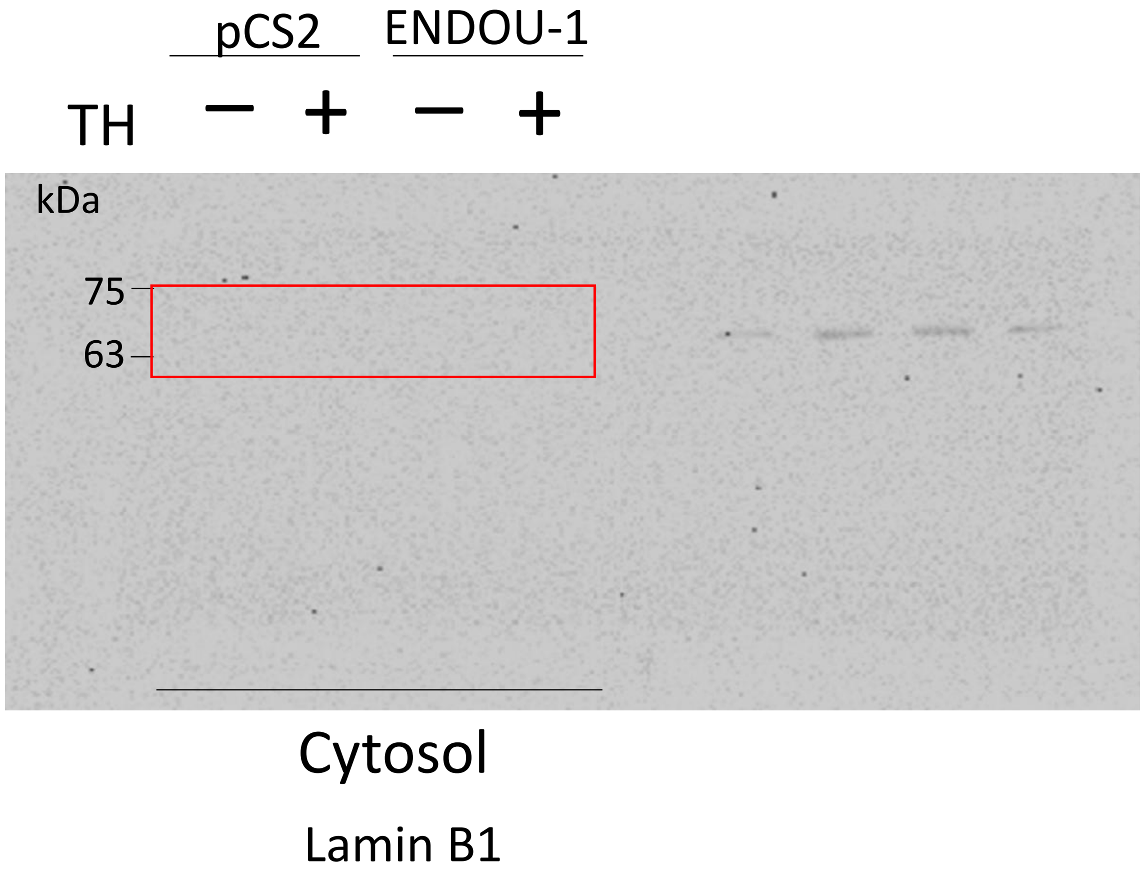
pCS2
ENDOU-1
TH − + − +
kDa
75
63
Cytosol
Lamin B1

Figure 3

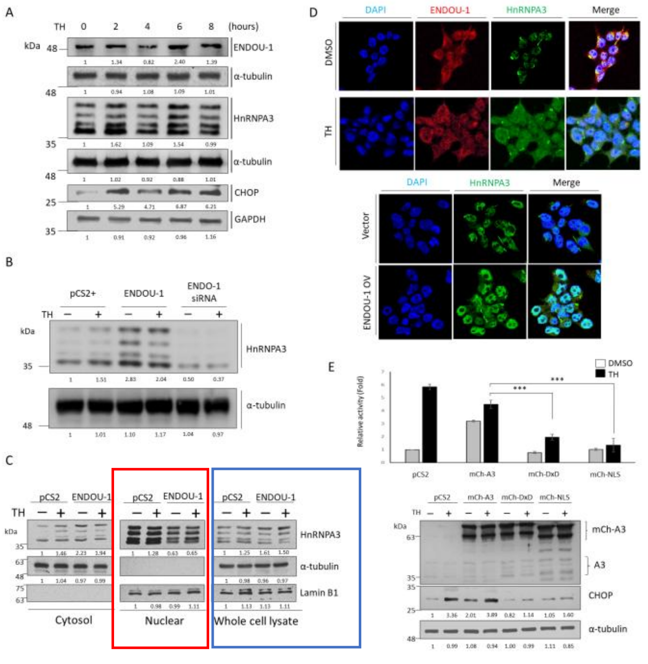

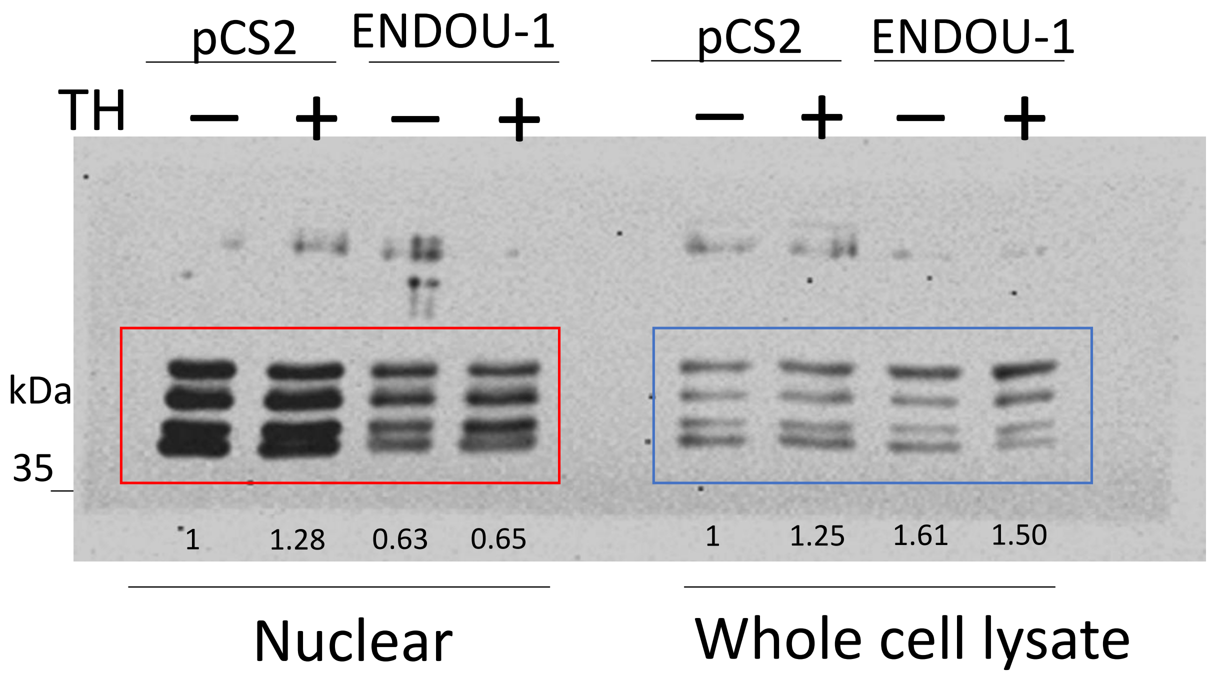

HnRNPA3

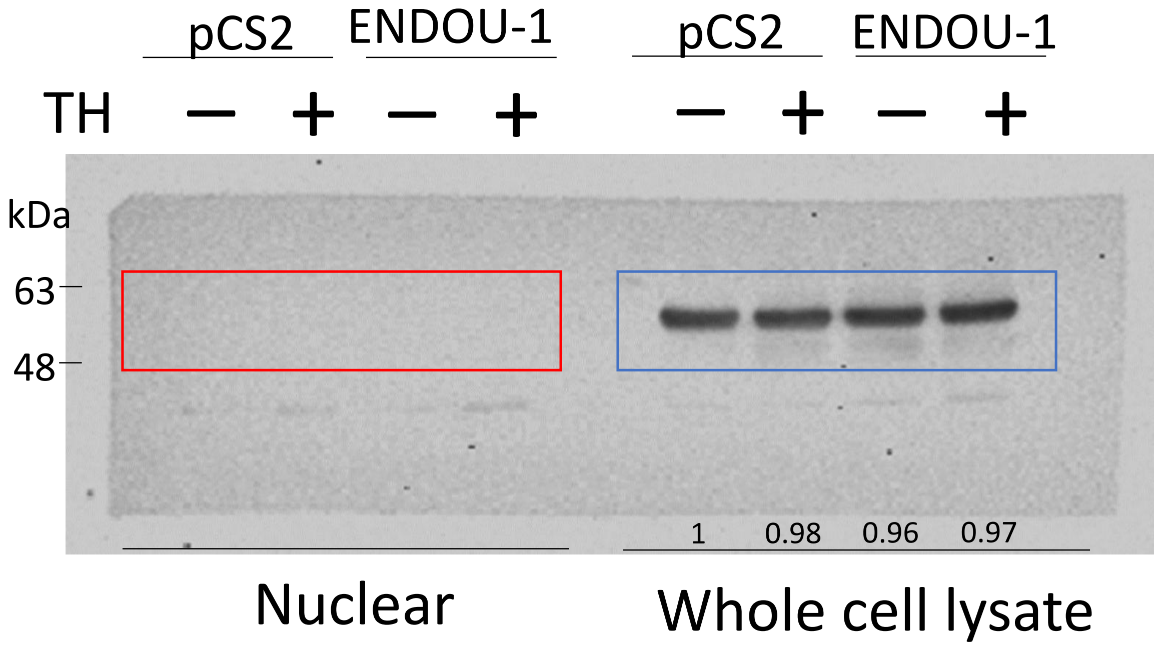

α-tubulin

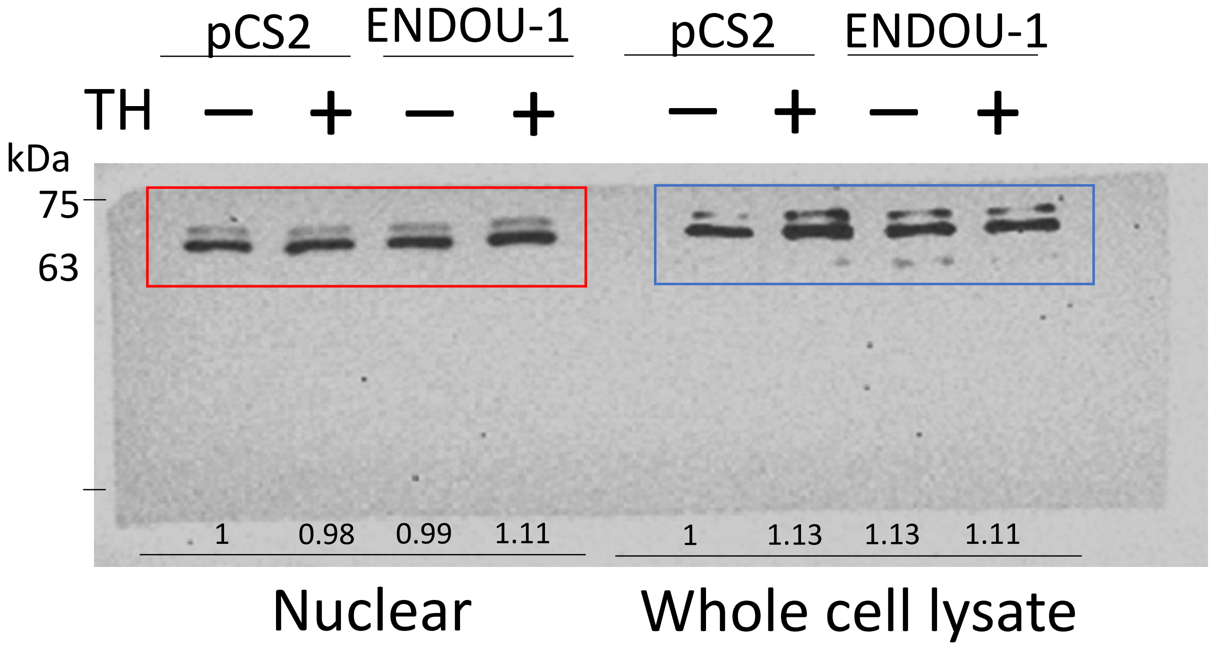

Lamin B1

Figure 3

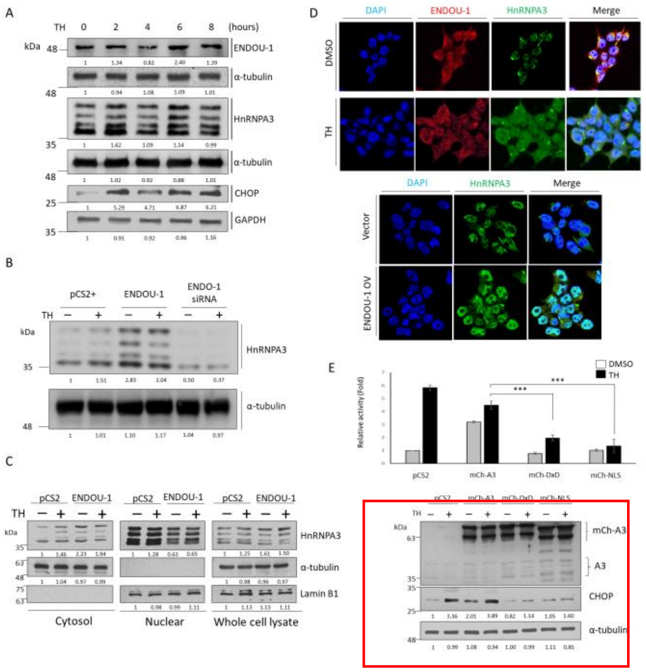

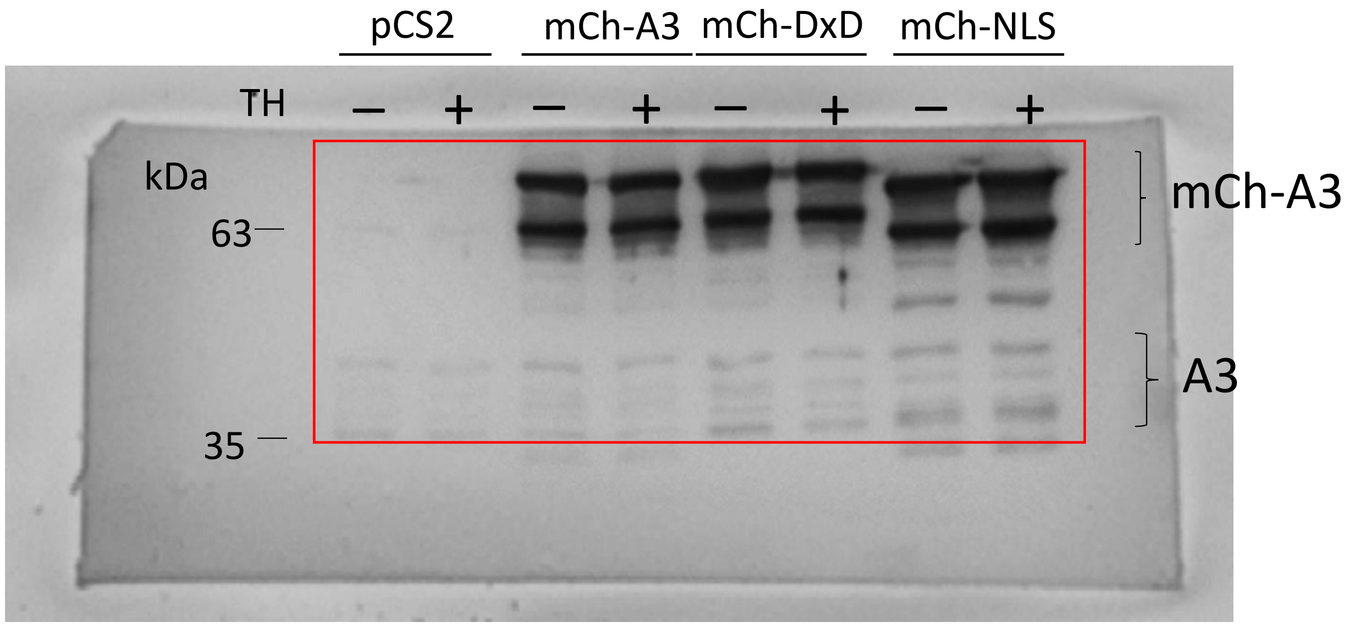

HnRNPA3

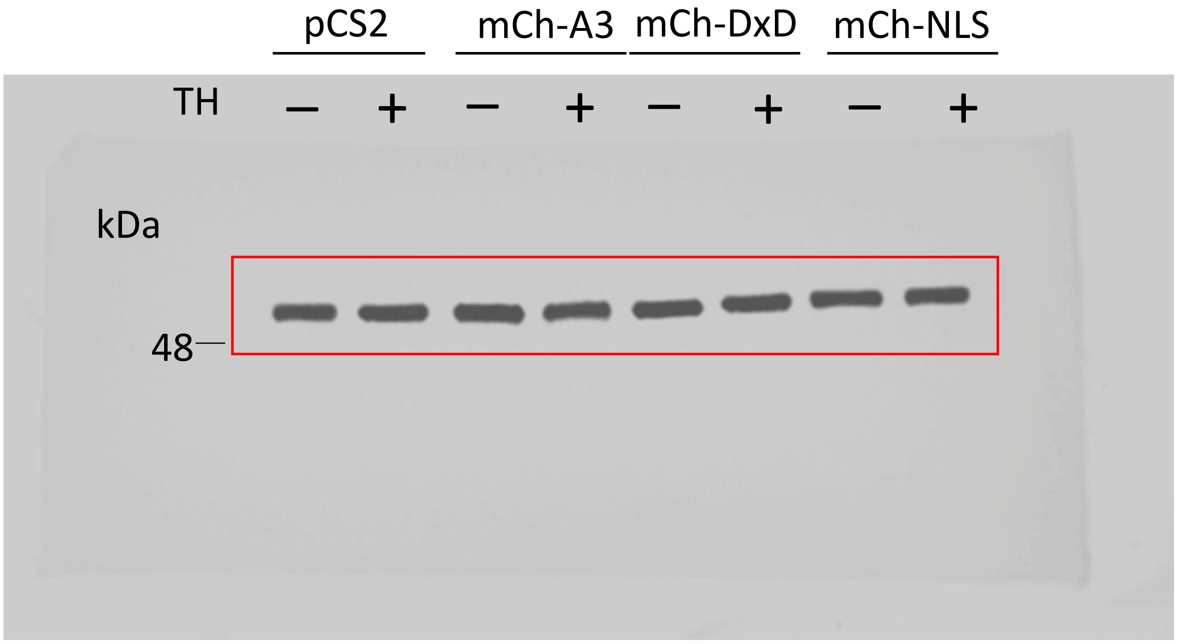

α-tubulin

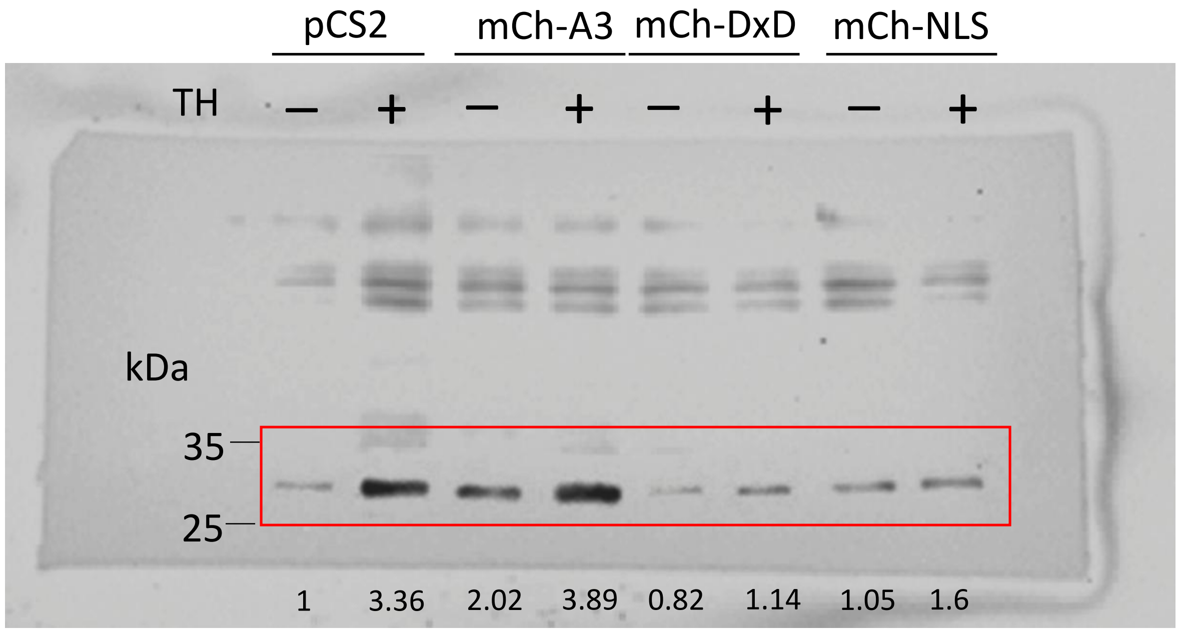

CHOP

Figure 3

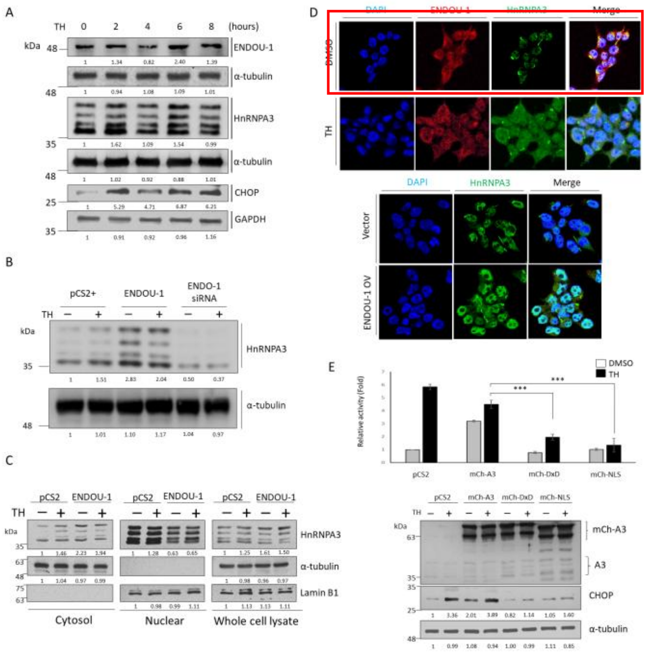

A
B
C
D
E
ENDOU-1
α-tubulin
HnRNPA3
CHOP
GAPDH
Lamin B1
Cytosol
Nuclear
Whole cell lysate

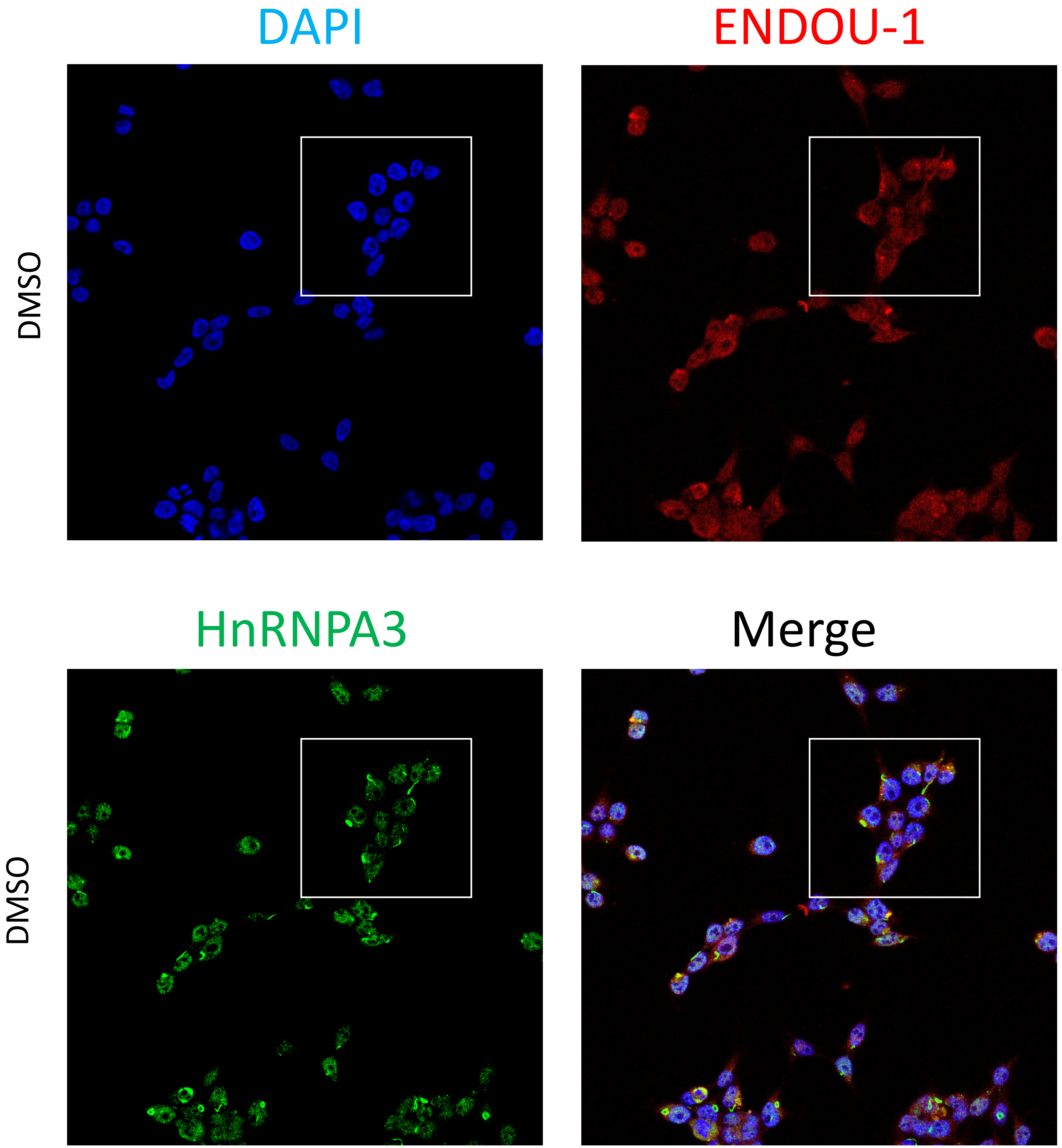

DAPI
ENDOU-1
DMSO
HnRNPA3
Merge
DMSO

Figure 3

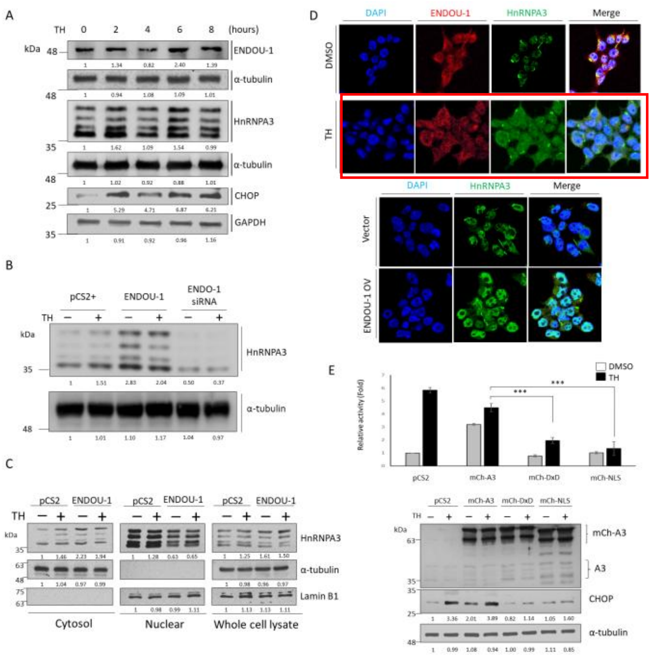

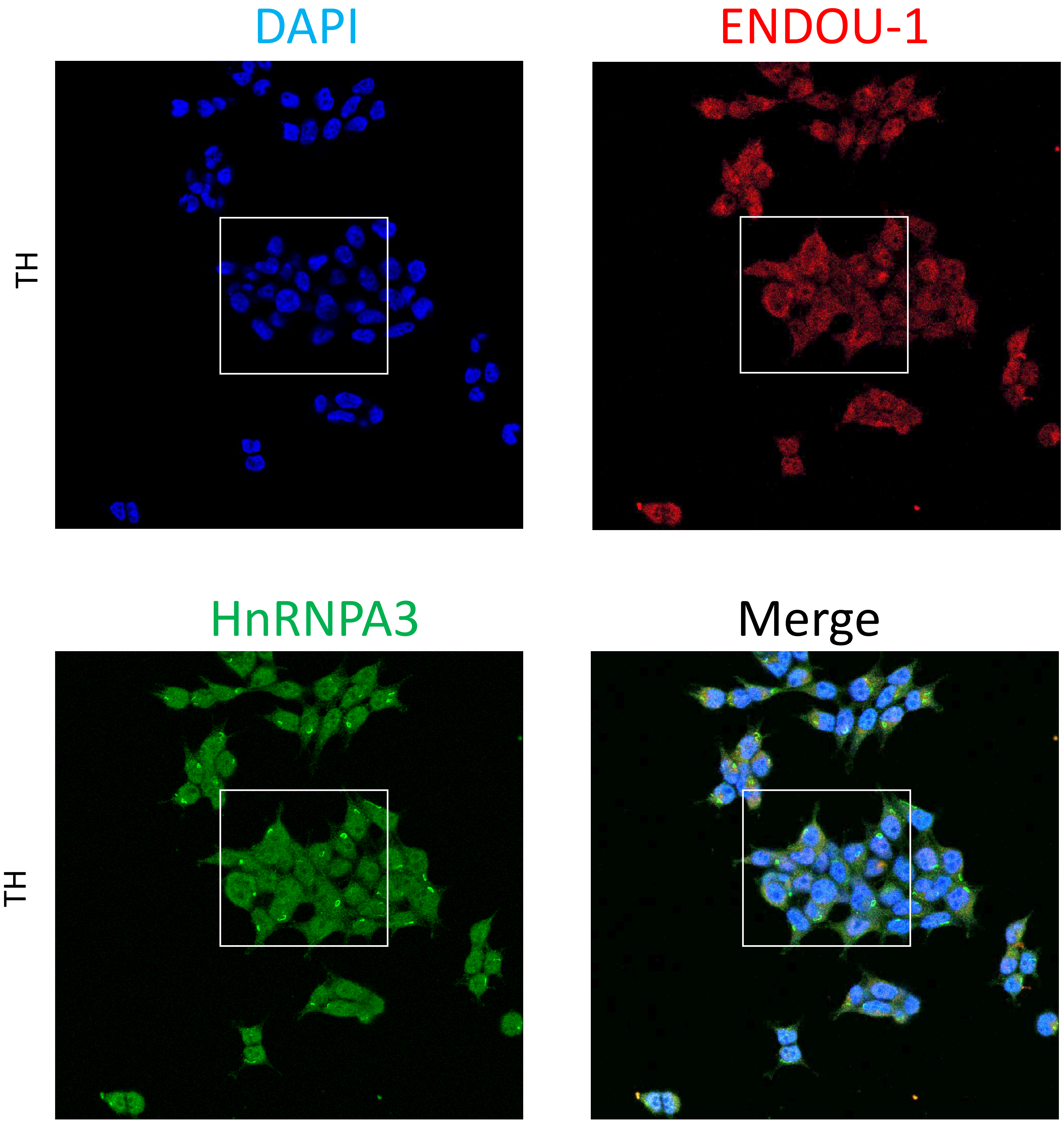

Figure 3

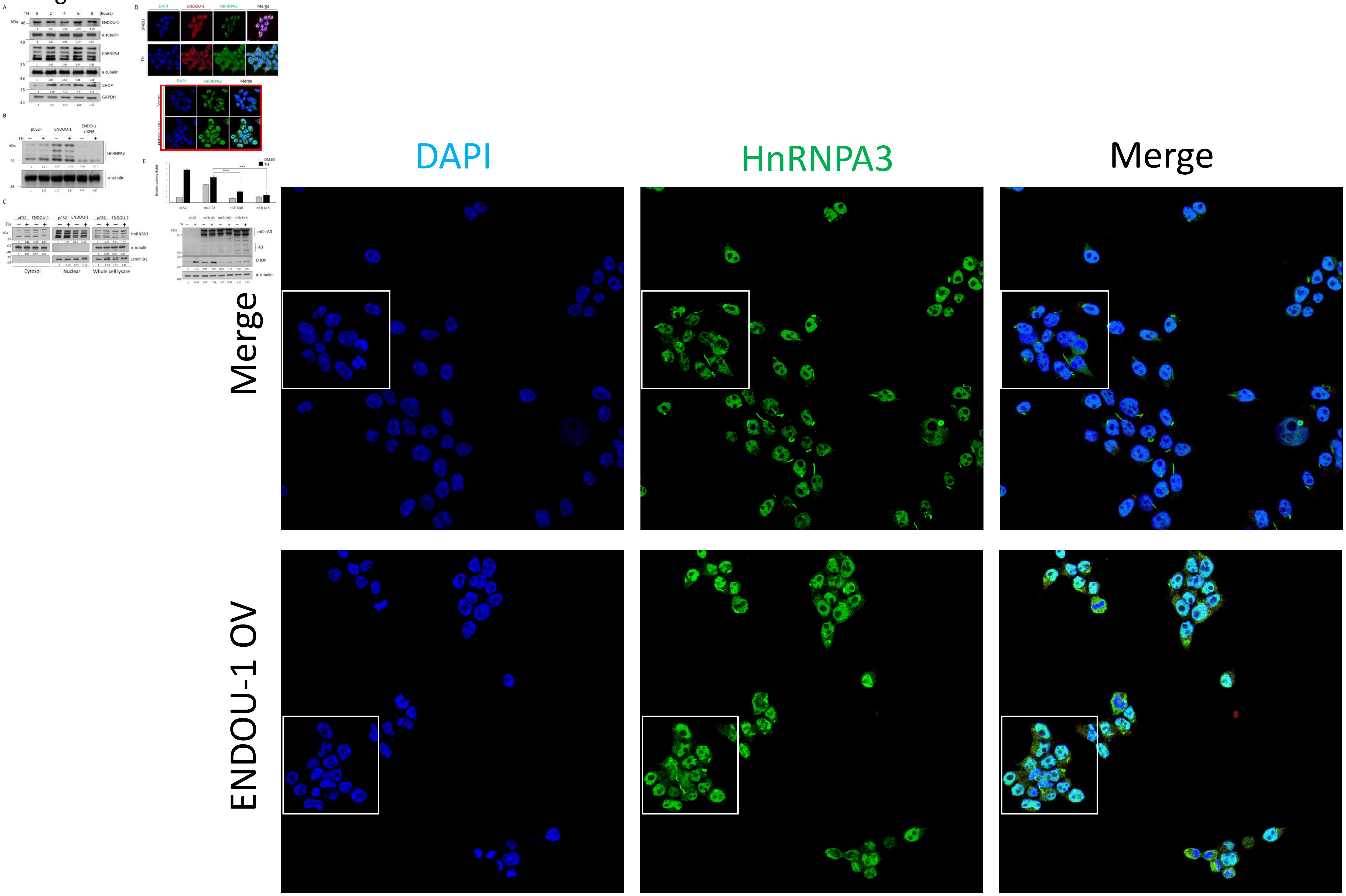

Figure S1

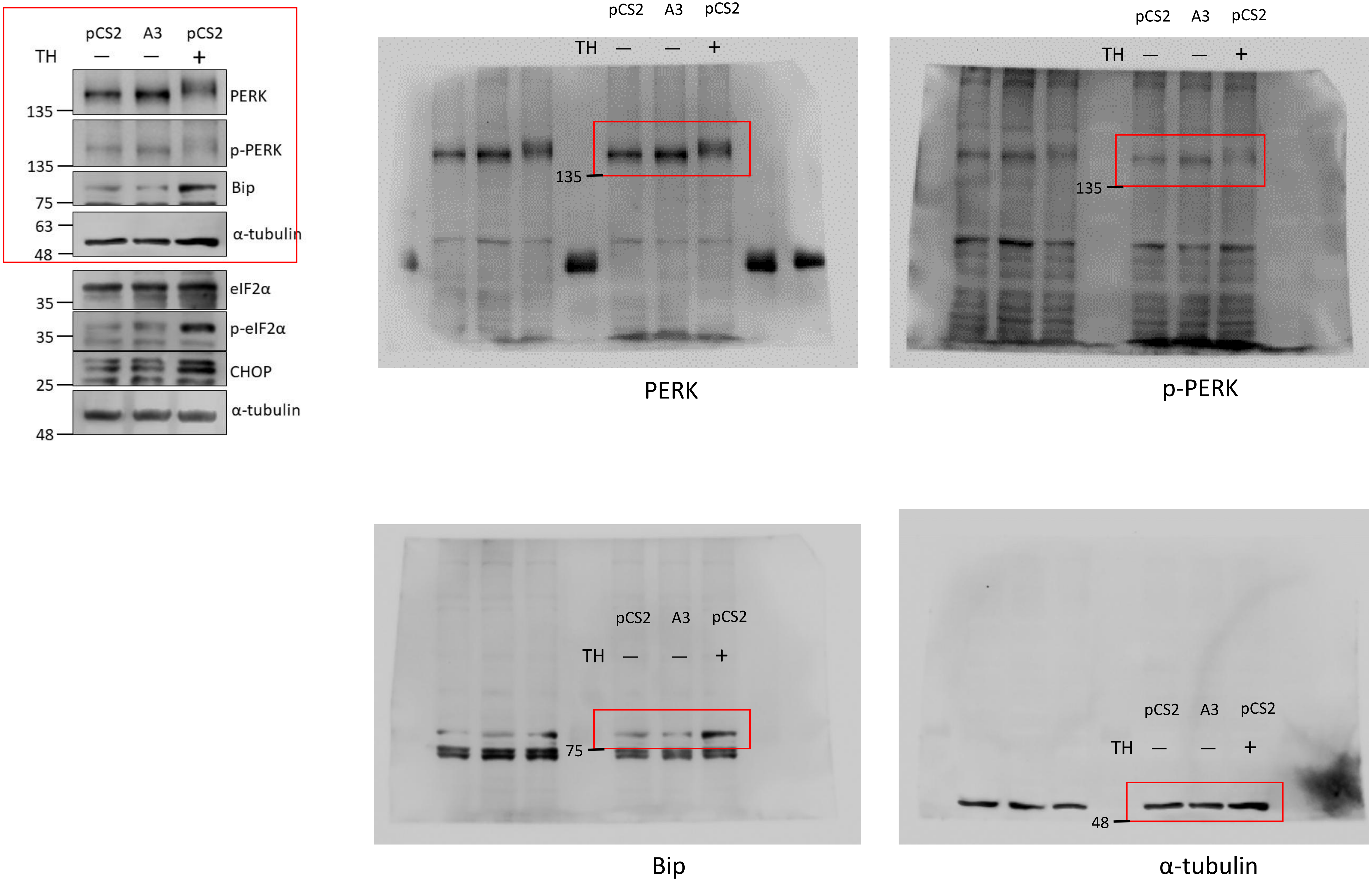

pCS2
A3
pCS2
TH
—
—
+
PERK
p-PERK
Bip
α-tubulin
eIF2α
p-eIF2α
CHOP
α-tubulin
135
135
75
63
48
35
35
25
48
PERK
p-PERK
Bip
α-tubulin

Figure S1

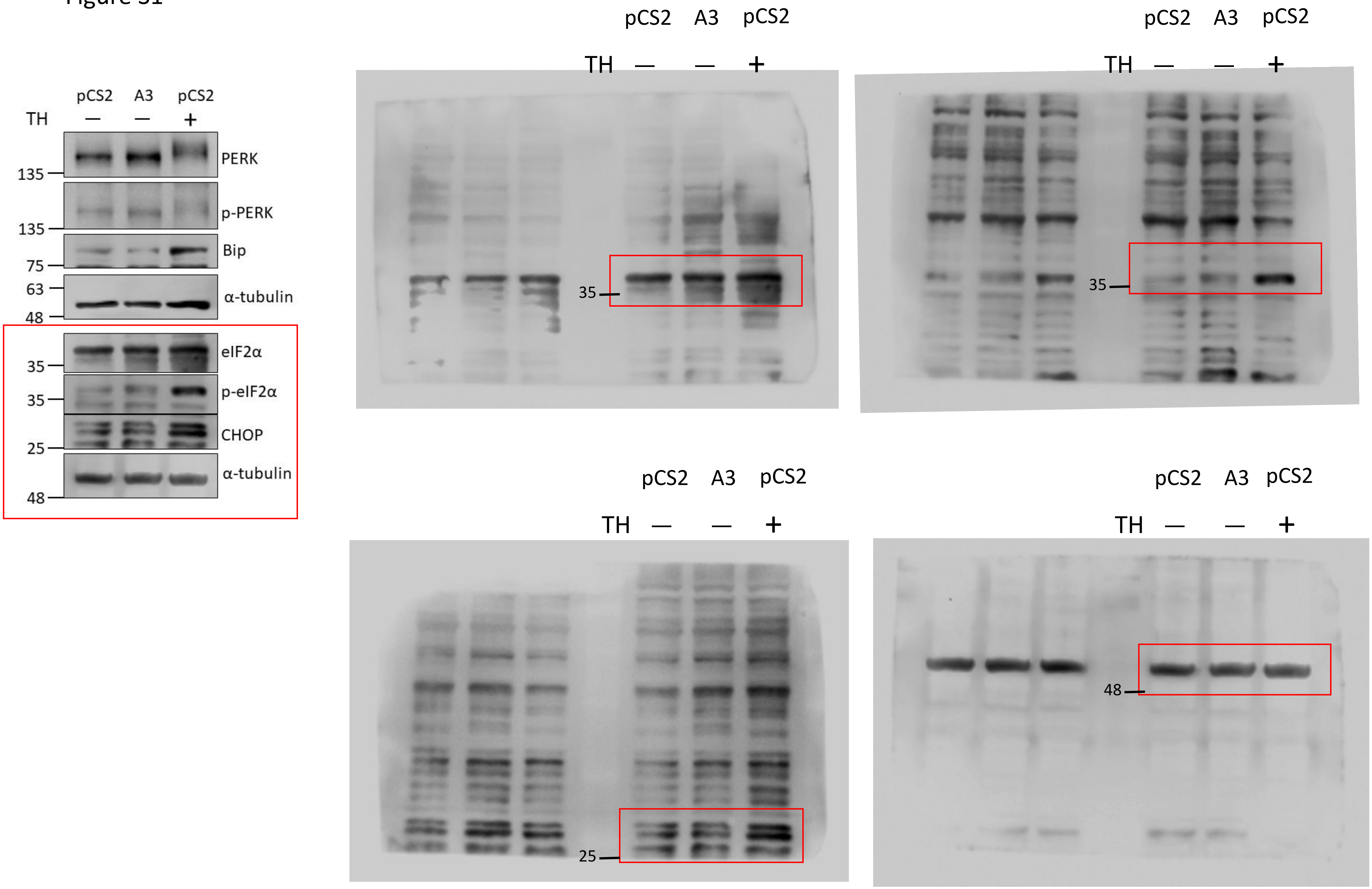
pCS2
A3
pCS2
TH
—
—
+
PERK
135
p-PERK
135
Bip
75
63
α-tubulin
48
eIF2α
35
p-eIF2α
35
CHOP
25
α-tubulin
48
35
35
25
48

Figure S3

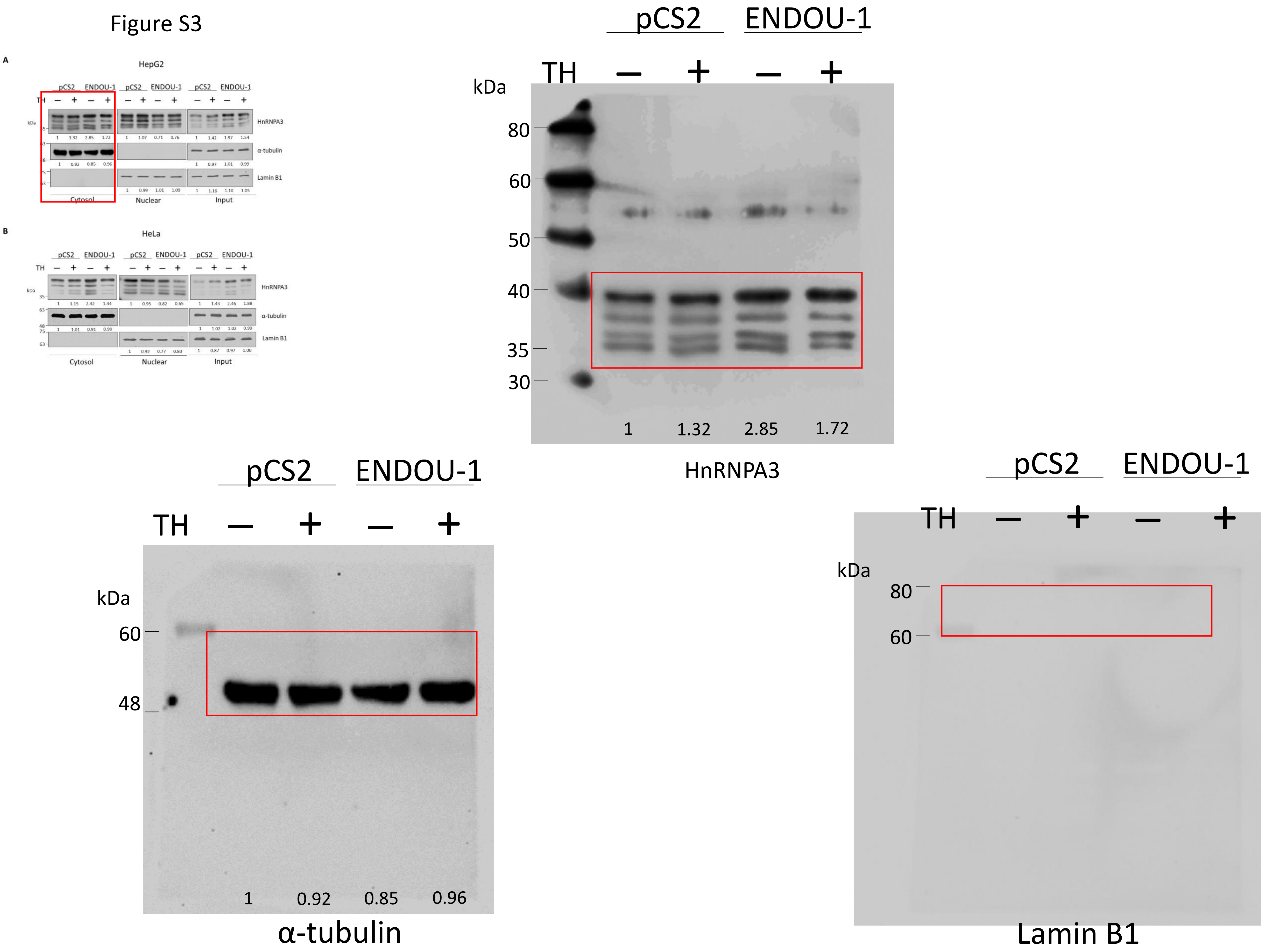
A
HepG2
pCS2
ENDOU-1
TH
HnRNPA3
α-tubulin
Lamin B1
Cytosol
Nuclear
Input
B
HeLa
kDa
80
60
50
40
35
30
1
1.32
2.85
1.72
HnRNPA3
0.92
0.85
0.96
48
α-tubulin
Lamin B1

Figure S3

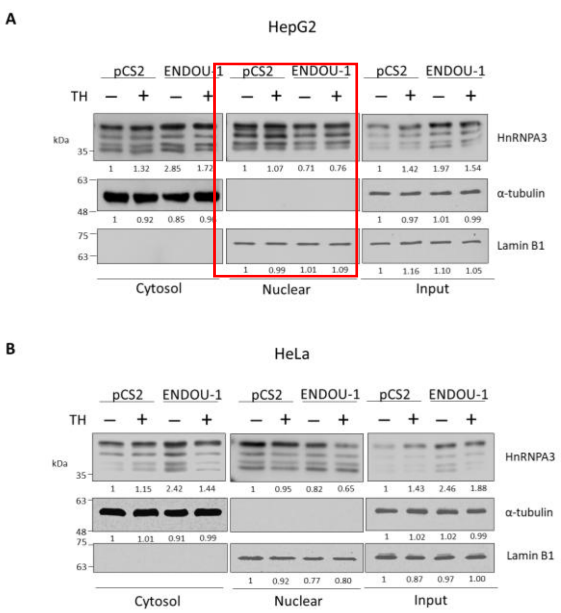

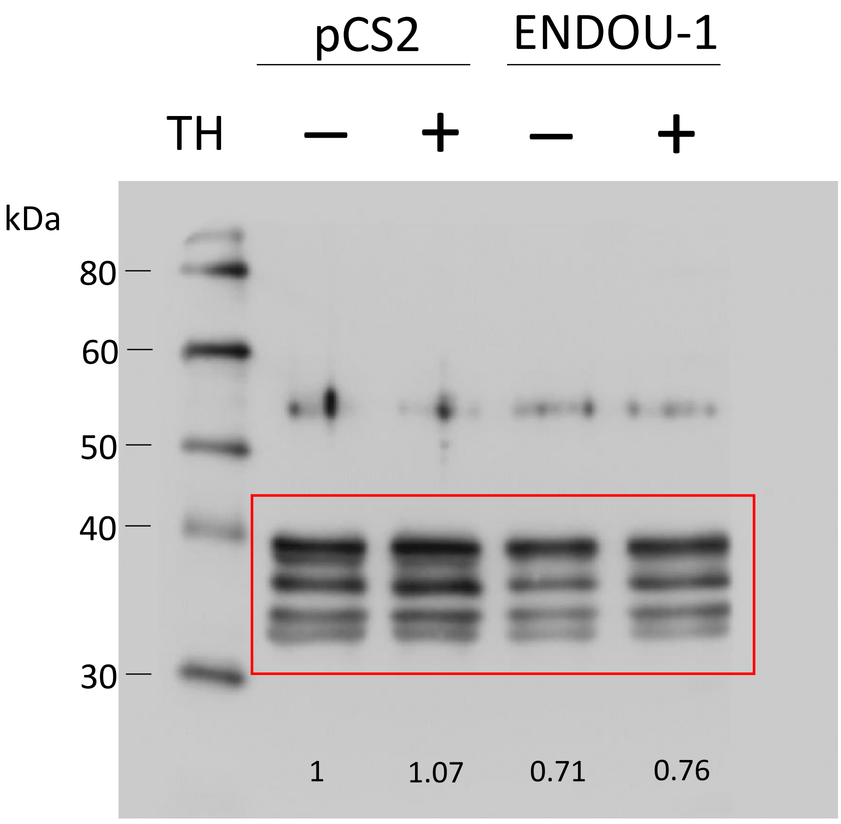

HnRNPA3

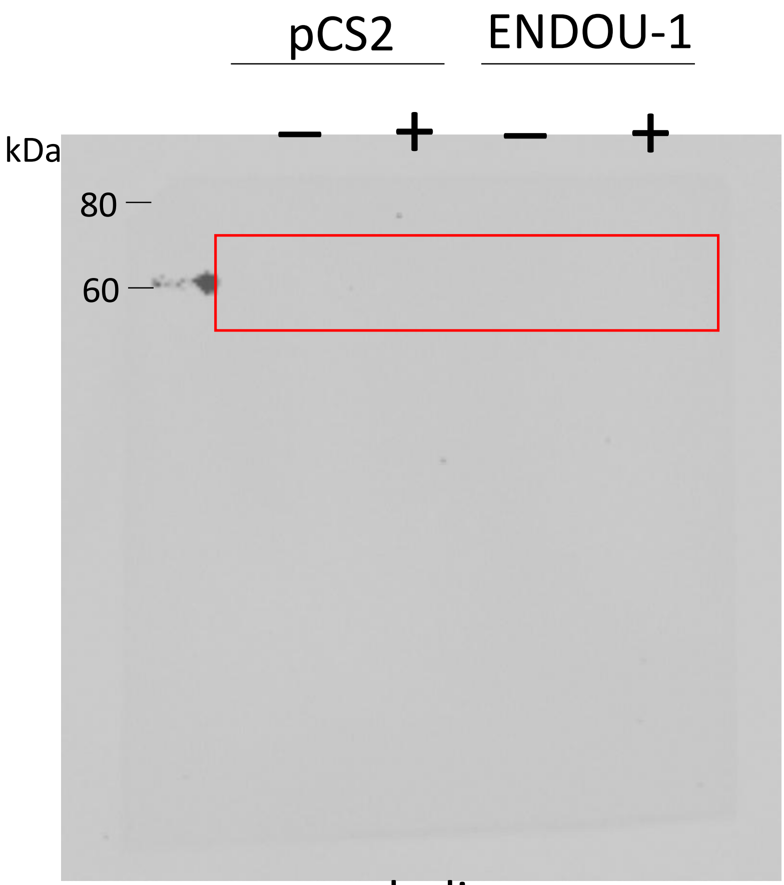

α-tubulin

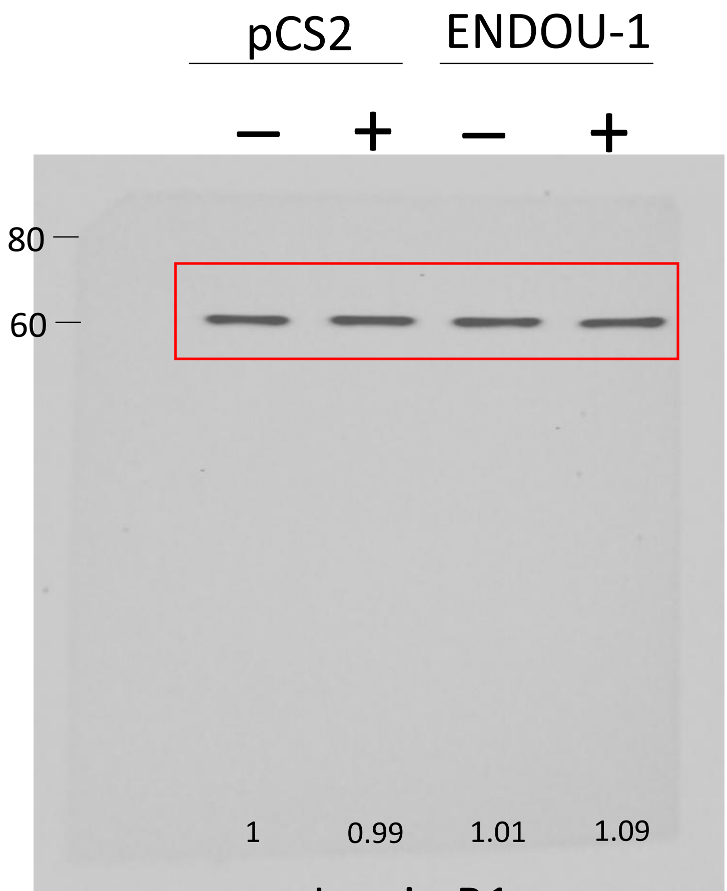

Lamin B1

Figure S3

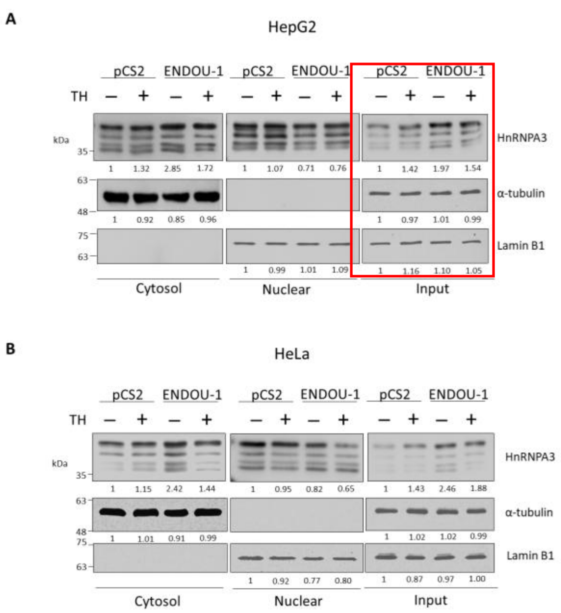

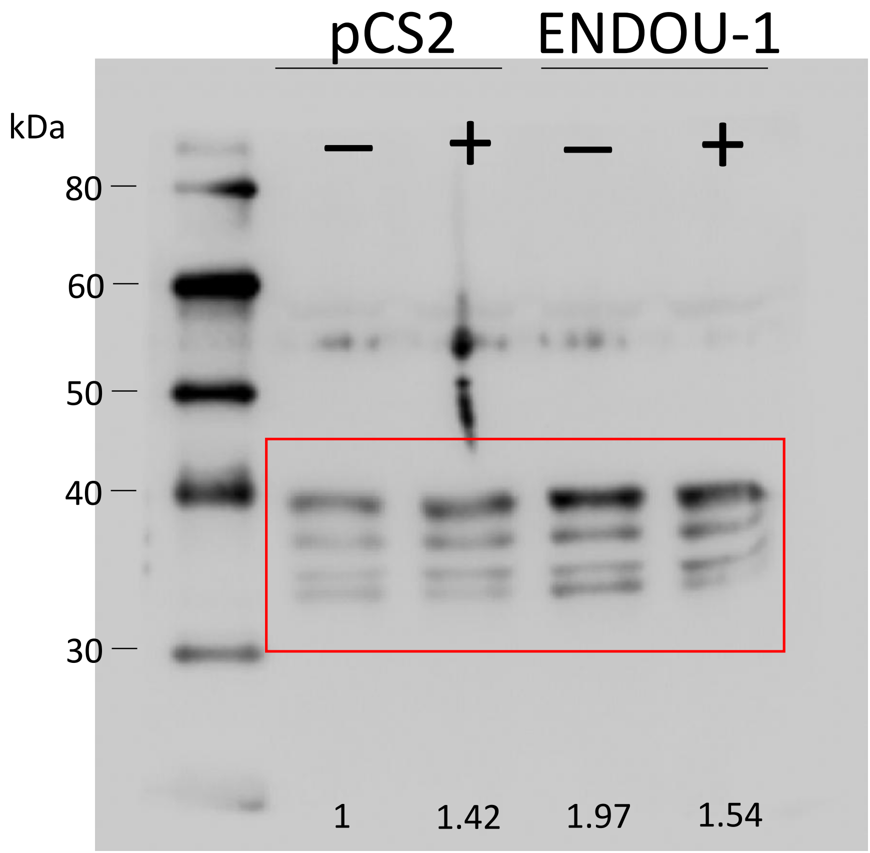

HnRNPA3

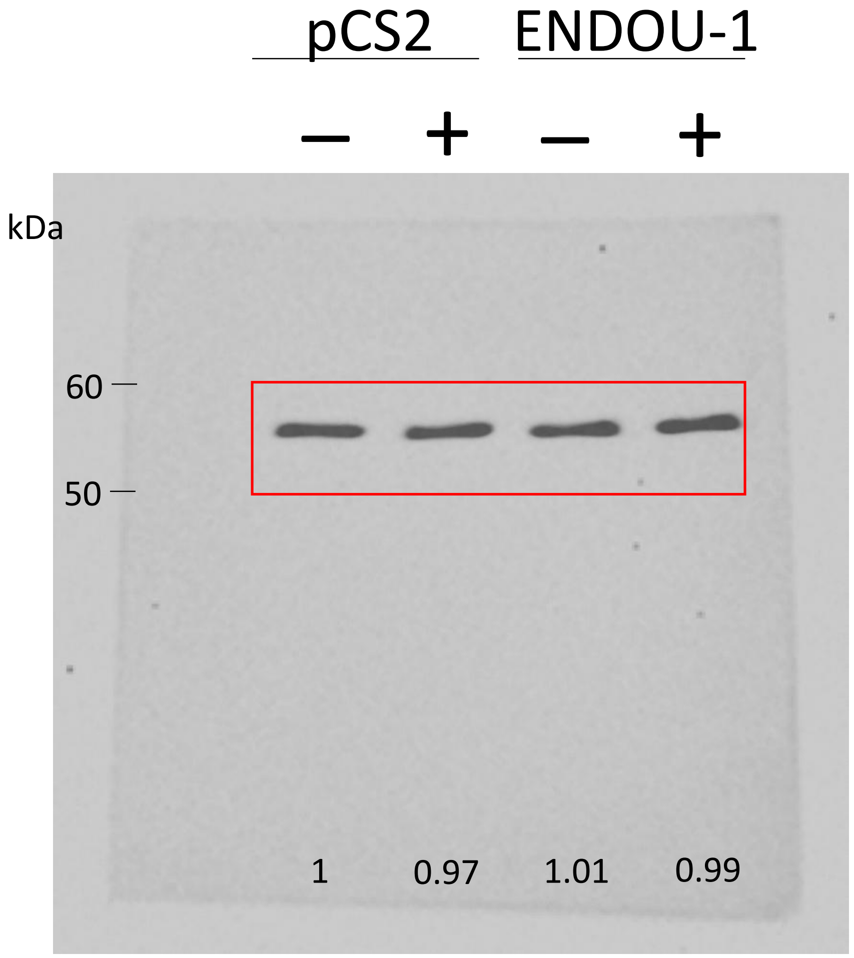

α-tubulin

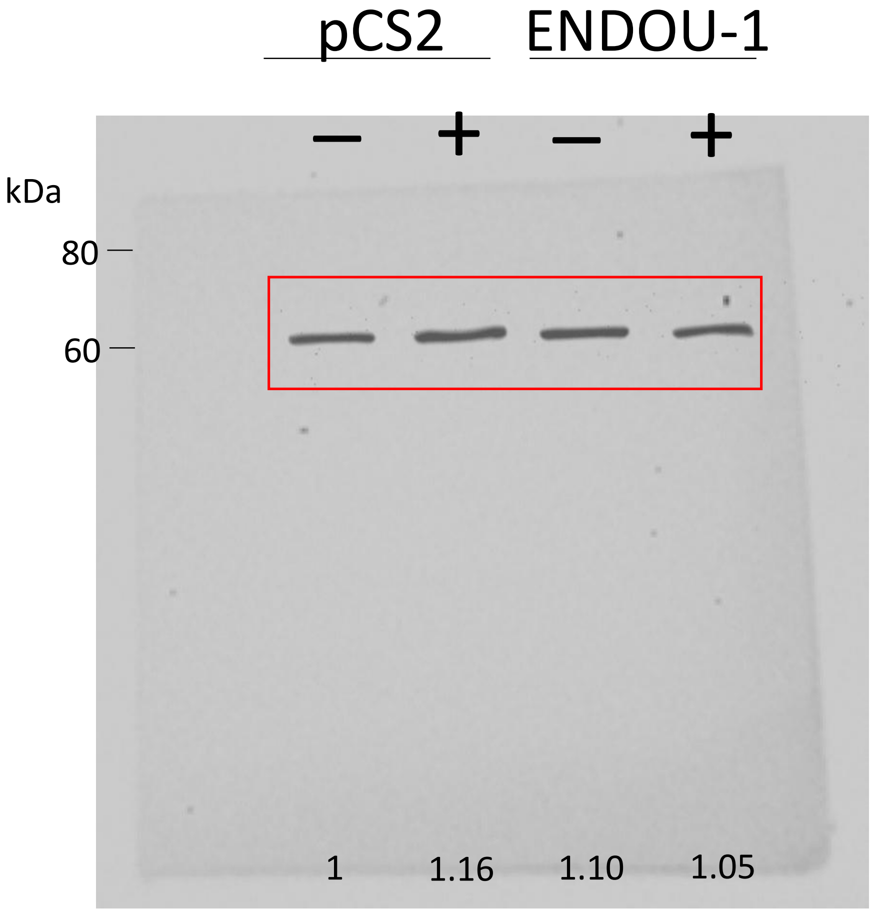

Lamin B1

Figure S3

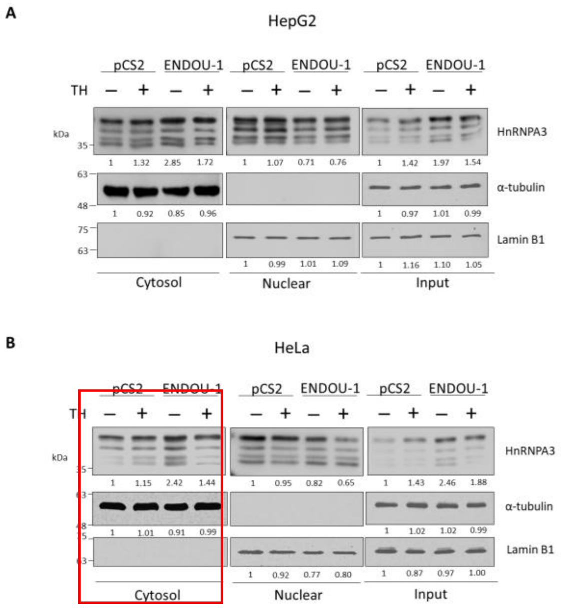

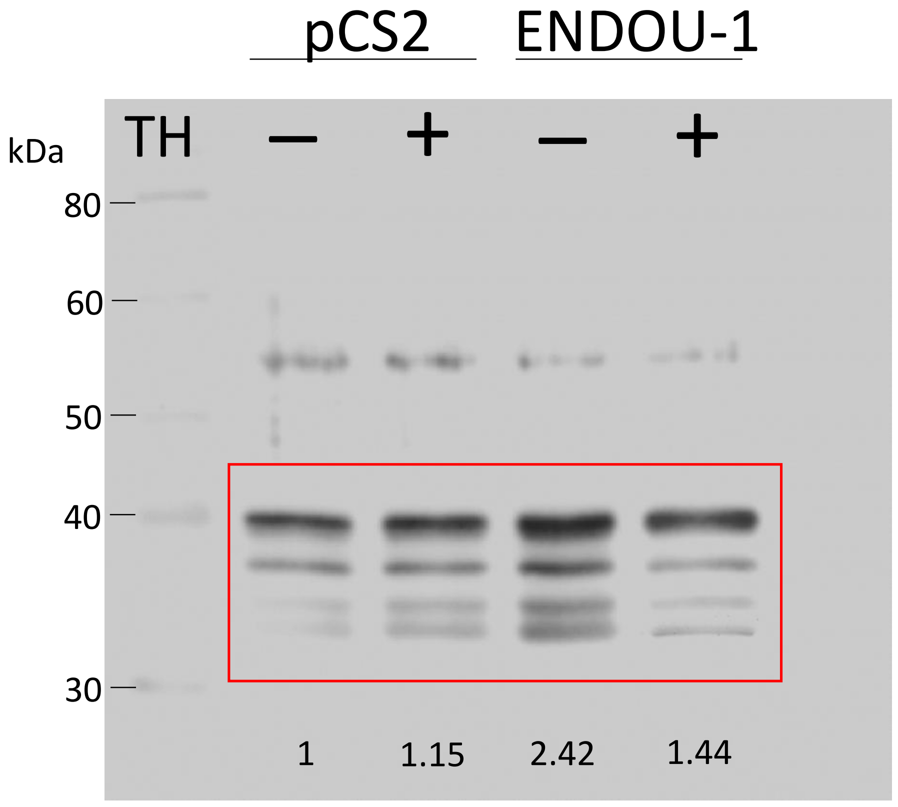

HnRNPA3

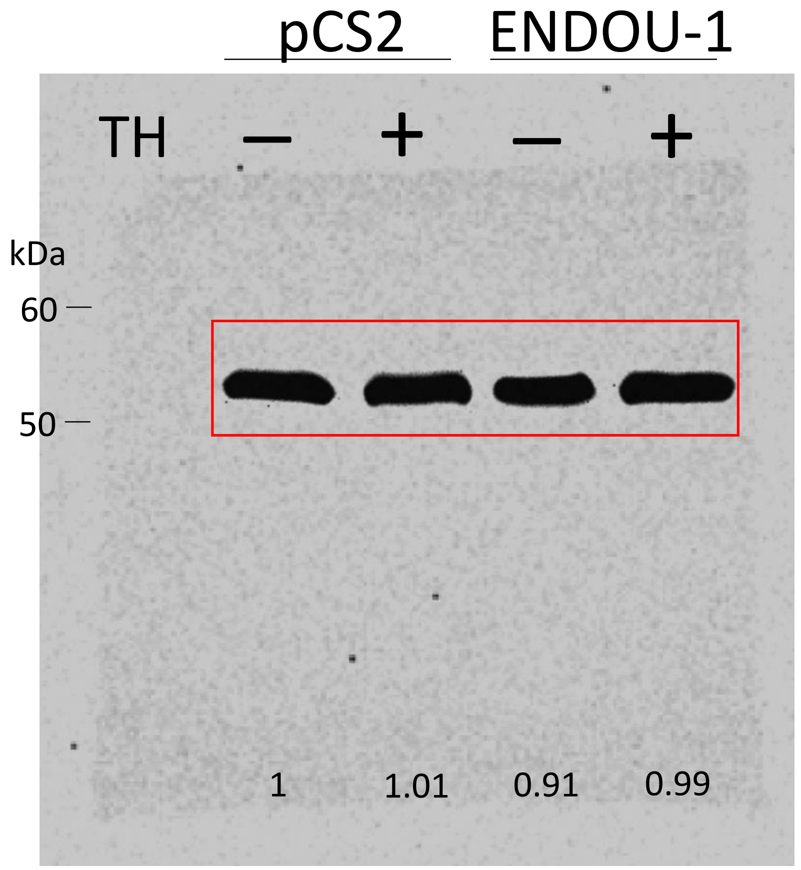

α-tubulin

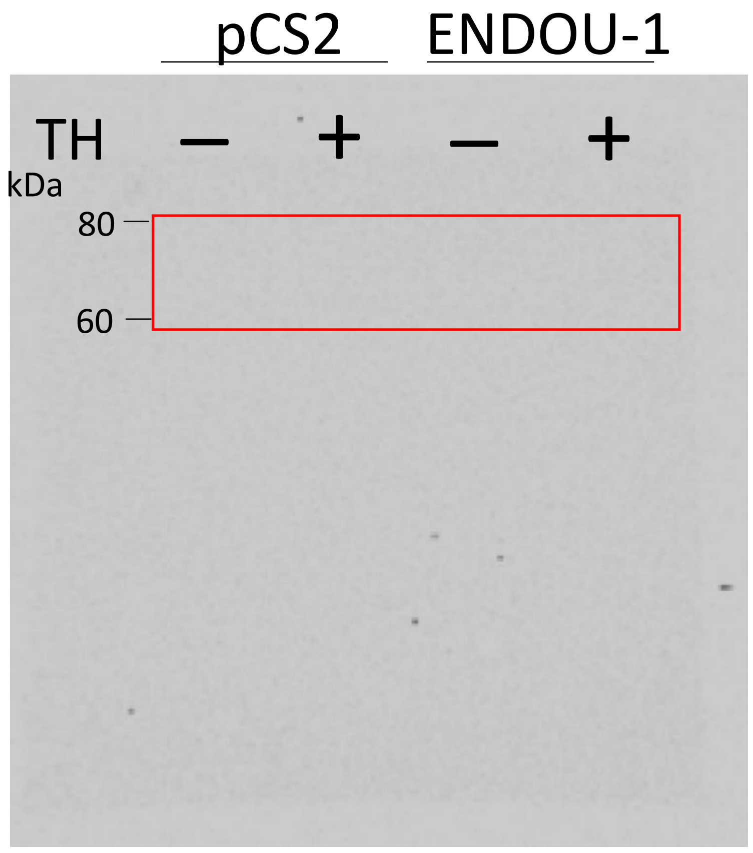

Lamin B1

Figure S3

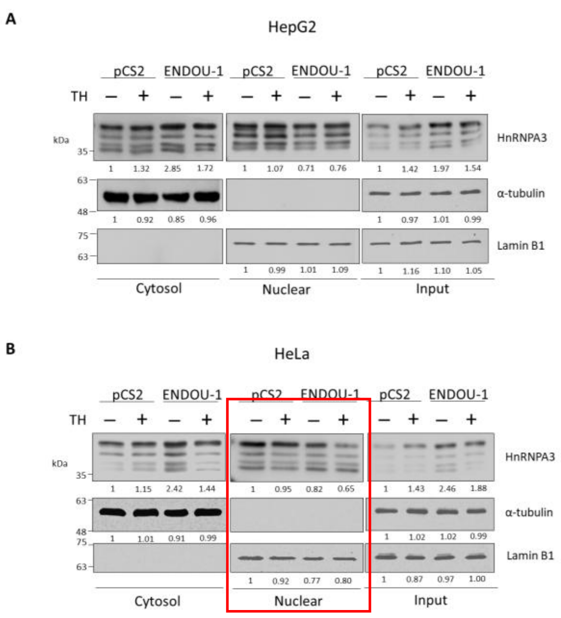

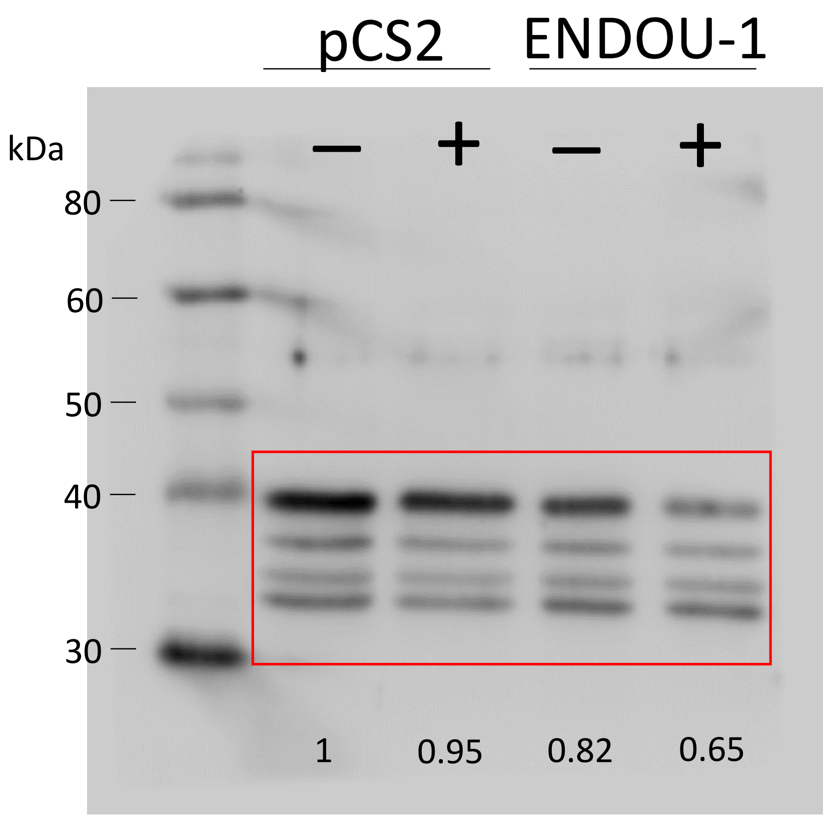

HnRNPA3

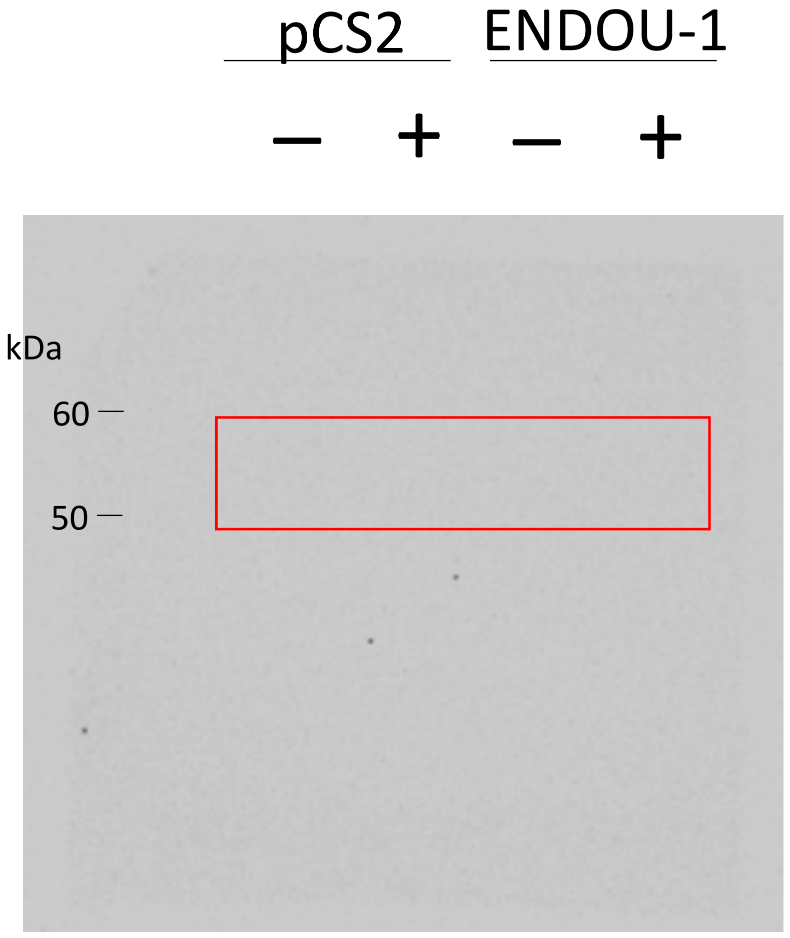

α-tubulin

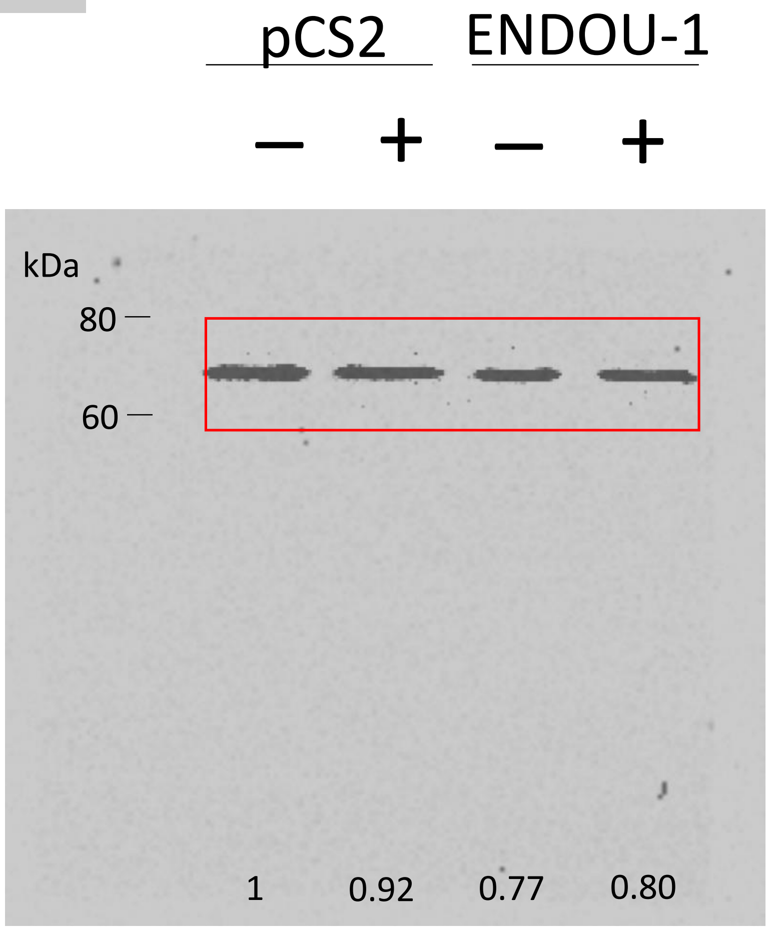

Lamin B1

Figure S3

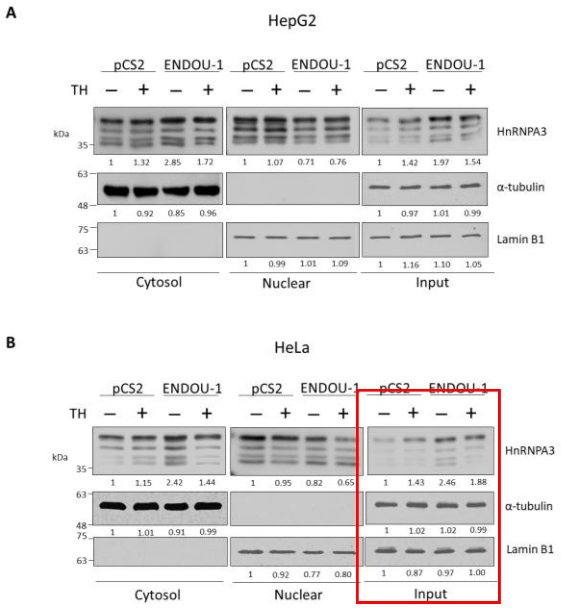

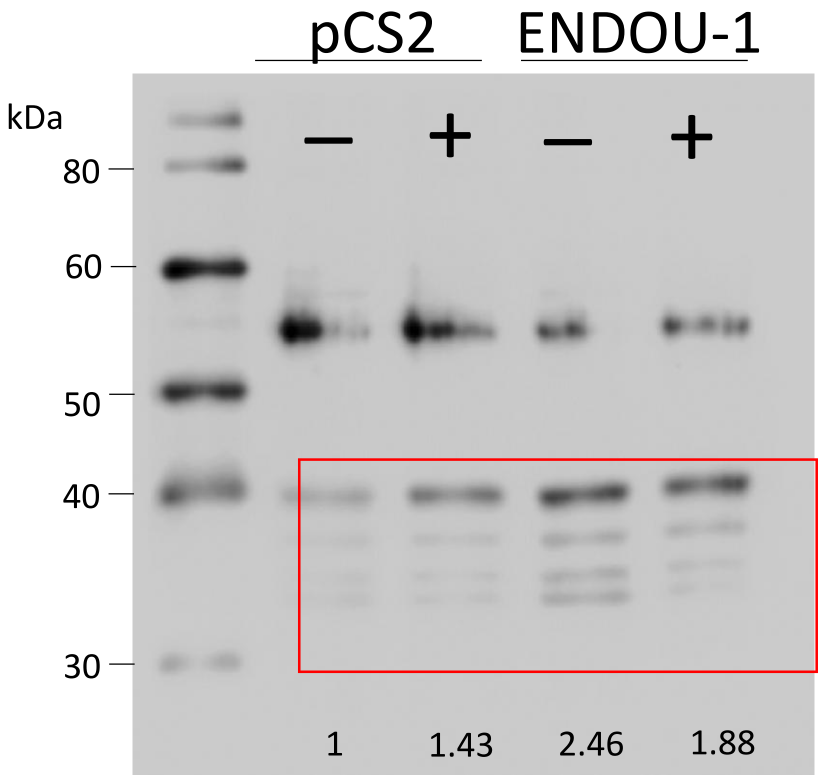

HnRNPA3

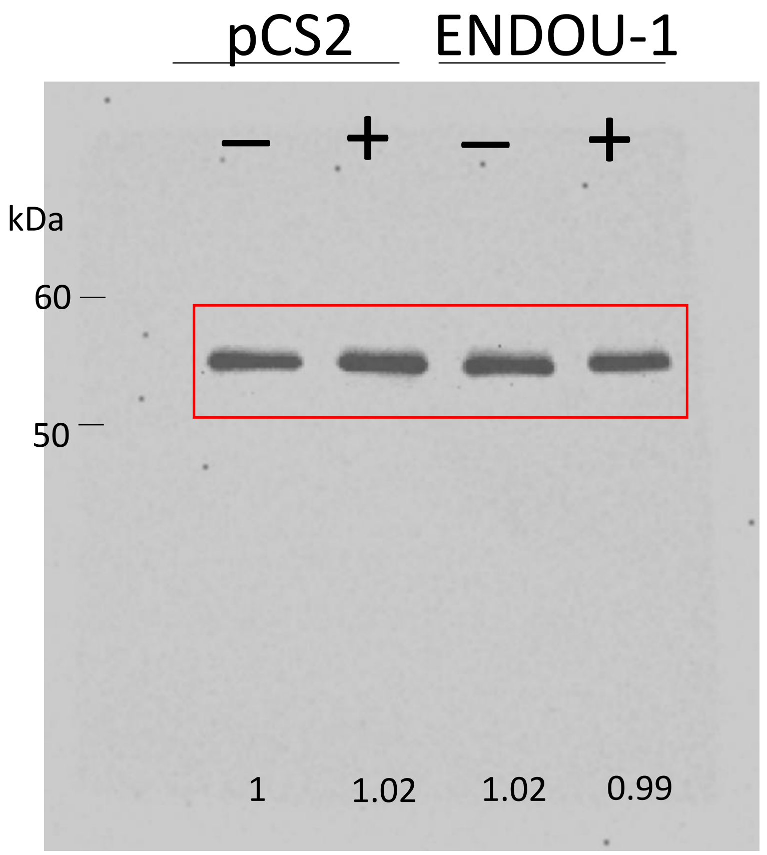

α-tubulin

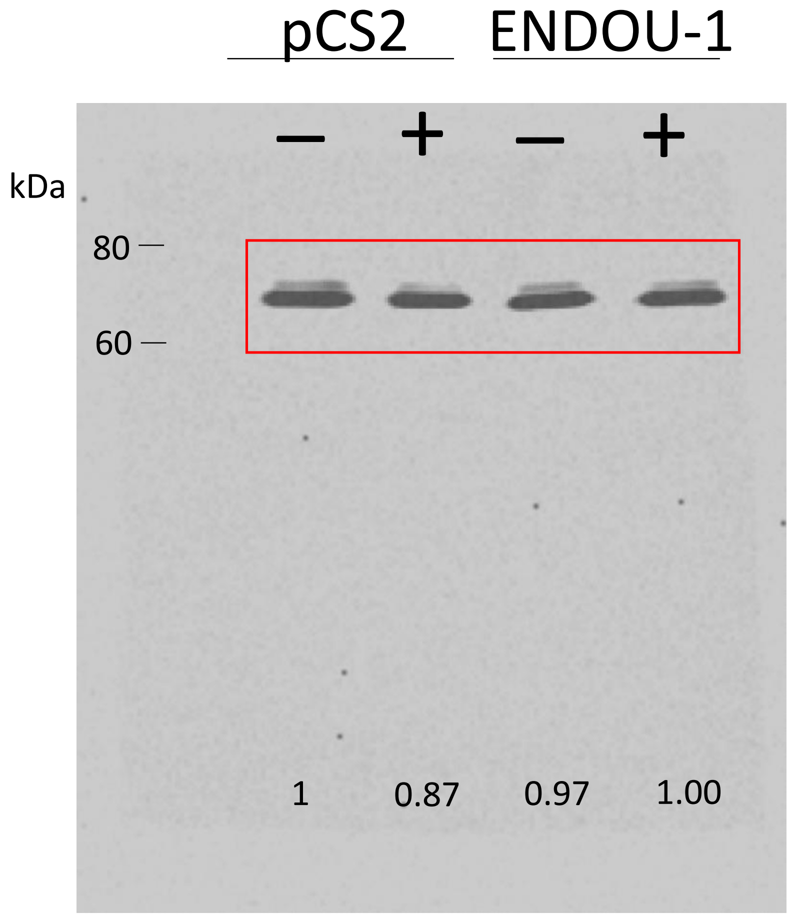

Lamin B1

Figure S6

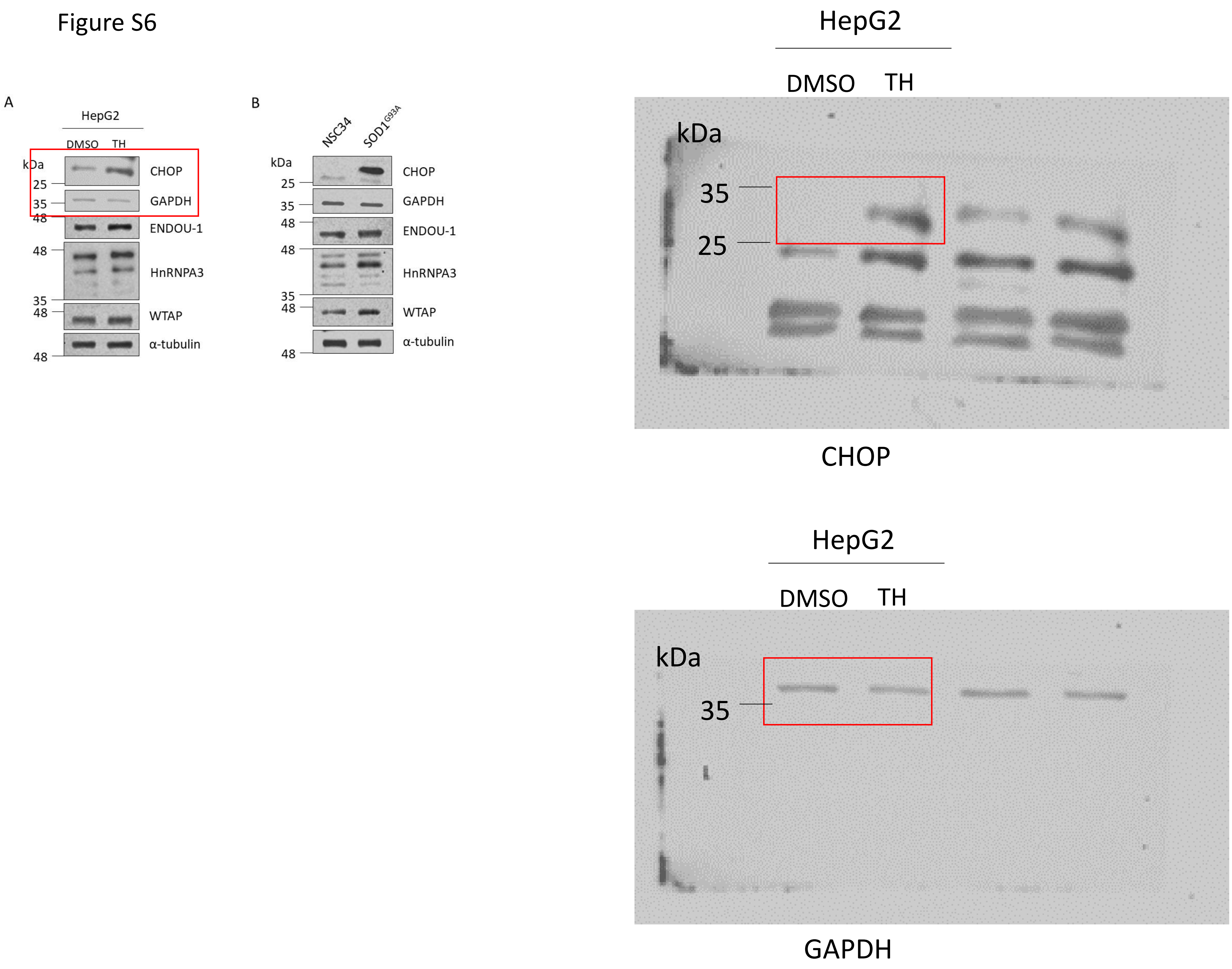

A
HepG2
DMSO
TH
kDa
25
35
48
48
35
48
48
CHOP
GAPDH
ENDOU-1
HnRNPA3
WTAP
α-tubulin
B
NSC34
SOD1G93A
kDa
25
35
48
48
35
48
48
CHOP
GAPDH
ENDOU-1
HnRNPA3
WTAP
α-tubulin
HepG2
DMSO
TH
kDa
35
25
CHOP
HepG2
DMSO
TH
kDa
35
GAPDH

Figure S6

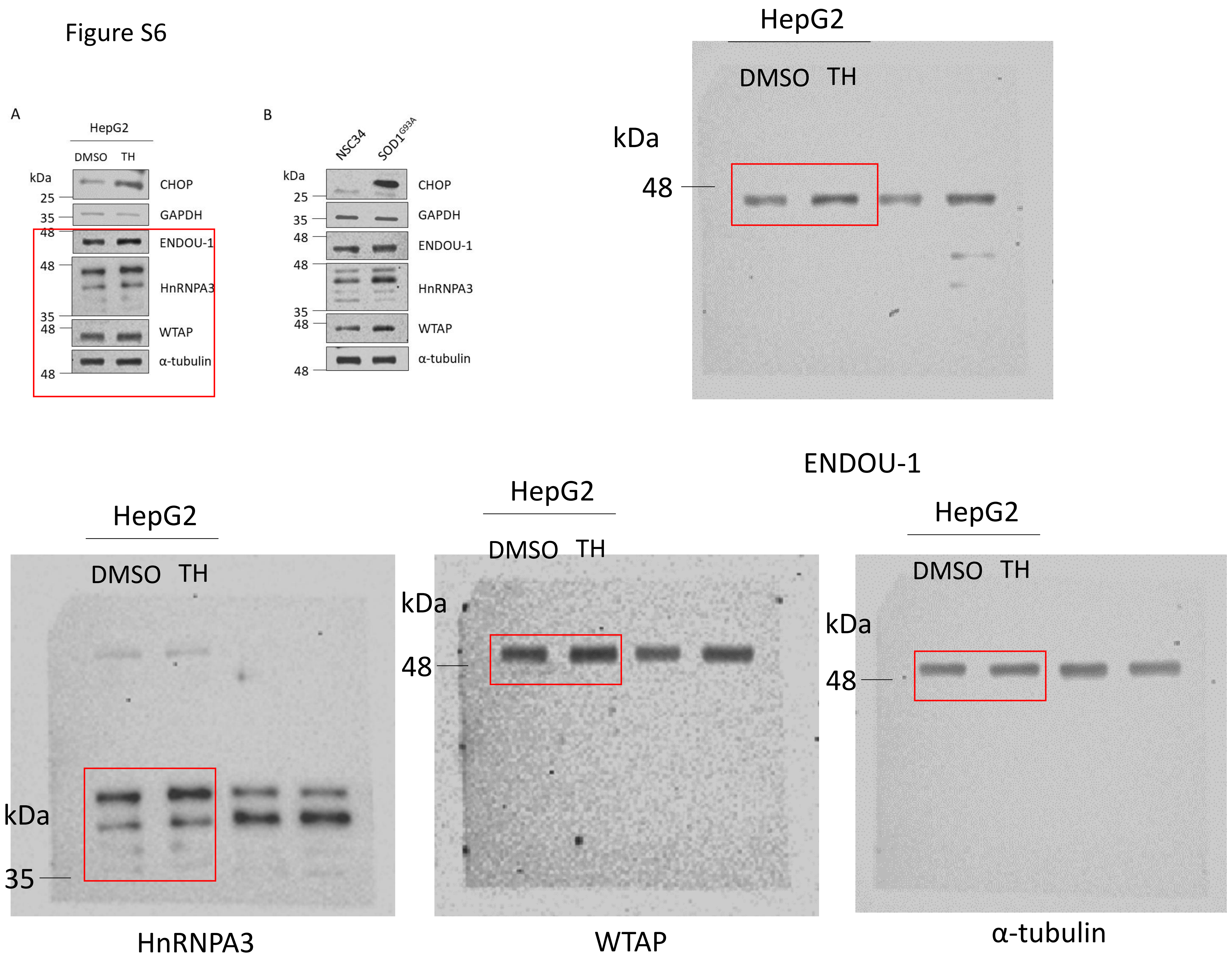
A
HepG2
DMSO TH
kDa
25
35
48
48
35
48
48
CHOP
GAPDH
ENDOU-1
HnRNPA3
WTAP
α-tubulin
B
NSC34
SOD1 G93A
kDa
25
35
48
48
35
48
48
CHOP
GAPDH
ENDOU-1
HnRNPA3
WTAP
α-tubulin
HepG2
DMSO TH
kDa
48
ENDOU-1
HepG2
DMSO TH
kDa
35
HnRNPA3
HepG2
DMSO TH
kDa
48
WTAP
HepG2
DMSO TH
kDa
48
α-tubulin

Figure S6

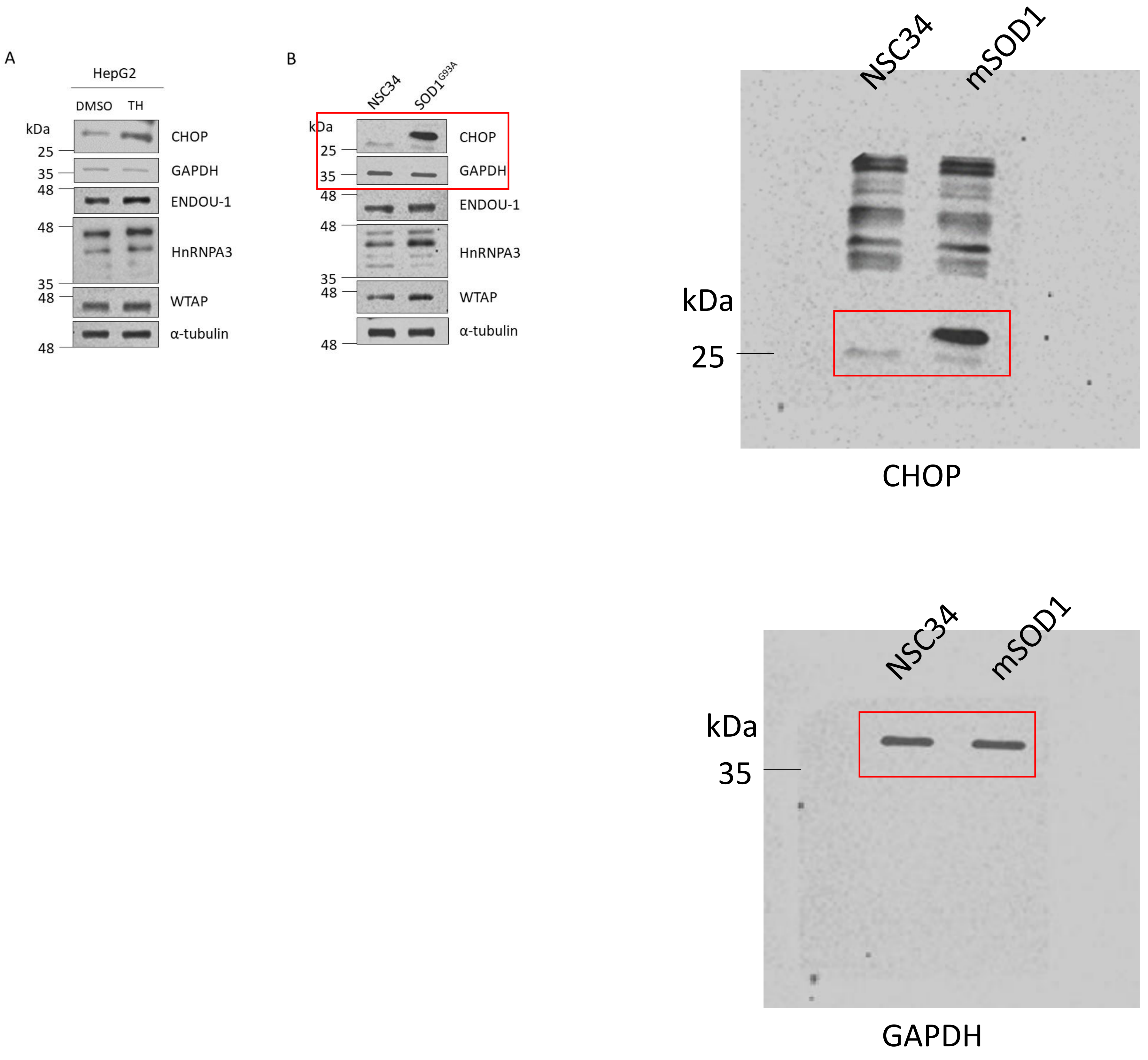
A
HepG2
DMSO
TH
kDa
25
35
48
48
35
48
48
CHOP
GAPDH
ENDOU-1
HnRNPA3
WTAP
α-tubulin
B
NSC34
SOD1G93A
kDa
25
35
48
48
35
48
48
CHOP
GAPDH
ENDOU-1
HnRNPA3
WTAP
α-tubulin
NSC34
mSOD1
kDa
25
CHOP
NSC34
mSOD1
kDa
35
GAPDH

Figure S6

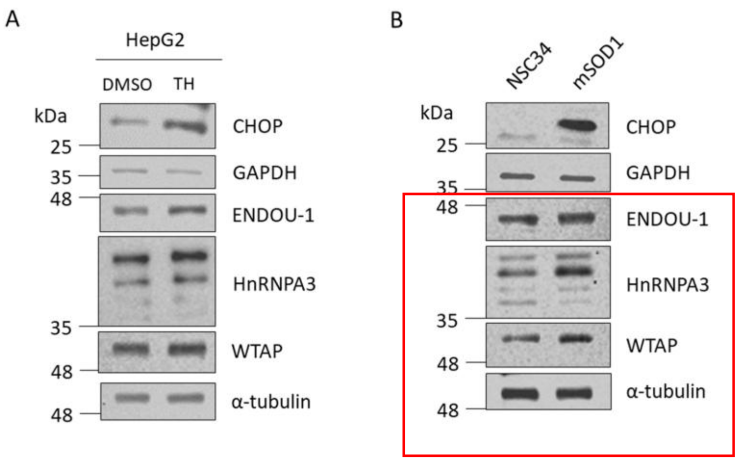

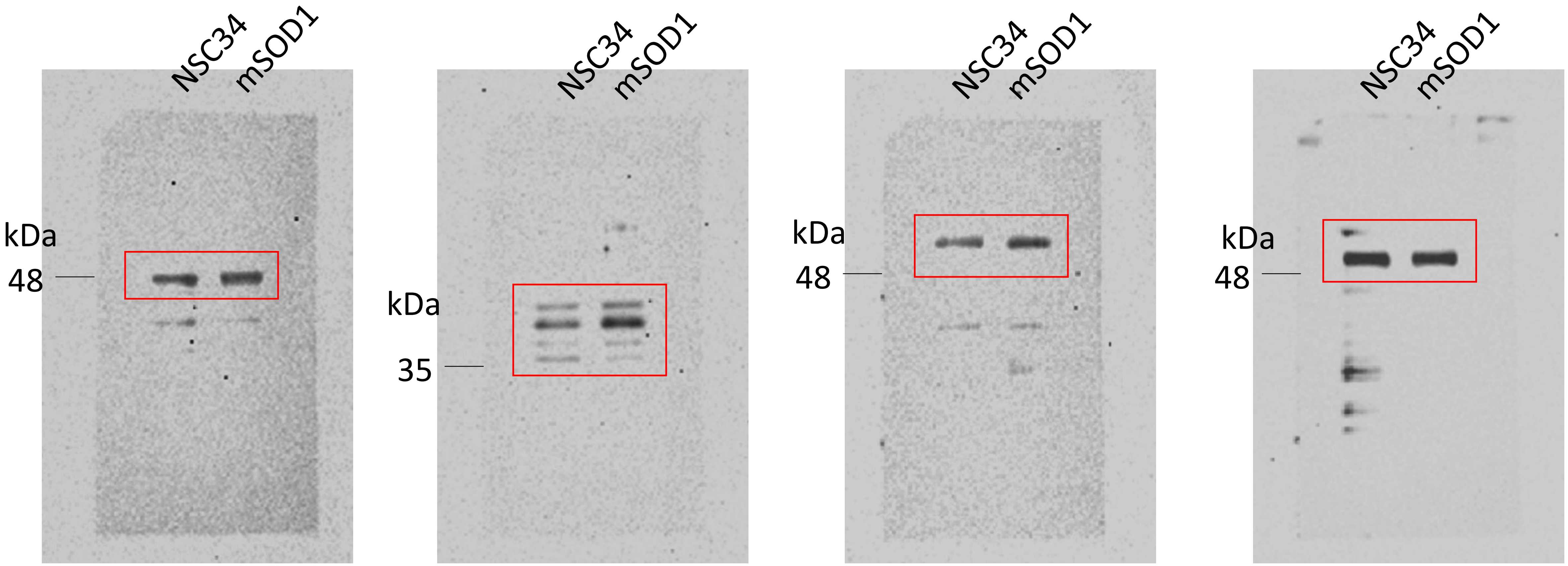

ENDOU-1 HnRNPA3 WTAP α-tubulin
